# Synthesis of Three-Dimensional Ring Fused Heterocycles by a Selective [4 + 2] Cycloaddition Between Bicyclic Thiazolo 2-Pyridones and Arynes

**DOI:** 10.1021/acs.joc.3c01957

**Published:** 2023-12-14

**Authors:** Souvik Sarkar, Pardeep Singh, Simon Edin, Ola F. Wendt, Fredrik Almqvist

**Affiliations:** † Department of Chemistry, Umeå University, 901 87 Umeå, Sweden; ‡ Centre for Analysis and Synthesis, Lund University, SE-221 00 Lund, Sweden

## Abstract

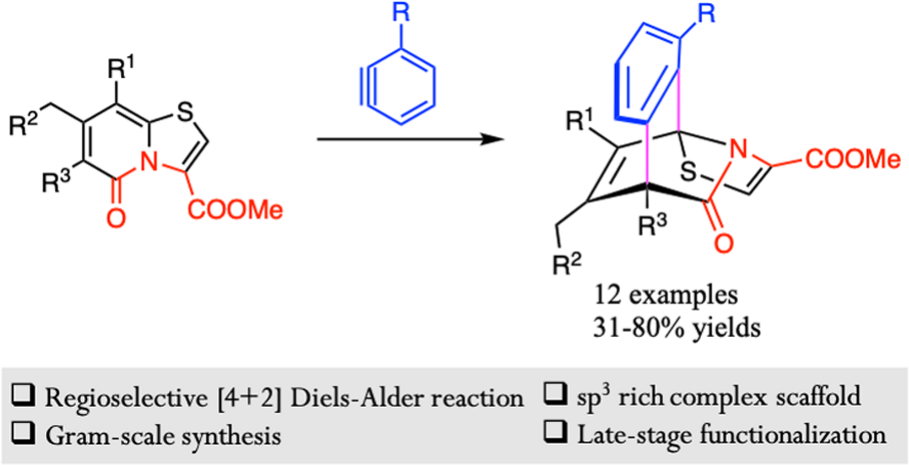

A selective [4 + 2] cycloaddition reaction of thiazolo-2-pyridones with arynes has been demonstrated. The developed protocol allows rapid access to highly functionalized, structurally complex thiazolo-fused bridged isoquinolones in high yields, which are susceptible to further late-stage functionalization.

Cycloaddition reactions are one of the most efficient and atom economical strategies to build complex ring systems.^1−5^ The ring systems of a scaffold are key components to determine the properties like three-dimensionality, rigidity, polarity, metabolic stability and toxicity.^6,7^ The scaffolds composed of fused 2*D*/3D fragments are important in medicinal chemistry due to their unique structure and often improved physicochemical properties.^8^ For instance, MK-8866, Huperzine A and varenicline (Figure 1A), based on fused 2*D*/3D ring systems, have been marketed to treat Alzheimer’s disease, type 2 diabetes, and smoking addiction, respectively.^9−11^

**Figure 1:**
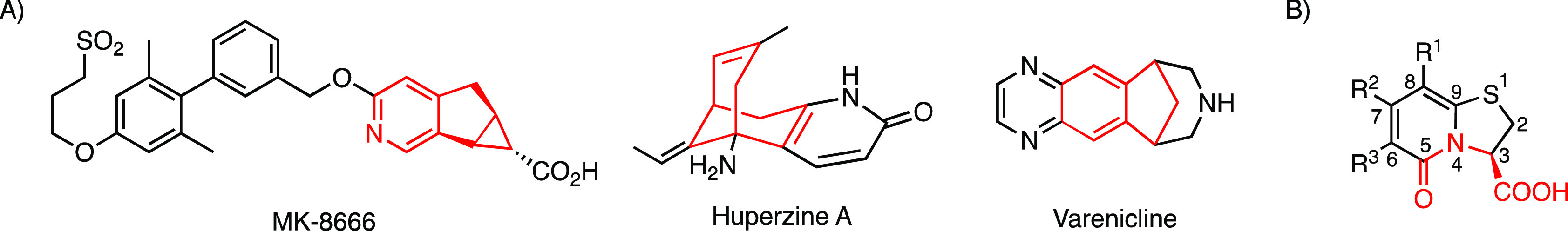
(A) Examples of bioactive compounds and natural products with fused 2D/3D rings. (B) Structure of thiazolino fused 2-pyridone.

The thiazolino fused 2-pyridone (Figure 1B) is an illustrious peptidomimetic scaffold, the derivatives of which exhibit a broad spectrum of biological activities.^12−16^ The bicyclic scaffold has also been used as a template to construct various heterocycles of diverse shapes.^17−19^ The compounds embodying carbo and heterocyclic rings around the thiazolino-2-pyridone scaffold (Figure 1B) has been shown to exhibit interesting biological activities.^20^ In our previous report, we showed that the cyclic sulfide in the thiazoline ring and the 4π system in the 2-pyridone ring **1** compete for their reaction with arynes, resulting in a mixture of *N*-alkenyl-2-pyridone **2** and the Diels–Alder adduct **3** (Scheme 1A). A selective [4 + 2] cycloaddition reaction across the 2-pyridone ring remained elusive due to the inherent nucleophilicity of the cyclic sulfide. The Diels–Alder adduct **3** can be viewed upon as an interesting sp^3^ rich, structurally complex peptidomimetic scaffold, that could be derivatized to compounds of biological interest.

**Scheme 1:**
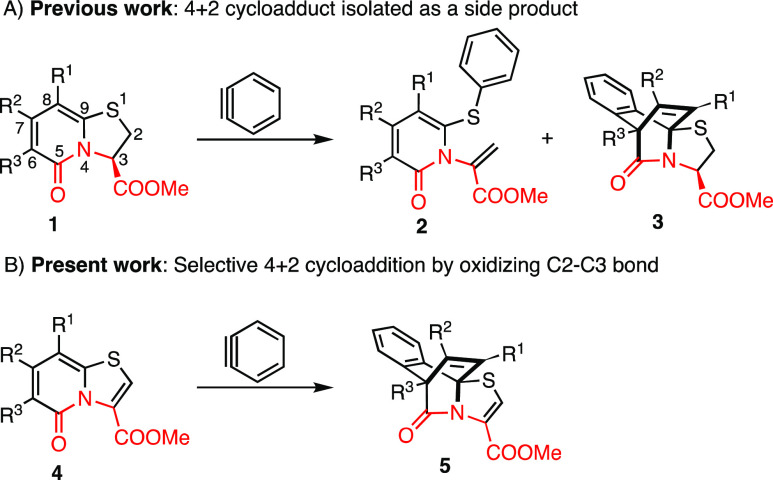
Diversification of Peptidomimetic Thiazolino-2-pyridones by Their Reaction with Arynes (A) Previous work. Aryne induced ring opening and [4 + 2] cycloadduct **3** as a side product. (B) This work. The oxidation of C2–C3 bond results in a selective [4 + 2]cycloaddition reaction across the 2-pyridone ring.

We wanted to change the characteristics of the thiazolino-2-pyridone scaffold in a manner that would favor the Diels–Alder reaction across the 2-pyridone ring by making the cyclic sulfide less reactive toward the aryne. Cognizant that an acidic proton at C3 is mandatory for the thiazolino-2-pyridone to undergo ring opening,^21^ we further hypothesized that oxidation of the C2–C3 bond would not only eliminate the possibility of ring-opening but it would also reduce the nucleophilicity of the cyclic sulfide by engaging it in conjugation with the α,β-unsaturated methyl ester. As a consequence, the [4 + 2] cycloaddition would be favored, resulting in a new substituted and functionalized ring-fused scaffold with 2D/3D character and great potential for late-stage modifications (Scheme 1B).

In continuation of our interest to expand the chemical space around the thiazolino-2-pyridone scaffold and generate new central fragments with more 3D character, we herein present an efficient, selective, scalable, and broad scope Diels–Alder reaction of thiazolo-2-pyridones with arynes for the synthesis of highly functionalized thiazolo-fused bridged isoquinolones comprised of a 2*D*/3D fused ring system.

We began our investigations by testing the reaction of thiazolino-2-pyridone (**6a**) with 2-(trimethylsilyl)phenyl trifluoromethanesulfonate (**7a**) using the conditions published previously.^21^ To our delight, the desired Diels–Alder adduct (**8a**) was obtained in 64% yield (entry 1) when **6a** was allowed to react with **7a** in the presence of KF and 18-crown-6 in THF at room temperature for 16 h (Table 1). Other fluoride sources such as CsF (entry 2) and tetrabutylammonium fluoride (TBAF, entry 3) provided **8a** in low yields, but the use of tetrabutylammonium difluorotriphenylsilicate (TBAT, entry 4) gave results comparable to KF. However, opting for the less expensive reagent, we chose the KF and 18-Crown-6 combination over TBAT for further optimization. When the reaction was carried out at 60 °C for 16 h, the yield of **8a** could be improved to 75% (entry 5). Further, different solvents were screened (Table S1, SI) in order to improve the yield and reduce the reaction time. The reaction in MeCN using 1.5 equiv of **7a** furnished **8a** in 68% yield when heated at 60 °C (entry 6) for 15 min. Finally, increasing the amount of aryne precursor **7a** to 2.0 equiv and employing 2.5 and 3.0 equiv of KF and 18-crown-6 respectively, yielded **8a** in 76% in 15 min (entry 7).

**Table 1:**
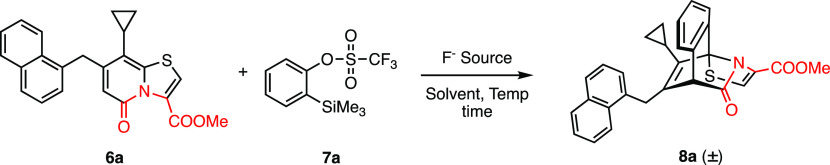
Optimization of Reaction Conditions for Selective [4 + 2] Cycloaddition

entry	F^–^ source (equiv)	18-c-6 equiv	**7a** equiv	time	solvent	temp (°C)	yield^a^ (%)
1	KF (2.0)	2.5	1.5	16h	THF	RT	64
2	CsF (2.0)		1.5	16h	THF	RT	11
3	TBAF (2.0)		1.5	16h	THF	RT	6
4	TBAT (2.0)		1.5	16h	THF	RT	61
5	KF (2.0)	2.5	1.5	16h	THF	60	75
6	KF (2.0)	2.5	1.5	15 min	MeCN	60	68
7	KF (2.5)	3.0	2	15 min	MeCN	60	76

a Isolated yields.

To understand the impact of the substituents on the outcome of the reaction, a set of variedly substituted ring fused 2-pyridones **6a**–**f** were tested for their reaction with arynes (Scheme 2). Ring fused 2-pyridones substituted with a methyl/1-naphthyl at position C7 in combination with groups like cyclopropyl (**6b**), methoxy (**6c)** and phenyl (**6d)** at position C8 reacted smoothly with unsubstituted aryne to give desired products **8b**–**d** in 66–80% yield. The 2-pyridone **6e** substituted with a bromo group at position C2, was compatible under the reaction conditions and gave the cycloadduct **8e** in 60% yield. The reaction of 2-pyridone **6f** bearing a benzothiophene group with an unsubstituted aryne was also successful, affording the corresponding product **8f** in 53% yield. Next, we investigated the reaction of **6a** and **6c**–**d** with electrophilic 3-methoxy aryne generated *in situ* from precursor **7b**. This aryne is more prone to react with nucleophiles due to the angle distortion as proposed by Medina et al.^22^ This effect has been proven in our previous work,^21^ when electrophilic 3-methoxy aryne reacted exclusively with the cyclic sulfide and did not participate in the [4 + 2] cycloaddition reaction. However, electrophilic 3-methoxy aryne underwent only a [4 + 2] cycloaddition reaction across the diene system in the 2-pyridone ring of **6a** and **6c**–**d** to give cycloadducts **8g**–**i** in 31–62% yield (Scheme 2). Rewardingly, the reaction of the 3-methoxy aryne with **6a** and **6c**–**d** was found to be regioselective. The structure of **8i** was unambiguously confirmed with the help of X-ray diffraction analysis.

**Scheme 2:**
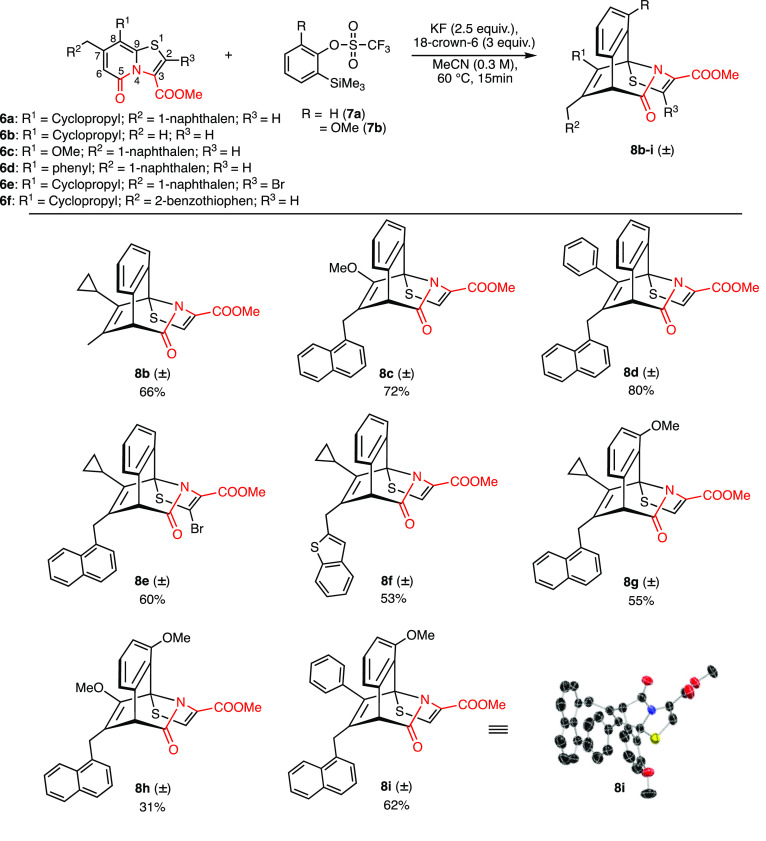
Reaction of C2–C3 Oxidized Ring Fused 2-Pyridones **6a**–**f** with In Situ Generated Arynes and Crystal Structure of **8i** Showing Thermal Ellipsoids at the 50% Probability Level (CCDC 2113369, See SI for Details)

It has been shown that the steric and electronic nature of the substituents at position C6 of the thiazolino-2-pyridone plays an important role to determine the selectivity of their reaction with arynes.^21^ Thus, to investigate the effect of substituents and explore the substrate scope of the [4 + 2] cycloaddition reaction, thiazolo-2-pyridones **9a**–**c** substituted with −Br, −NO_2_, and −NH_2_ at C6 were tested for their reaction with aryne (Scheme 3). Gratifyingly, the reaction of 6-bromo-thiazolo-2-pyridone **9a** with aryne generated in situ from **7a**, resulted in the desired product **10a** in 51% yield. The introduction of a nitro group at C6 deactivated the diene system in the 2-pyridone ring; thus, only traces of desired product **10b** were detected on LCMS even after heating the reaction mixture for 16 h. We further challenged our methodology by employing amino substituted thiazolo-2-pyridone **9c** as a substrate in the [4 + 2] cycloaddition reaction with aryne. We anticipated that both the amino group and the 4π system in thiazolino-2-pyridone **9c** would compete for their reaction with the aryne. Indeed, both components reacted with aryne, resulting in the thiazolo-fused bridged isoquinolone **10c** in 56% yield.

**Scheme 3:**
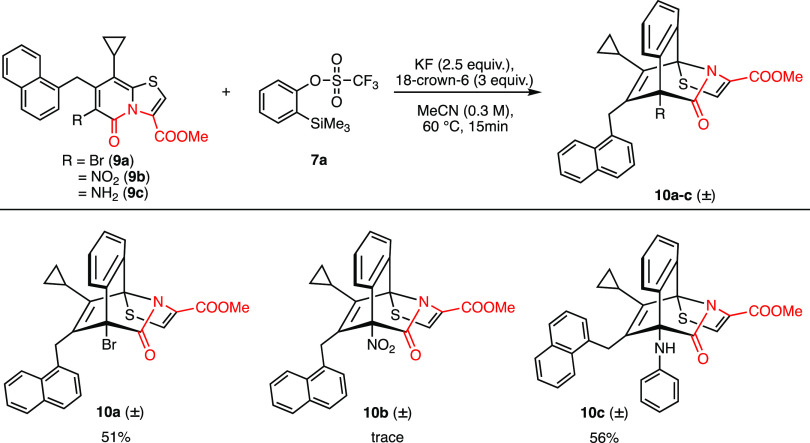
Reaction of C6 Substituted 2-Pyridones **9a**–**c** with In Situ Generated Aryne

Next, we wanted to demonstrate the [4 + 2] cycloaddition reaction on a substrate which is amenable to transformations at the late stage to prepare more structurally diverse complex molecules. Thus, we chose thiazolo-2-pyridone **11** substituted with a methylene chloride group at position C7 for a [4 + 2] cycloaddition reaction with aryne. We envisaged that the resulting product **12**, equipped with an allyl halide type functionality, can be used for late-stage transformations. To our delight, thiazolo-2-pyridone **11** reacted smoothly with an aryne at a small scale (0.4 mmol) to afford **12** in 77% yield (Scheme 4). Further, to demonstrate the scalability of the transformation and to have enough material for the late-stage transformations, the same reaction was performed on gram scale (3.35 mmol) to produce **12** in 77% yield. The methylene chloride functionality could be oxidized to an aldehyde group **13** in 86% yield by refluxing **12** in THF for 4 h in the presence of *N*-methylmorpholine-*N*-oxide (NMO) and KI (Scheme 4). Next, we investigated the reaction of **12** with nucleophiles such as azide and cyanide so that the resulting products could be transformed further. The reaction of **12** with sodium azide at 50 °C for 2 h furnished **14** in 66% either via an S_N_2’ type reaction or Winstein rearrangement.^23^ To get insight into the path of the reaction, **12** was allowed to react with sodium azide at room temperature, that resulted in an inseparable mixture of S_N_2 adduct and **14**. When the isolated mixture was heated at 50 °C in DMF for 2h, the S_N_2 adduct rearranged to **14**, thus confirming the initial S_N_2 reaction followed by Winstein rearrangement. The reaction of **12** with KCN at room temperature after 16 h exclusively resulted in the α,β-unsaturated nitrile **15** in 63% yield via S_N_2 substitution reaction followed by a 1,3-hydrogen shift.

**Scheme 4:**
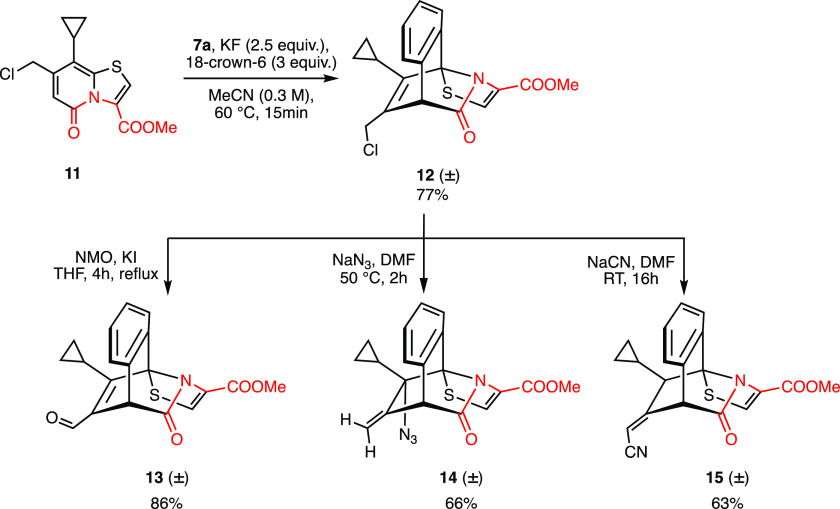
Reaction of C7 Chloromethylene Substituted C2–C3 Oxidized Ring Fused 2-Pyridone **11** with In Situ Generated Aryne (**7a**) in Gram Scale and Late-Stage Functionalization Using a Methylene Chloride Group

In conclusion, we have demonstrated that the oxidation of the C2–C3 bond of ring fused thiazolino-2-pyridones reduces the nucleophilicity of sulfide, thus resulting in a selective [4 + 2] cycloaddition reaction between the diene system in the 2-pyridone ring and the aryne. The developed methodology is scalable and efficiently generates a structurally complex sp^3^ rich peptidomimetic scaffold in high yields. In addition, unsymmetrical 3-methoxy aryne reacted regioselectively with the 2-pyridone ring, which further demonstrates the synthetic utility of the developed methodology. The new ring system obtained is hardly accessible by other synthetic routes and constitutes a new peptidomimetic scaffold that is amenable to late-stage functionalization.

## Experimental Section

### General Details

Chemicals and solvents were purchased from commercial suppliers and used without further purification. Anhydrous solvents (THF, DMF, and MeCN) were dried in a solvent drying system and collected prior to the reaction. Anhydrous reactions were carried out in oven-dried glassware under a nitrogen atmosphere. Reactions were monitored by silica gel TLC plates (median pore size 60 Å) and detected with UV light at 254 nm. Flash column chromatography was performed using an automated chromatography system on silica gel with average particle diameter 50 μm (range 40–65 μm, pore diameter 53 Å) and detection at 254 nm and eluents are given with respective compounds. High resolution mass spectra (HRMS) data were performed using a mass spectrometer micrOTOF II (Bruker) with ESI-TOF (ESI+); calibrated with sodium formate or an Agilent 1290 binary LC system connected to an Agilent 6230 Accurate-Mass TOF LC/MS (ESI+); calibrated with an Agilent G1969-85001 ES-TOF reference mix containing ammonium trifluoroacetate, purine, and hexakis- (1H,1H,3H-tetrafluoropropoxy)phosphazine in 90:10 acetonitrile/water. ^1^H and ^13^C{^1^H} NMR spectra were recorded on a Bruker Advance III 400 MHz or a Bruker Avance III HD 600 MHz spectrometer at 298 K unless otherwise stated and calibrated using the residual peak of the solvent as the internal standard (CDCl_3_, δ_H_ = 7.26 ppm, δ_C_ = 77.16 ppm).

**Caution!** NaN_3_ used for nucleophilic substitution is fatal if swallowed, in contact with skin, or if inhaled.

**Caution!** NaCN produces toxic gas in contact with acids and has toxicity toward different target organs and is highly toxic by inhalation, ingestion, and skin absorption.

**Caution!** The new heteroaromatic compounds were synthesized in small quantity. Peptidomimetic 2-pyridones are known to have diverse biological activities and thus, should be handled carefully.

#### General Procedure for the Synthesis of C2–C3 Oxidized 2-Pyridones (**6a**–**c**)

A oven-dried round-bottom flask (RBF) was cooled under nitrogen atmosphere. The flask was charged with NaH (20.0 mmol, 2.0 equiv (60% in mineral oil)) at room temperature and washed with heptane for 15 min. The RBF was cooled down to 0 °C in an ice bath, and dry MeCN (30 mL) was added and allowed to cool down for 10 min. The solution of 2-pyridone (10.0 mmol, 1.0 equiv) in dry MeCN (30 mL) was added to the flask dropwise at 0 °C. BrCCl_3_ (10.0 mmol, 1.0 equiv) was added to the reaction mixture dropwise for 10 min and stirred at 0 °C for 45 min, then the ice bath was removed and allowed the reaction mixture to come to room temperature for 45 min. Methanol (15.0 mmol, 1.5 equiv) was added dropwise to the reaction mixture and stirred at room temperature for 16h. Completion of the reaction was monitored by TLC. Reaction was quenched by dropwise addition of 6% KHSO_4_ and extracted with EtOAc. The combined organic layer was washed with H_2_O and brine, dried with Na_2_SO_4_, and concentrated in vacuo. The crude product was purified by flash chromatography.

#### Methyl 8-Cyclopropyl-7-(naphthalen-1-ylmethyl)-5-oxo-5*H*-thiazolo[3,2-*a*]pyridine-3-carboxylate (**6a**)

Prepared by following the general procedure for the synthesis of C2–C3 oxidized 2-pyridones using 2-pyridone **I** (Supporting Information). The product was purified with automated flash column chromatography (50 g cartridge; 20–100% ethyl acetate in heptane). Brown solid, 2.68 g (69%). IR (KBr cm^–1^): ν 1723, 1655, 1565, 1471, 1436, 1251, 781. ^1^H NMR (600 MHz, CDCl_3_) δ 7.89–7.87 (m, 1H), 7.85–7.84 (m, 1H), 7.79 (d, *J* = 8.2 Hz, 1H), 7.49–7.48 (m, 2H), 7.42 (dd, *J* = 8.2, 7.0 Hz, 1H), 7.25–7.24 (m, 1H), 7.10 (s, 1H), 5.96 (s, 1H), 4.55 (s, 2H), 3.94 (s, 3H), 1.83–1.78 (m, 1H), 1.06–1.03 (m, 2H), 0.78–0.75 (m, 2H). ^13^C{^1^H} NMR (151 MHz, CDCl_3_) δ: 161.1, 159.0, 153.8, 147.3, 134.2, 133.9, 131.9, 131.4, 128.8, 127.6, 127.3, 126.2, 125.7, 125.5, 123.7, 114.0, 112.3, 111.7, 53.3, 36.2, 10.9, 7.8 (2C). HRMS (ESI^+^) calcd. for C_23_H_20_NO_3_S^+^ (M+H)^+^: 390.1158, found: 390.1161.

#### Methyl 8-Cyclopropyl-7-methyl-5-oxo-5*H*-thiazolo[3,2-*a*]pyridine-3-carboxylate (**6b**)

Prepared by following the general procedure for the synthesis of C2–C3 oxidized 2-pyridones using 2-pyridone **II** (Supporting Information). The product was purified with automated flash column chromatography (50 g cartridge; 20–100% ethyl acetate in heptane). yellow solid, 1.78 g (65%). IR (KBr cm^–1^): ν 1739, 1656, 1566, 1473, 1428, 1245, 1040, 746. ^1^H NMR (400 MHz, CDCl_3_) δ 7.06 (s, 1H), 6.21 (d, *J* = 1.0 Hz, 1H), 3.95 (s, 3H), 2.37 (d, *J* = 0.9 Hz, 3H), 1.76–1.69 (m, 1H), 1.04–1.00 (m, 2H), 0.65–0.61 (m, 2H). ^13^C{^1^H} NMR (100 MHz, CDCl_3_) δ: 161.2, 158.9, 151.8, 146.9, 131.4, 113.6, 112.7, 111.2, 53.3, 20.1, 10.8, 7.6 (2C). HRMS (ESI^+^) calcd. for C_13_H_14_NO_3_S^+^ (M+H)^+^: 264.0689, found: 264.0672.

#### Methyl 8-Methoxy-7-(naphthalen-1-ylmethyl)-5-oxo-5*H*-thiazolo[3,2-*a*]pyridine-3-carboxylate (**6c**)

Prepared by following the general procedure for the synthesis of C2–C3 oxidized 2-pyridones using 2-pyridone **III** (Supporting Information). The product was purified with automated flash column chromatography (50 g cartridge; 20–100% ethyl acetate in heptane). Brown solid, 2.37 g (63%). IR (KBr cm^–1^): ν 1732, 1663, 1574, 1555, 1476, 1249, 996, 787. ^1^H NMR (400 MHz, CDCl_3_) δ 7.91–7.85 (m, 2H), 7.80 (d, *J* = 8.2 Hz, 1H), 7.50–7.41 (m, 2H), 7.43 (dd, *J* = 8.2, 7.0 Hz, 1H), 7.35 (dd, *J* = 7.0, 1.2 Hz, 1H), 7.12 (s, 1H), 5.94 (s, 1H), 4.40 (s, 2H), 3.94 (s, 3H), 3.79 (s, 3H). ^13^C{^1^H} NMR (100 MHz, CDCl_3_) δ 160.6, 157.9, 147.9, 139.1, 136.1, 133.9, 133.5, 131.8, 131.7, 128.8, 127.9, 127.6, 126.4, 125.8, 125.4, 123.7, 114.1, 111.0, 60.5, 53.4, 32.7. HRMS (ESI^+^) calcd. for C_21_H_18_NO_4_S^+^ (M+H)^+^: 380.0951, found: 380.0954.

#### Methyl 7-(Naphthalen-1-ylmethyl)-5-oxo-8-phenyl-5*H*-thiazolo[3,2-*a*]pyridine-3-carboxylate (**6d**)

Prepared by following the general procedure for the synthesis of C2–C3 oxidized 2-pyridones using 2-pyridone **IV** (Supporting Information). The product was purified with automated flash column chromatography (50 g cartridge; 20–100% ethyl acetate in heptane). Brown solid, 2.84 g (66%). IR (KBr cm^–1^): ν 1741, 1658, 1470, 1440, 1231, 781. ^1^H NMR (400 MHz, CDCl_3_) δ 7.75 (dd, *J* = 7.7, 1.6 Hz, 1H), 7.67 (d, *J* = 8.2 Hz, 1H), 7.56–7.53 (m, 1H), 7.43–7.27 (m, 8H), 7.13 (dd, *J* = 7.0, 1.1 Hz, 1H), 6.94 (s, 1H), 5.96 (s, 1H), 4.05 (s, 2H), 3.87 (s, 3H). ^13^C{^1^H} NMR (100 MHz, CDCl_3_) δ 160.8, 159.0, 151.5, 147.1, 135.7, 134.0, 133.9, 131.7, 131.5, 129.8 (2C), 129.6 (2C), 128.8, 128.7, 127.8, 127.7, 126.1, 125.6, 125.4, 123.7, 115.3, 113.9, 111.4, 53.3, 36.8. HRMS (ESI^+^) calcd. for C_26_H_20_NO_3_S^+^ (M+H)^+^: 426.1158, found: 426.1164.

#### Methyl 2-Bromo-8-cyclopropyl-7-(naphthalen-1-ylmethyl)-5-oxo-5*H*-thiazolo[3,2-*a*]pyridine-3-carboxylate (**6e**)

Prepared according to the published procedure (Supporting Information).

#### Methyl 7-(Benzo[*b*]thiophen-2-ylmethyl)-8-cyclopropyl-5-oxo-5*H*-thiazolo[3,2-*a*]pyridine-3-carboxylate (**6f**)

A 20 mL microwave vial was charged with compound **(11)** (2.0 mmol, 1.0 equiv), 2-benzothiophene boronic acid (4.0 mmol, 2.0 equiv), and KF (3.0 mmol, 1.5 equiv). The mixture was dissolved in dry methanol (10 mL) and degassed with N_2_ (g) for 5 min. Bis(triphenylphosphine)palladium(II) dichloride (0.2 mmol, 0.1 equiv) was introduced into the suspension as a catalyst, and the reaction mixture was degassed again under nitrogen for 5 min. The reaction mixture was irradiated at 120 °C for 12 min under microwave radiation. The obtained reaction mixture was extracted with CH_2_Cl_2_, and washed with water and brine respectively. The organic phase was dried over Na_2_SO_4_ and reconcentrated under vacuum. The crude product was purified by automated flash column chromatography (10 g cartridge; 20–100% ethyl acetate in heptane). Brown solid, 240 mg (90%). IR (KBr cm^–1^): ν 1739, 1700, 1455, 1433, 1381, 1365, 1289, 1267, 1214, 752. ^1^H NMR (400 MHz, CDCl_3_) δ 7.65 (dd, *J* = 7.8, 1.3 Hz, 1H), 7.59–7.52 (m, 1H), 7.28–7.12 (m, 2H), 7.02 (s, 1H), 6.92 (d, *J* = 1.1 Hz, 1H), 6.28 (s, 1H), 4.25 (d, *J* = 1.2 Hz, 2H), 3.89 (s, 3H), 1.64–1.57 (m, 1H), 0.97–0.93 (m, 2H), 0.62–0.58 (m, 2H). ^13^C{^1^H} NMR (100 MHz, CDCl_3_) δ: 161.0, 159.0, 152.2, 147.9, 141.9, 139.8, 139.6, 131.5, 124.3, 123.9, 123.0, 122.4, 122.1, 114.3, 112.3, 111.6, 53.4, 34.2, 10.8, 7.9 (2C). HRMS (ESI^+^) calcd. for C_21_H_18_NO_3_S_2_^+^ (M+H)^+^: 396.0723, found: 396.0735.

#### General Procedure for 4 + 2 Cycloaddition

A flame-dried 5 mL Biotage microwave reaction tube equipped with a magnetic stirrer was charged with C2–C3 oxidized 2-pyridone (0.40 mmol, 1.0 equiv), KF (1.0 mmol, 2.5 equiv), and 18-Crown-6 (1.2 mmol, 3.0 equiv) and dissolved in dry MeCN (1.33 mL, 0.3M). The closed microwave reaction tube was then transferred to a 60 °C oil bath and stirred for 10 min to obtain a homogeneous mixture. Substituted or unsubstituted 2-(trimethylsilyl)phenyl trifluoromethanesulfonate (0.80 mmol, 2.0 equiv) was added to the reaction mixture with a syringe, and the mixture was stirred for 15 min. The reaction mixture was then quenched with EtOAc (10 mL) at RT and transferred to a separation funnel with H_2_O (20 mL). The aqueous layer was extracted twice with EtOAc (10 mL). The organic phases were combined, washed with brine (20 mL), dried with Na_2_SO_4_, and concentrated in vacuo. The crude product was purified with flash column chromatography.

#### Methyl (6*S*,10*bS*)-11-Cyclopropyl-12-(naphthalen-1-ylmethyl)-5-oxo-5,6-dihydro-6,10*b*-ethenothiazolo[2,3-*a*]isoquinoline-3-carboxylate (**8a**)

The compound was prepared by following the general procedure of cycloaddition. The crude product was purified by automated flash column chromatography (10 g cartridge; 5–90% ethyl acetate in heptane). White fluffy solid. yield 130 mg (70%). IR (KBr cm^–1^): ν 1739, 1699, 1455, 1434, 1383, 1289, 1127. ^1^H NMR (400 MHz, CDCl_3_) δ 7.98–7.96 (m, 1H), 7.89–7.87 (m, 1H), 7.74 (d, *J* = 8.2 Hz, 1H), 7.54–7.50 (m, 3H), 7.29–7.27 (m, 1H), 7.21 (dd, *J* = 8.2, 7.1 Hz, 1H), 7.15–7.05 (m, 2H), 6.43 (dd, *J* = 7.0, 1.2 Hz, 1H), 6.29 (s, 1H), 4.38–4.23 (m, 3H), 3.76 (s, 3H), 1.80–1.79 (m, 1H), 0.85–0.73 (m, 2H), 0.45–0.42 (m, 1H), 0.01 – −0.02 (m, 1H). ^13^C{^1^H} NMR (100 MHz, CDCl_3_) δ: 164.9, 159.9, 146.9, 144.2, 140.2, 137.5, 133.8, 133.3, 132.1, 128.8, 127.4, 127.0, 126.6 (2C), 126.3, 125.8, 125.7, 125.3, 124.3, 123.2, 121.0, 112.9, 84.7, 59.8, 52.5, 33.5, 11.0, 6.9, 4.5. HRMS (ESI^+^) calcd. for C_29_H_24_NO_3_S^+^ (M+H)^+^: 466.1471, found: 466.1478.

#### Methyl (6*R*,10*bS*)-11-Cyclopropyl-12-methyl-5-oxo-5,6-dihydro-6,10b-ethenothiazolo[2,3-*a*]isoquinoline-3-carboxylate (**8b**)

The compound was prepared by following the general procedure of cycloaddition. The crude product was purified by automated flash column chromatography (10 g cartridge; 5–90% ethyl acetate in heptane). White fluffy solid. yield 90 mg (66%). IR (KBr cm^–1^): ν 1738, 1698, 1657, 1580, 1454, 1434, 1384, 1290, 1266, 1221, 1121, 773, 740. ^1^H NMR (400 MHz, CDCl_3_) δ 7.56–7.54 (m, 1H), 7.47 (dd, *J* = 7.4, 1.1 Hz, 1H), 7.35 (td, *J* = 7.5, 1.2 Hz, 1H), 7.26 (td, *J* = 7.4, 1.2 Hz, 1H), 6.37 (s, 1H), 4.46 (s, 1H), 3.89 (s, 3H), 2.15 (d, *J* = 2.0 Hz, 3H), 1.78–1.73 (m, 1H), 1.05–0.98 (m, 1H), 0.83–0.76 (m, 1H), 0.53–0.47 (m, 1H), 0.02 – −0.03 (m, 1H). ^13^C{^1^H} NMR (100 MHz, CDCl_3_) δ: 165.0, 159.9, 144.5, 143.8, 139.0, 136.9, 126.8, 126.5, 126.5, 124.1, 121.0, 113.1, 84.6, 61.5, 52.5, 16.9, 10.7, 7.6, 4.5. HRMS (ESI^+^) calcd. for C_19_H_17_NNaO_3_S^+^ (M+Na)^+^: 362.0821, found: 362.0856.

#### Methyl (6*S*,10*bR*)-11-Methoxy-12-(naphthalen-1-ylmethyl)-5-oxo-5,6-dihydro-6,10*b*-ethenothiazolo[2,3-*a*]isoquinoline-3-carboxylate (**8c**)

The compound was prepared by following the general procedure of cycloaddition using 2-pyridone **6c** and aryne **7a**. The crude product was purified by automated flash column chromatography (10 g cartridge; 5–90% ethyl acetate in heptane). Dark yellow solid. yield 132 mg (72%). IR (KBr cm^–1^): ν 1740, 1702, 1455, 1434, 1276, 1225, 1126. ^1^H NMR (400 MHz, CDCl_3_) δ 7.86–7.84 (m, 1H), 7.79–7.76 (m, 2H), 7.47–7.33 (m, 4H), 7.20 (td, *J* = 7.6, 1.1 Hz, 1H), 7.04–6.97 (m, 2H), 6.82–6.80 (m, 1H), 6.31 (s, 1H), 4.34 (d, *J* = 16.3 Hz, 1H), 4.18 (s, 1H), 4.06 (d, *J* = 16.3 Hz, 1H), 3.82 (s, 3H), 3.79 (s, 3H). ^13^C{^1^H} NMR (100 MHz, CDCl_3_) δ 165.5, 160.1, 159.5, 143.0, 138.3, 133.7, 133.0, 132.0, 128.6, 127.6, 127.0, 126.6, 126.6, 126.4, 126.1, 125.7, 125.3, 124.2, 123.2, 121.9, 121.2, 112.7, 82.4, 61.8, 58.0, 52.5, 31.6. HRMS (ESI^+^) calcd. for C_27_H_22_NO_4_S^+^ (M+H)^+^: 456.1264, found: 456.1271.

#### Methyl (6*R*,10*bR*)-12-(naphthalen-1-ylmethyl)-5-oxo-11-phenyl-5,6-dihydro-6,10*b*-ethenothiazolo[2,3-*a*]isoquinoline-3-carboxylate (**8d**)

The compound was prepared following the general procedure of cycloaddition using 2-pyridone **6d** and aryne **7a**. The crude product was purified by automated flash column chromatography (10 g cartridge; 5–90% ethyl acetate in heptane). Pale yellow solid. yield 160 mg (80%). IR (KBr cm^–1^): ν 1740, 1701, 1643, 1455, 1434, 1377, 1285, 1226, 1121, 792, 778, 747. ^1^H NMR (400 MHz, CDCl_3_) δ 7.68–7.62 (m, 2H), 7.41 (dd, *J* = 7.5, 1.0 Hz, 1H), 7.31–7.22 (m, 6H), 7.12 (s, 1H), 7.09–6.99 (m, 4H), 6.78 (td, *J* = 7.5, 1.2 Hz, 1H), 6.52 (dd, *J* = 7.3, 1.0 Hz, 1H), 6.01 (s, 1H), 4.31 (s, 1H), 4.04–3.91 (m, 2H), 3.65 (s, 3H). ^13^C{^1^H} NMR (100 MHz, CDCl_3_) δ 165.4, 159.7, 146.9, 143.6, 141.7, 137.8, 134.7, 133.7, 132.9, 132.0, 128.6, 128.5, 128.5 (2C), 128.4, 128.4, 127.7, 127.1, 126.9, 126.5, 126.4, 125.8, 125.7, 125.4, 124.3, 123.4, 121.3, 112.2, 84.2, 59.2, 52.6, 34.6. HRMS (ESI^+^) calcd. for C_32_H_24_NO_3_S^+^ (M+H)^+^: 502.1471, found: 502.1478.

#### Methyl (6*R*,10*bS*)-2-Bromo-11-cyclopropyl-12-(naphthalen-1-ylmethyl)-5-oxo-5,6-dihydro-6,10*b*-ethenothiazolo[2,3-*a*]isoquinoline-3-carboxylate (**8e**)

The compound was prepared by following the general procedure of cycloaddition using 2-pyridone **6e** and aryne **7a**. The crude product was purified by automated flash column chromatography (10 g cartridge; 5–90% ethyl acetate in heptane). White fluffy solid. yield 130 mg (60%). IR (KBr cm^–1^): ν 1738, 1697, 1456, 1435, 1384, 1367, 1293, 1268, 1254, 1216, 1150, 1028, 769. ^1^H NMR (400 MHz, CDCl_3_) δ 8.00–7.97 (m, 1H), 7.93–7.91 (m, 1H), 7.78 (d, *J* = 8.2 Hz, 1H), 7.60–7.54 (m, 3H), 7.34 (td, *J* = 7.5, 1.2 Hz, 1H), 7.26–7.18 (m, 2H), 7.12 (dd, *J* = 7.4, 1.2 Hz, 1H), 6.45 (dd, *J* = 7.0, 1.3 Hz, 1H), 4.39–4.25 (m, 3H), 3.86 (s, 3H), 1.89–1.87 (m, 1H), 0.92–0.77 (m, 2H), 0.50–0.46 (m, 1H), 0.01 – −0.02 (m, 1H). ^13^C{^1^H} NMR (100 MHz, CDCl_3_) δ: 164.4, 160.2, 146.3, 143.5, 140.4, 137.3, 133.8, 133.1, 132.1, 128.8, 127.5, 127.3, 126.9, 126.3, 125.9, 125.7, 125.2, 125.2, 124.4, 123.2, 121.0, 97.5, 84.7, 58.7, 53.1, 33.5, 11.0, 7.0, 4.3. HRMS (ESI^+^) calcd. for C_29_H_22_BrNNaO_3_S^+^ (M+Na)^+^: 566.0396, found: 566.0413.

#### Methyl (6*R*,10b*S*)-12-(Benzo[*b*]thiophen-2-ylmethyl)-11-cyclopropyl-5-oxo-5,6-dihydro-6,10*b*-ethenothiazolo[2,3-*a*]isoquinoline-3-carboxylate (**8f**)

The compound was prepared following the general procedure of cycloaddition using 2-pyridone **6f** and aryne **7a**. The crude product was purified by automated flash column chromatography (10 g cartridge; 5–90% ethyl acetate in heptane). White fluffy solid. yield 100 mg (53%). IR (KBr cm^–1^): ν 1737, 1699, 1587, 1455, 1434, 1382, 1290, 1263, 1219, 1123, 747. ^1^H NMR (400 MHz, CDCl_3_) δ 7.76–7.74 (m, 1H), 7.64–7.62 (m, 1H), 7.52 (dd, *J* = 7.4, 1.1 Hz, 1H), 7.37–7.27 (m, 3H), 7.19–7.12 (m, 2H), 6.69 (s, 1H), 6.32 (s, 1H), 4.50 (s, 1H), 4.31–3.94 (m, 2H), 3.81 (s, 3H), 1.84–1.76 (m, 1H), 0.99–0.95 (m, 1H), 0.83–0.78 (m, 1H), 0.62–0.58 (m, 1H), 0.02 – −0.01 (m, 1H). ^13^C{^1^H} NMR (100 MHz, CDCl_3_) δ: 164.8, 159.8, 147.0, 144.1, 140.6, 139.8, 139.7, 139.2, 137.3, 127.0, 126.6, 126.6, 124.4, 124.3, 123.9, 123.0, 122.3, 122.1, 121.0, 113.0, 84.7, 59.3, 52.5, 31.7, 10.9, 7.5, 4.4. HRMS (ESI^+^) calcd. for C_27_H_21_NNaO_3_S_2_^+^ (M+Na)^+^: 494.0855, found: 494.0874.

#### Methyl (6*R*,10*bS*)-11-cyclopropyl-7-methoxy-12-(naphthalen-1-ylmethyl)-5-oxo-5,6-dihydro-6,10*b*-ethenothiazolo[2,3-*a*]isoquinoline-3-carboxylate (**8g**)

The compound was prepared by following the general procedure of cycloaddition using 2-pyridone **6a** and aryne **7b**. The crude product was purified by automated flash column chromatography (10 g cartridge; 5–90% ethyl acetate in heptane). Orange solid. yield 108 mg (55%). IR (KBr cm^–1^): ν 1737, 1715, 1698, 1588, 1481, 1436, 1382, 1369, 1267, 1232, 795, 783. ^1^H NMR (400 MHz, CDCl_3_) δ 7.82–7.80 (m, 1H), 7.74–7.72 (m, 1H), 7.58 (d, *J* = 8.2 Hz, 1H), 7.39–7.36 (m, 2H), 7.02 (dd, *J* = 8.2, 7.1 Hz, 1H), 6.93 (dd, *J* = 8.4, 7.3 Hz, 1H), 6.67 (dd, *J* = 8.4, 0.9 Hz, 1H), 6.57 (dd, *J* = 7.3, 0.8 Hz, 1H), 6.31 (s, 1H), 6.13 (dd, *J* = 7.1, 1.1 Hz, 1H), 4.21–4.02 (m, 3H), 3.78 (s, 3H), 3.65 (s, 3H), 1.65–1.62 (m, 1H), 0.73–0.67 (m, 2H), 0.26–0.23 (m, 1H), 0.01 – −0.02 (m, 1H). ^13^C{^1^H} NMR (100 MHz, CDCl_3_) δ 165.4, 160.6, 153.0, 150.5, 140.8, 138.8, 133.7, 133.4, 132.1, 130.7, 128.7, 127.7, 127.2, 126.2, 125.8, 125.6, 125.5, 125.2, 123.2, 117.5, 115.9, 111.2, 82.8, 59.9, 56.1, 52.4, 33.2, 11.0, 7.0, 5.2. HRMS (ESI^+^) calcd. for C_30_H_26_NO_4_S^+^ (M+H)^+^: 496.1577, found: 496.1577.

#### Methyl (6*R*,10*bR*)-7,11-Dimethoxy-12-(naphthalen-1-ylmethyl)-5-oxo-5,6-dihydro-6,10*b*-ethenothiazolo[2,3-*a*]isoquinoline-3-carboxylate (**8h**)

The compound was prepared following the general procedure of cycloaddition using 2-pyridone **6c** and aryne **7b**. The crude product was purified by automated flash column chromatography (10 g cartridge; 5–90% ethyl acetate in heptane). Dark orange semisolid. yield 60 mg (31%). IR (KBr cm^–1^): ν 1721, 1589, 1480, 1364, 1271, 1224, 795, 781. ^1^H NMR (400 MHz, CDCl_3_) δ 7.74–7.63 (m, 3H), 7.36–7.32 (m, 1H), 7.29–7.19 (m, 2H) 6.86–6.79 (m, 2H), 6.61 (dd, *J* = 8.5, 0.9 Hz, 1H), 6.35–6.33 (m, 2H), 4.17 (d, *J* = 16.3 Hz, 1H), 4.02 (s, 1H), 3.89 (d, *J* = 16.3 Hz, 1H), 3.76 (s, 3H), 3.70 (s, 3H), 3.68 (s, 3H). ^13^C{^1^H} NMR (100 MHz, CDCl_3_) δ: 166.2, 162.5, 160.3, 153.2, 141.4, 133.6, 133.1, 132.0, 129.4, 128.5, 127.9, 127.5, 126.6, 126.0, 125.9, 125.7, 125.3, 123.2, 121.6, 117.3, 115.7, 111.0, 80.5, 62.1, 57.4, 56.0, 52.4, 31.4. HRMS (ESI^+^) calcd. for C_28_H_24_NO_5_S^+^ (M+H)^+^: 486.1370, found: 486.1378.

#### Methyl (6*R*,10*bR*)-7-Methoxy-12-(naphthalen-1-ylmethyl)-5-oxo-11-phenyl-5,6-dihydro-6,10*b*-ethenothiazolo[2,3-*a*]isoquinoline-3-carboxylate (**8i**)

The compound was prepared by following the general procedure of cycloaddition using 2-pyridone **6d** and aryne **7b**. The crude product was purified by automated flash column chromatography (10 g cartridge; 5–90% ethyl acetate in heptane). Dark yellow solid. yield 131 mg (62%). IR (KBr cm^–1^): ν 1738, 1704, 1590, 1480, 1436, 1375, 1268, 1223, 792. ^1^H NMR (400 MHz, CDCl_3_) δ 7.69–7.67 (m, 1H), 7.63 (d, *J* = 8.2 Hz, 1H), 7.34–7.22 (m, 7H), 7.16 (s, 1H), 7.09–7.05 (m, 1H), 6.92 (dd, *J* = 7.1, 1.1 Hz, 1H), 6.74 (dd, *J* = 8.4, 7.2 Hz, 1H), 6.63 (dd, *J* = 8.5, 0.9 Hz, 1H), 6.18 (d, *J* = 0.8 Hz, 1H), 6.14 (s, 1H), 4.28 (s, 1H), 4.01–3.90 (m, 2H), 3.80 (s, 3H), 3.70 (s, 3H). ^13^C{^1^H} NMR (100 MHz, CDCl_3_) δ 165.8, 160.4, 153.0, 150.0, 140.9, 140.5, 134.6, 133.6, 133.0, 132.0, 130.0, 128.4 (2C), 128.2 (2C), 128.0, 127.6, 127.5 (2C), 127.0, 125.7, 125.6, 125.5, 125.4, 123.3, 117.3, 114.9, 111.3, 82.2, 59.2, 56.3, 52.4, 34.5. HRMS (ESI^+^) calcd. for C_33_H_26_NO_4_S^+^ (M+H)^+^: 532.1577, found: 532.1587.

#### Methyl 6-Bromo-8-cyclopropyl-7-(naphthalen-1-ylmethyl)-5-oxo-5*H*-thiazolo[3,2-*a*]pyridine-3-carboxylate (**9a**)

Compound **6a** (1.0 mmol, 1.0 equiv) was dissolved in acetonitrile (10 mL) in a round bottomed flask. N-bromosuccinimide (1.2 mmol, 1.2 equiv) was added portion wise and stirred at room temperature. The reaction was followed with TLC. Upon completion, saturated aqueous sodium thiosulfate solution (15 mL) was added and the resulting mixture was stirred for another 5 min. The mixture was subsequently partitioned between ethyl acetate (30 mL) and brine (20 mL). The organic phase was dried over Na_2_SO_4_ and concentrated under vacuo. The desired product was purified with automated flash column chromatography (10 g cartridge; 20–80% ethyl acetate in heptane). The pure product obtained as yellow solid. yield 370 mg (79%). IR (KBr cm^–1^): ν 1741, 1648, 1456, 1243, 792, 773. ^1^H NMR (400 MHz, CDCl_3_) δ 8.23–8.21 (m, 1H), 7.94–7.92 (m, 1H), 7.76 (d, J = 8.2 Hz, 1H), 7.67–7.56 (m, 2H), 7.33–7.29 (m, 1H), 7.20 (s, 1H), 6.77 (dd, J = 7.2, 1.2 Hz, 1H), 4.95 (s, 2H), 4.06 (s, 3H), 1.71–1.64 (m, 1H), 0.88–0.84 (m, 2H), 0.65–0.61 (m, 2H). ^13^C{^1^H} NMR (100 MHz, CDCl_3_) δ 160.8, 155.5, 151.7, 146.5, 133.8, 132.8, 132.1, 131.8, 129.0, 127.1, 126.3, 125.8, 125.6, 124.0, 122.9, 114.7, 113.4, 111.0, 53.7, 36.7, 12.2, 7.7 (2C). HRMS (ESI^+^) calcd. for C_23_H_18_BrNNaO_3_S^+^ (M+Na)^+^: 490.0083, found: 490.0088.

#### Methyl 8-Cyclopropyl-7-(naphthalen-1-ylmethyl)-6-nitro-5-oxo-5*H*-thiazolo[3,2-*a*]pyridine-3-carboxylate (**9b**)

To a mixture of Compound **6a** (2.0 mmol, 1.0 equiv) and NaNO_2_ (2.1 mmol, 1.05 equiv) was added 20 mL CH_2_Cl_2_. A balloon filled with oxygen gas was connected to the flask via a rubber septum, and TFA (34.0 mmol, 17.0 equiv) was added dropwise at room temperature. After 6 h the dark red solution was neutralized with NaHCO_3_(aq) and then extracted with CH_2_Cl_2_ (3 × 30 mL). The combined organic phase was washed with brine, dried over Na_2_SO_4_ and concentrated under vacuo. The crude residue was purified by automated flash column chromatography (25 g cartridge; 20–80% ethyl acetate in heptane). The pure product was obtained as a dark green solid. yield 610 mg (68%). IR (KBr cm^–1^): ν 1743, 1666, 1525, 1476, 1352, 1246, 1174, 795, 778. ^1^H NMR (600 MHz, CDCl_3_) δ 8.10–8.09 (m, 1H), 7.87 (dd, *J* = 8.1, 1.4 Hz, 1H), 7.73 (d, *J* = 8.2 Hz, 1H), 7.60–7.51 (m, 2H), 7.38 (s, 1H), 7.32 (dd, *J* = 8.2, 7.1 Hz, 1H), 6.93 (dd, *J* = 7.1, 1.3 Hz, 1H), 4.75 (s, 2H), 4.01 (s, 3H), 1.49–1.44 (m, 1H), 0.83–0.80 (m, 2H), 0.62–0.60 (m, 2H). ^13^C{^1^H} NMR (151 MHz, CDCl_3_) δ: 160.0, 151.6, 151.1, 145.3, 136.5, 133.6, 132.6, 132.6, 131.5, 128.9, 127.5, 126.5, 125.9, 125.4, 124.8, 122.7, 116.6, 111.6, 53.7, 31.4, 11.3, 7.8 (2C). HRMS (ESI^+^) calcd. for C_23_H_19_N_2_O_5_S^+^ (M+H)^+^: 435.1009, found: 435.1016.

#### Methyl 6-Amino-8-cyclopropyl-7-(naphthalen-1-ylmethyl)-5-oxo-5*H*-thiazolo[3,2-*a*]pyridine-3-carboxylate (**9c**)

6-Nitropyridone (**9b**) (0.55 mmol, 1.0 equiv) was dissolved in 5 mL of acetic acid in a RBF. Freshly activated zinc powder (5.5 mmol, 10 equiv) was added to the resulting mixture portion wise and stirred vigorously for 16h at room temperature. Upon completion, the reaction mixture was neutralized with saturated NaHCO_3_, diluted with DCM (30 mL) and filtered through the cotton wool. The aqueous layer was extracted with DCM (2 × 20 mL). The combined organic layer was washed with H2O (20 mL) and brine (20 mL), dried over Na_2_SO_4_, and concentrated in vacuo. The crude residue was purified by with automated flash column chromatography (10 g cartridge; 20–80% ethyl acetate in heptane). The pure product obtained as dark brown solid. yield 175 mg (78%). IR (KBr cm^–1^): ν 3439, 3357, 1737, 1640, 1573, 1493, 1371, 1248, 1215, 793, 775. ^1^H NMR (400 MHz, CDCl_3_) δ 8.25 (dd, *J* = 8.3, 1.1 Hz, 1H), 7.95–7.93 (m, 1H), 7.78 (d, *J* = 8.2 Hz, 1H), 7.67–7.57 (m, 2H), 7.33–7.30 (m, 1H), 7.15 (s, 1H), 6.88 (q, *J* = 1.3 Hz, 1H), 4.68 (s, 2H), 4.05 (s, 3H), 3.86 (s, 2H), 1.49–1.44 (m, 1H), 0.89–0.85 (m, 2H), 0.64–0.60 (m, 2H). ^13^C{^1^H} NMR (100 MHz, CDCl_3_) δ 161.3, 154.2, 133.9, 133.4, 132.1, 132.1, 130.7, 130.4, 128.9, 128.6, 127.4, 126.3, 125.9, 125.7, 123.4, 123.1, 115.3, 113.9, 53.3, 31.0, 11.6, 7.1 (2C). HRMS (ESI^+^) calcd. for C_23_H_21_N_2_O_3_S^+^ (M+H)^+^: 405.1267, found: 405.1272.

#### Methyl (6*S*,10*bS*)-6-Bromo-11-cyclopropyl-12-(naphthalen-1-ylmethyl)-5-oxo-5,6-dihydro-6,10*b*-ethenothiazolo[2,3-*a*]isoquinoline-3-carboxylate (**10a**)

The compound was prepared following the general procedure of cycloaddition using compound **9a** and aryne **7a**. The crude product was purified by automated flash column chromatography (10 g cartridge; 5–80% ethyl acetate in heptane). Pale yellow solid. yield 111 mg (51%). IR (KBr cm^–1^): ν 1742, 1713, 1453, 1378, 1277, 1214, 818. ^1^H NMR (400 MHz, CDCl_3_) δ 8.17–8.14 (m, 1H), 7.95–7.92 (m, 1H), 7.75–7.58 (m, 5H), 7.48–7.33 (m, 2H), 7.12 (dd, *J* = 8.3, 7.2 Hz, 1H), 6.39 (s, 1H), 6.06 (dd, *J* = 7.1, 1.3 Hz, 1H), 4.57–4.46 (m, 2H), 3.88 (s, 3H), 1.94–1.87 (m, 1H), 0.80–0.75 (m, 2H), 0.46–0.44 (m, 1H), 0.01 – −0.01 (m, 1H). ^13^C{^1^H} NMR (100 MHz, CDCl_3_) δ 160.9, 160.0, 147.6, 142.1, 140.3, 137.8, 134.5, 133.6, 131.5, 128.8, 127.9, 127.7, 126.9, 126.8, 126.2, 125.8, 125.1, 125.1, 124.3, 123.0, 121.0, 112.8, 83.1, 74.5, 52.8, 33.9, 12.6, 6.7, 4.5. HRMS (ESI^+^) calcd. for C_29_H_22_BrNNaO_3_S^+^ (M+Na)^+^: 566.0396, found: 566.0413.

#### Methyl (6*S*,10*bS*)-11-Cyclopropyl-12-(naphthalen-1-ylmethyl)-5-oxo-6-(phenylamino)-5,6-dihydro-6,10*b*-ethenothiazolo[2,3-*a*]isoquinoline-3-carboxylate (**10c**)

The compound was prepared by following the general procedure of cycloaddition using compound **9c** and aryne **7a**. The crude product was purified by automated flash column chromatography (10 g cartridge; 5–90% ethyl acetate in heptane). Dark brown solid. yield 125 mg (56%) IR (KBr cm^–1^): ν 3387, 1739, 1703, 1601, 1507, 1385, 1268, 1208, 749, 728. ^1^H NMR (400 MHz, CDCl_3_) δ 7.69 (dd, *J* = 8.2, 1.4 Hz, 1H), 7.57 (d, *J* = 8.2 Hz, 1H), 7.44 (dd, *J* = 7.4, 1.1 Hz, 1H), 7.38 (dd, *J* = 8.5, 1.1 Hz, 1H), 7.31–7.27 (m, 1H), 7.24–7.14 (m, 5H), 7.00–6.94 (m, 4H), 6.65–6.60 (m, 1H), 6.22 (d, *J* = 7.9 Hz, 1H), 6.18 (s, 1H), 5.34 (s, 1H), 4.19–4.01 (m, 2H), 3.66 (s, 3H), 1.44–1.42 (m, 1H), 0.69–0.62 (m, 2H), 0.45–0.42 (m, 1H), 0.02–0.00 (m, 1H). ^13^C{^1^H} NMR (100 MHz, CDCl_3_) δ: 164.0, 159.7, 147.4, 144.2, 144.1, 142.7, 136.5, 134.9, 133.7, 131.6, 128.7, 128.5, 127.1, 126.8, 126.6, 126.1, 125.6 (2C), 125.4, 125.3 (2C), 125.2 (2C), 123.5, 121.1, 117.3, 114.7, 113.7, 83.4, 71.0, 52.5, 30.4, 12.3, 9.2, 5.5. HRMS (ESI^+^) calcd. for C_35_H_28_N_2_NaO_3_S^+^ (M+Na)^+^: 579.1713, found: 579.1732.

#### Methyl 7-(Chloromethyl)-8-cyclopropyl-5-oxo-5*H*-thiazolo[3,2-*a*]pyridine-3-carboxylate (**11**)

*Step 1*. A 100 mL RBF equipped with a magnetic stirrer was charged with compound **V** (Supporting Information) (7.0 mmol, 1.0 equiv) and dissolved in DCM (25 mL). The resulting solution was transferred to a ice bath and cooled to 0 °C. m-CPBA (7.0 mmol, 1.0 equiv) was added portion wise to the resulting reaction. The reaction mixture was stirred for 3 h, and consumption of starting material was confirmed by TLC. The resulting reaction mixture was then quenched by the addition of 15 mL of saturated NaHCO_3_. The resulting solution was extracted with DCM (3 × 40 mL). The combined organic layer was washed with H_2_O and brine, dried over Na_2_SO_4_, and concentrated in vacuo. The resulting sulfoxide product was utilized for the next reaction.

*Step 2*. A flame-dried 100 mL RBF equipped with a magnetic stirrer was charged with sulfoxide intermediate (3.48 mmol, 1.0 equiv), toluene (22 mL), and trifluoroacetic anhydride (17.4 mmol, 5.0 equiv). The resulting mixture was heated to 60 °C for 3 h, then allowed to cool, and concentrated under vacuo. The resulting mixture was diluted in DCM (2 mL), which was added dropwise to precooled (0 °C) concentrated sulfuric acid (98%, 6.58 mL) with stirring. The reaction was carried out for 1 h at RT, cooled down to 0 °C and poured onto ice. The resulting mixture was quenched with NaHCO_3_ and extracted with DCM (3 × 50 mL). The combined organic layer was washed with H_2_O and brine, dried over Na_2_SO_4_, and concentrated in vacuo. The crude product was purified by automated flash column chromatography (50 g cartridge; 20–80% ethyl acetate in heptane). The final compound was obtained as a bright yellow solid (720 mg, 55%). IR (KBr cm^–1^): ν 1737, 1650, 1557, 1474, 1244, 1229, 1041. ^1^H NMR (400 MHz, CDCl_3_) δ: 7.16 (s, 1H), 6.50 (s, 1H), 4.70 (s, 2H), 3.99 (s, 3H), 1.94–1.87 (m, 1H), 1.14–1.10 (m, 2H), 0.78–0.74 (m, 2H). ^13^C{^1^H} NMR (100 MHz, CDCl_3_) δ: 160.8, 158.9, 149.1, 148.2, 131.7, 114.6, 111.6, 111.3, 53.4, 42.5, 10.2, 7.4 (2C). HRMS (ESI^+^) calcd. for C_13_H_13_ClNO_3_S^+^ (M+H)^+^: 298.0299, found: 298.0313.

#### Methyl (6*S*,10*bR*)-12-(Chloromethyl)-11-cyclopropyl-5-oxo-5,6-dihydro-6,10*b*-ethenothiazolo[2,3-*a*]isoquinoline-3-carboxylate (**12**)

The compound was prepared by following the general procedure of cycloaddition using compound **11** and aryne **7a**. The crude product was purified by automated flash column chromatography (10 g cartridge; 5–90% ethyl acetate in heptane). Bright yellow solid. yield 115 mg (77%). This reaction was also performed using **11** (3.35 mmol, 1 g), **7a** (6.7 mmol, 1.63 mL), KF (8.3 mmol, 2.5 equiv), and 18-Crown-6 (10.0 mmol, 3.0 equiv) and dissolved in dry MeCN (11.2 mL, 0.3M). 958 mg (76%) of isolated product were obtained. IR (KBr cm^–1^): ν 1740, 1701, 1585, 1455, 1435, 1381, 1292, 1225, 1127, 1025, 992, 720. ^1^H NMR (400 MHz, CDCl_3_) δ 7.56–7.50 (m, 2H), 7.37–7.33 (m, 1H), 7.28 (td, *J* = 7.5, 1.3 Hz, 1H), 6.34 (s, 1H), 4.74–4.69 (m, 2H), 4.42 (dd, *J* = 11.6, 1.6 Hz, 1H), 3.86 (s, 3H), 1.85–1.77 (m, 1H), 1.09–1.02 (m, 1H), 0.87–0.80 (m, 1H), 0.72–0.67 (m, 1H), 0.02 – −0.04 (m, 1H). ^13^C{^1^H} NMR (100 MHz, CDCl_3_) δ: 164.4, 159.7, 149.2, 143.3, 137.7, 136.9, 127.5, 126.8, 126.5, 124.3, 121.4, 112.9, 84.3, 58.3, 52.6, 41.7, 10.7, 7.0, 4.3. HRMS (ESI^+^) calcd. for C_19_H_17_ClNO_3_S^+^ (M+H)^+^: 374.0612, found: 374.0629.

#### Methyl (6*S*,10*bR*)-11-Cyclopropyl-12-formyl-5-oxo-5,6-dihydro-6,10*b*-ethenothiazolo[2,3-*a*]isoquinoline-3-carboxylate (**13**)

*N*-Methylmorpholine-*N*-oxide (1.60 mmol, 3.0 equiv) and potassium iodide (0.16 mmol, 0.3 equiv) were added portion wise to a solution of compound **12** (0.54 mmol, 1.0 equiv) in dry THF (30 mL) at 0 °C under nitrogen atmosphere. The reaction mixture was stirred for 4h at reflux under nitrogen atmosphere. The reaction mixture was allowed to cool to room temperature and solvent was removed under reduced pressure. The reaction mixture was dissolved in H_2_O (20 mL) and extracted with EtOAc (3 × 10 mL). The combined organic layer was washed with H_2_O and brine, dried over Na_2_SO_4_, and concentrated in vacuo. The crude product was purified by automated flash column chromatography (10 g cartridge; 10–60% ethyl acetate in heptane). Bright yellow solid. Yield 162 mg (86%). IR (KBr cm^–1^): ν 1740, 1704, 1668, 1456, 1434, 1375, 1292, 1274, 1226, 1123, 744. ^1^H NMR (600 MHz, CDCl_3_) δ 10.15 (s, 1H), 7.46 (dd, *J* = 7.5, 1.1 Hz, 1H), 7.35 (dd, *J* = 7.3, 1.1 Hz, 1H), 7.21–7.19 (m, 1H), 7.14 (td, *J* = 7.4, 1.2 Hz, 1H), 6.20 (s, 1H), 5.21 (s, 1H), 3.69 (s, 3H), 1.96–1.91 (m, 1H), 1.20–1.15 (m, 1H), 0.86–0.82 (m, 1H), 0.71–0.66 (m, 1H), 0.02 – −0.03 (m, 1H). ^13^C{^1^H} NMR (151 MHz, CDCl_3_) δ: 185.2, 166.9, 163.5, 159.6, 142.5, 139.2, 136.3, 128.3, 127.1, 126.5, 125.1, 122.5, 112.2, 84.6, 52.7, 51.7, 11.2, 9.1, 4.1. HRMS (ESI^+^) calcd. for C_19_H_15_NNaO_4_S^+^ (M+Na)^+^: 376.0614, found: 376.0631.

#### Methyl (6*S*,10*bR*,11R)-11-azido-11-cyclopropyl-12-methylene-5-oxo-5,6-dihydro-10*b*,6-ethanothiazolo[2,3-*a*]isoquinoline-3-carboxylate (**14**)

In a dry RBF, compound **12** (0.30 mmol, 1.0 equiv) and sodium azide (0.90 mmol, 3.0 equiv) were added and dissolved in 5 mL of DMF. The reaction mixture was stirred for 2 h at 50 °C. Upon completion, the reaction mixture was diluted with EtOAc (20 mL) and extracted once with sat. NaHCO_3_ (10 mL) and once with brine (10 mL). The aqueous phase was re-extracted with EtOAc (2 × 10 mL), dried, and concentrated in vacuo. The crude product was purified by automated flash column chromatography (10 g cartridge; 10–50% ethyl acetate in heptane). Brown fluffy solid. Yield 76 mg (65%). IR (KBr cm^–1^): ν 2110, 1740, 1706, 1458, 1435, 1377, 1290, 1227, 1135, 756, 730. ^1^H NMR (600 MHz, CDCl_3_) δ 7.37–7.35 (m, 1H), 7.20–7.15 (m, 3H), 6.15 (s, 1H), 5.46 (s, 1H), 5.19 (s, 1H), 4.28 (s, 1H), 3.61 (s, 3H), 0.63–0.59 (m, 1H), 0.13 – −0.04 (m, 4H). ^13^C{^1^H} NMR (151 MHz, CDCl_3_) δ: 163.2, 158.9, 138.7, 137.9, 132.5, 129.4, 127.2, 126.8, 124.0, 123.6, 115.5, 114.0, 88.1, 71.7, 58.3, 51.8, 17.1, 0.0, −0.3. HRMS (ESI^+^) calcd. for C_19_H_17_N_4_O_3_S^+^ (M+H)^+^: 381.1016, found: 381.1032.

#### Methyl (6*R*,10*bS*,11*R*,*E*)-12-(Cyanomethylene)-11-cyclopropyl-5-oxo-5,6-dihydro-10*b*,6-ethanothiazolo[2,3-*a*]isoquinoline-3-carboxylate (**15**)

In a dry RBF, compound **10** (0.29 mmol, 1.0 equiv) and sodium cyanide (0.58 mmol, 2.0 equiv) were added and dissolved in 5 mL of DMF. The reaction mixture was stirred for 16 h at room temperature. Upon completion, the reaction mixture was diluted with EtOAc (20 mL) and extracted once with sat. NaHCO_3_ (10 mL) and once with brine (10 mL). The aqueous phase was reextracted with EtOAc (2 × 10 mL), dried, and concentrated in vacuo. The crude product was purified by with automated flash column chromatography (10 g cartridge; 10–50% ethyl acetate in heptane). Dark brown solid. Yield 67 mg (63%). IR (KBr cm^–1^): ν 2220, 1739, 1706, 1457, 1435, 1377, 1293, 1222, 1124, 1025, 760, 733. ^1^H NMR (400 MHz, CDCl_3_) δ 7.72–7.70 (m, 1H), 7.60–7.53 (m, 3H), 6.38 (s, 1H), 5.75 (d, *J* = 2.0 Hz, 1H), 5.21 (s, 1H), 3.91 (s, 3H), 2.82 (dd, *J* = 10.3, 2.0 Hz, 1H), 0.97–0.91 (m, 1H), 0.65–0.60 (m, 2H), 0.49–0.44 (m, 1H), 0.05 – −0.03 (m, 1H). ^13^C{^1^H} NMR (100 MHz, CDCl_3_) δ 162.0, 159.7, 158.7, 138.6, 130.8, 130.3, 128.4, 127.4, 125.2, 124.2, 115.3, 113.8, 96.1, 82.9, 57.6, 57.2, 52.7, 14.7, 3.4, 3.3. HRMS (ESI^+^) calcd. for C_20_H_17_N_2_O_3_S^+^ (M+H)^+^: 365.0954, found: 365.0983.

## Data Availability

The data underlying this study are available in the published article and its Supporting Information.

## Supporting Information Available

Extra information for the synthesis of thiazolino ring fused 2-pyridone peptidomimetics, optimization of the conditions, crystal data and copies of ^1^H and ^13^C{^1^H} NMR spectra for all new compounds (PDF)
