# Supplementary material for: Synthesis of Three-Dimensional Ring Fused Heterocycles by a Selective [4 + 2] Cycloaddition Between Bicyclic Thiazolo 2-Pyridones and Arynes
Source: J Org Chem. 2023 Dec 14;89(1):731–9. doi: 10.1021/acs.joc.3c01957 (PMC10777404; doi:10.1021/acs.joc.3c01957)
Supplement: Supplementary file 1 — jo3c01957_si_001.pdf [file jo3c01957_si_001.pdf]

Synthesis of Three-Dimensional Ring Fused Heterocycles by a Selective [4+2] Cycloaddition Between Bicyclic Thiazolo 2-Pyridones and Arynes

Souvik Sarkar[a], Pardeep Singh[a]*, Simon Edin[b], Ola F. Wendt[b] and Fredrik Almqvist[a]*

[a]Umeå University, Department of Chemistry, 901 87 Umeå, Sweden.

[b]Centre for Analysis and Synthesis, Lund University, SE-221 00 Lund, Sweden

*Corresponding authors. E-mail: fredrik.almqvist@umu.se; pardeep.singh@umu.se

Table of Contents

1. Synthesis of thiazolino ring fused 2-pyridone peptidomimetics ………………………. S2

2. Optimization of the conditions ………... S2

3. Crystal data ..……………………………………………………………………………S2

4. Copies of ^{1}H, ^{13}C{^{1}H} spectra S4

5. References ………………………………………………………………………….S28

1. Synthesis of thiazolino ring fused 2-pyridone peptidomimetics

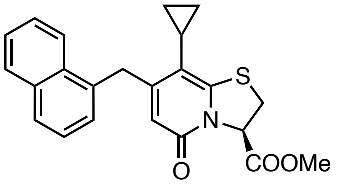

I

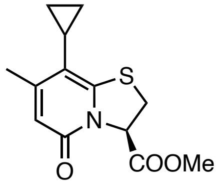

II

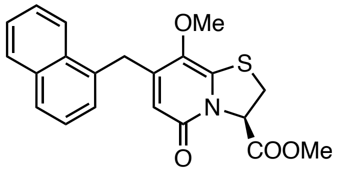

III

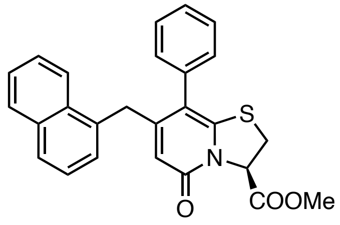

IV

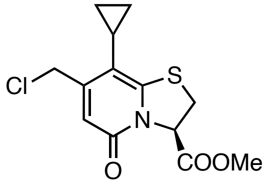

V

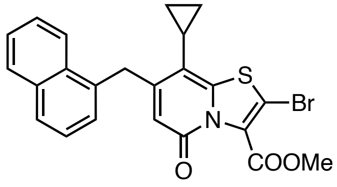

6e

Compound I[1-3], II[3], III[4], IV[5, 6], V[3], and 6e[7] were synthesized according to already published experimental procedures.

2. Optimization of the conditions

Table S1. Optimization of reaction conditions for selective [4+2] cycloaddition.

entry	F^- source	aryne (equivalent)	time	solvent	temp (°C)	yield[b] (%)
1[a]	CsF	1.5	16 h	THF	RT	11
2	TBAT	1.5	16 h	THF	RT	61
3	TBAF	1.5	16 h	THF	RT	6
4	KF, 18-crown-6	1.5	16 h	THF	RT	64
5	KF, 18-crown-6	1.5	16 h	THF	0	56
8	KF, 18-crown-6	1.5	16 h	THF	60	75
9	KF, 18-crown-6	1.5	16 h	MeCN	60	78
10	KF, 18-crown-6	1.5	16 h	Toluene	60	57
11	KF, 18-crown-6	1.5	16 h	dioxane	60	70
12	KF, 18-crown-6	1.5	1 h	MeCN	60	73
13	KF, 18-crown-6	1.5	15 min	MeCN	60	68
14[c]	KF, 18-crown-6	2	15 min	MeCN	60	76
15[c]	KF, 18-crown-6	2	15 min	MeCN	RT	52

[a]Unless otherwise stated, the reactions were carried out with C2-C3 oxidized ring fused 2 pyridone 1a (1.0 equiv), benzyne 2a (1.5 equiv) and fluorine source (2 equiv) (18-Crown-6 (2.5 equiv) when needed with fluorine source) in dry solvent (0.3M) for different time period at the mentioned temperature. [b]Isolated yield. [c]Benzyne (2.0 equiv), KF (2.5 equiv), 18-crown-6 (3 equiv). Abbreviations: TBAT, tetrabutylammonium difluorotriphenylsilicate; TBAF, Tetrabutylammonium fluoride; RT, room temperature.

3. Crystal data. X-ray quality crystals of **8f** were obtained through diffusion crystallization from an ethanol/dichloromethane mixture and diffusing pentane. Intensity data was collected with an Oxford Diffraction Excalibur 3 system, using ω-scans and Mo Kα (λ = 0.71073 Å) radiation.[7] The data was extracted, integrated and empirically absorption corrected using Crysalis RED.[8] The structure was solved by direct methods and refined by full-matrix least-squares calculations on F^2

using SHELXL implemented in Olex 2-1.5.[9] Molecular graphics were generated using Crystal Maker 10.6.2.[10] CCDC deposition number 2113369.

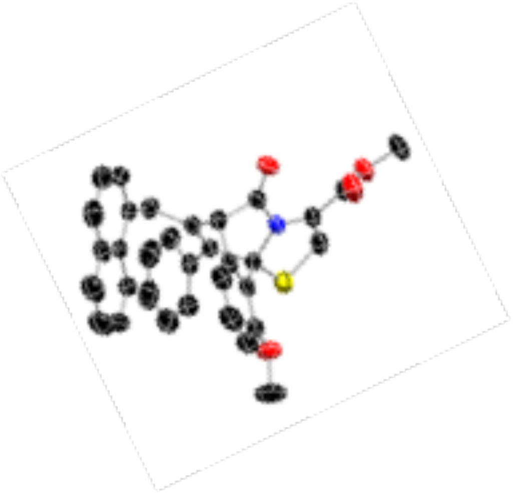

Figure S1. Crystal Structure of **8f**

Table S2. Crystal data and structure refinement details for the structure

	**8f**
**Empirical formula**	C33 H25 N O4 S
**Formula weight**	531.60
**Temperature/K**	293(2)
**Crystal system**	Triclinic
**Space group**	$P\bar{1}$
**a/Å**	8.2153(3)
**b/Å**	13.0979(5)
**c/Å**	13.3653(5)
**β/°**	107.106(4)
**Volume/Å³**	1335.891(9)
**Z**	2
**ρ_{calc}g/cm³**	1.322
**μ/mm⁻¹**	0.161
**F(000)**	556.0
**Θ range for data collection/°**	3.345 to 29.836
**Reflections collected**	29708
**Independent reflections**	6583 [R_{int} = 0.0384, R_{sigma} = 0.0407]
**Data/restraints/parameters**	6583/0/354
**Goodness-of-fit on F²**	1.021
**Final R indexes [I>=2σ (I)]**	R1 = 0.0553<br>wR2 = 0.1096
**Final R indexes [all data]**	R1 = 0.0877<br>wR2 = 0.1237
**Largest diff. peak/hole / e Å⁻³**	0.22 / -0.26
**CCDC**	2113369

4. Copies of ^{1}H, $^{13}C\{^{1}H\}$ spectra

Compound 6a. ^{1}H NMR (600 MHz, $CDCl_3$) $^{13}C\{^{1}H\}$ NMR (151 MHz, $CDCl_3$)

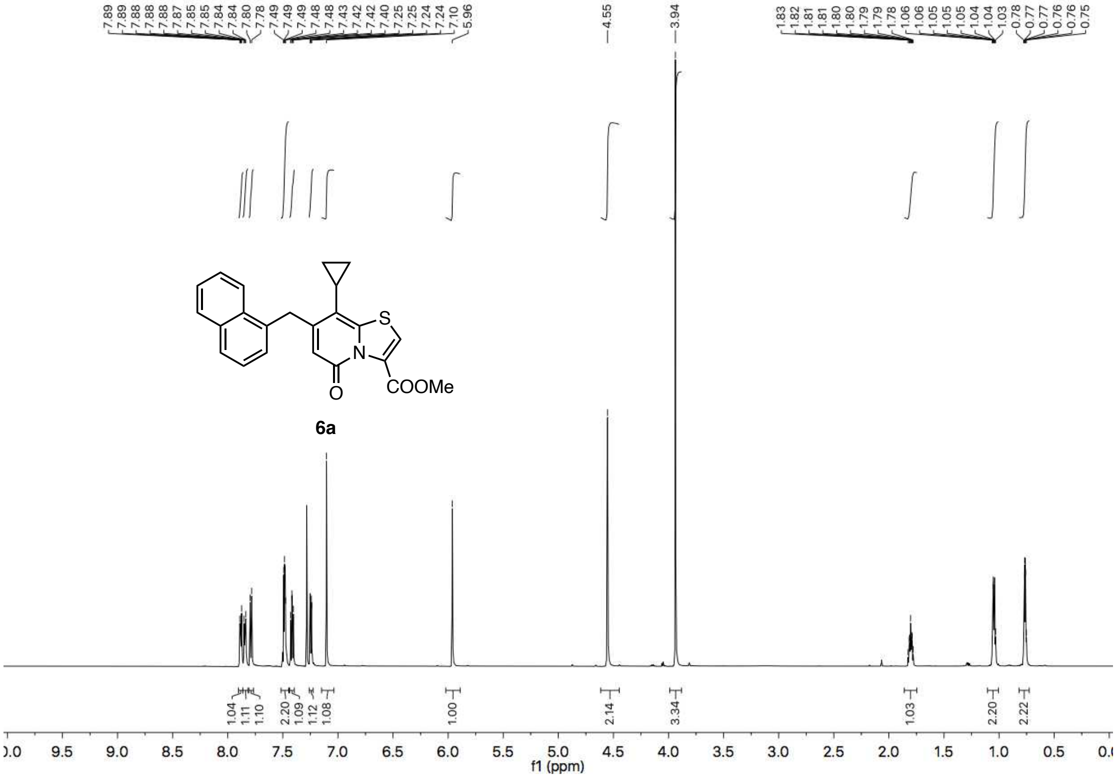

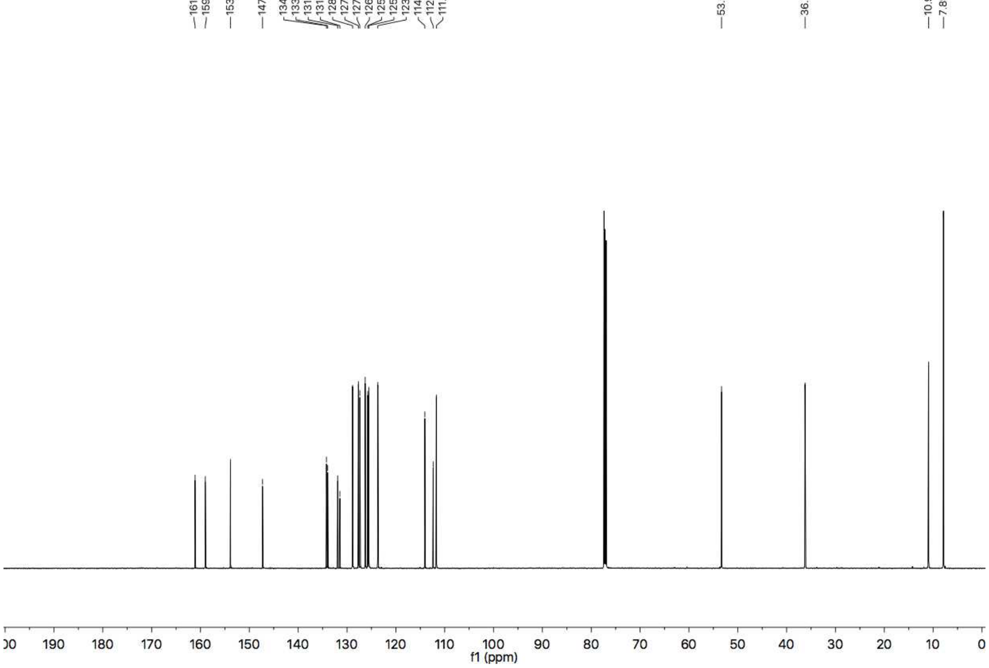

Compound 6b. ^{1}H NMR (400 MHz, $CDCl_3$) $^{13}C\{^{1}H\}$ NMR (100 MHz, $CDCl_3$)

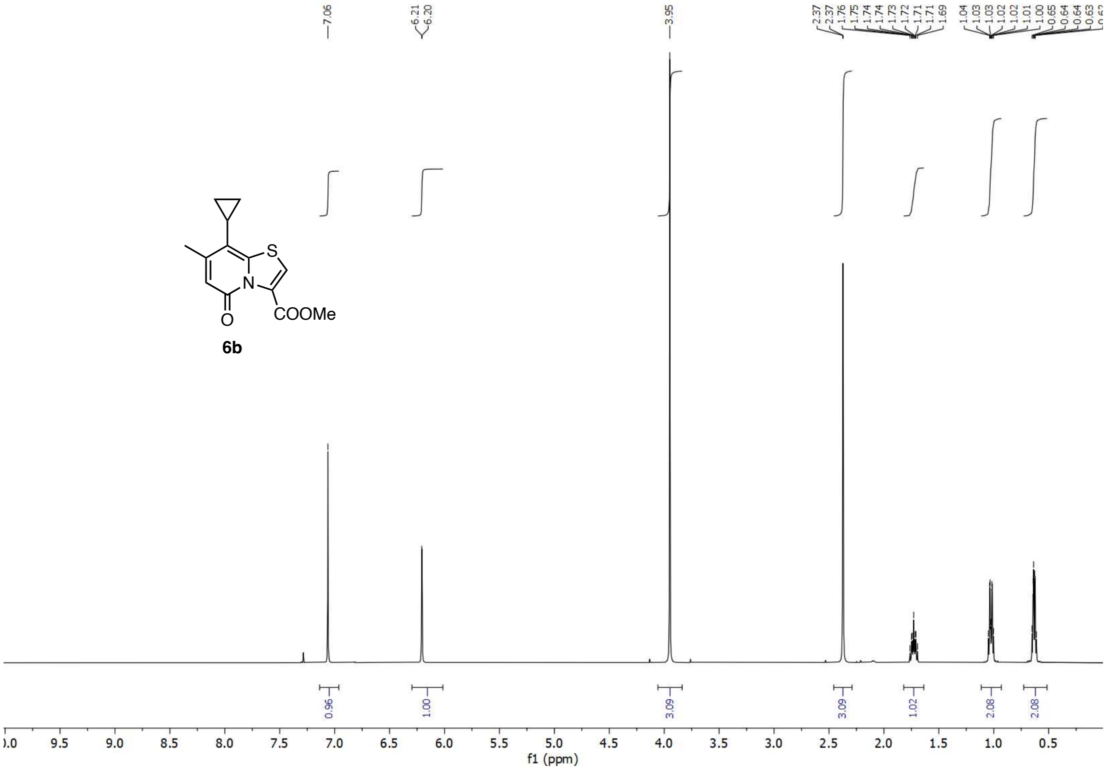

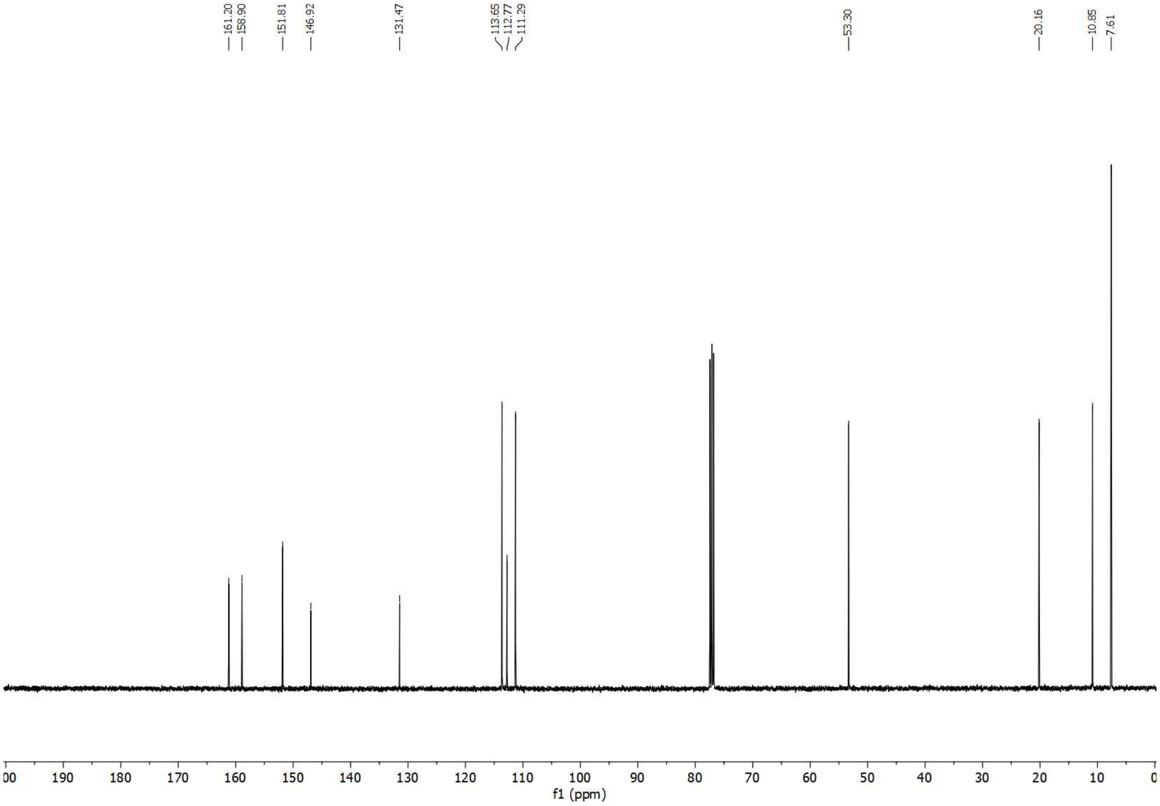

Compound 6c. ^{1}H NMR (400 MHz, $CDCl_3$) $^{13}C\{^{1}H\}$ NMR (100 MHz, $CDCl_3$)

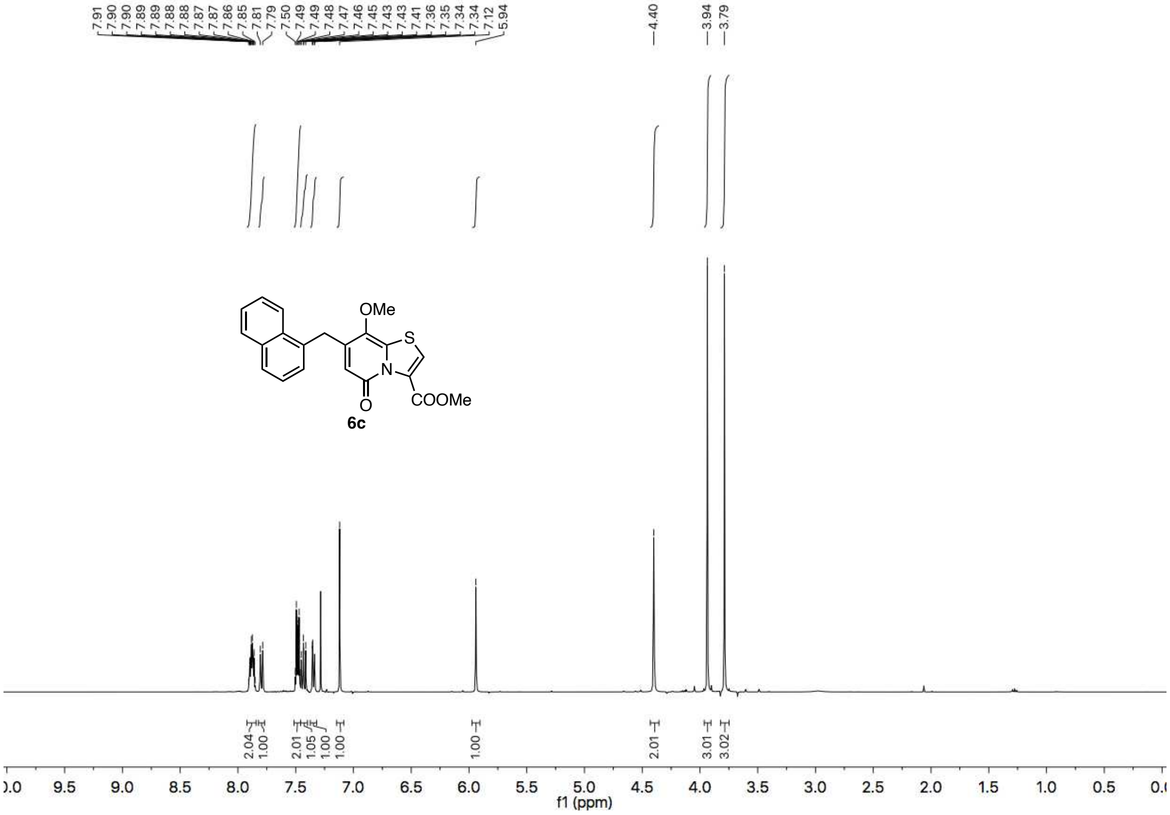

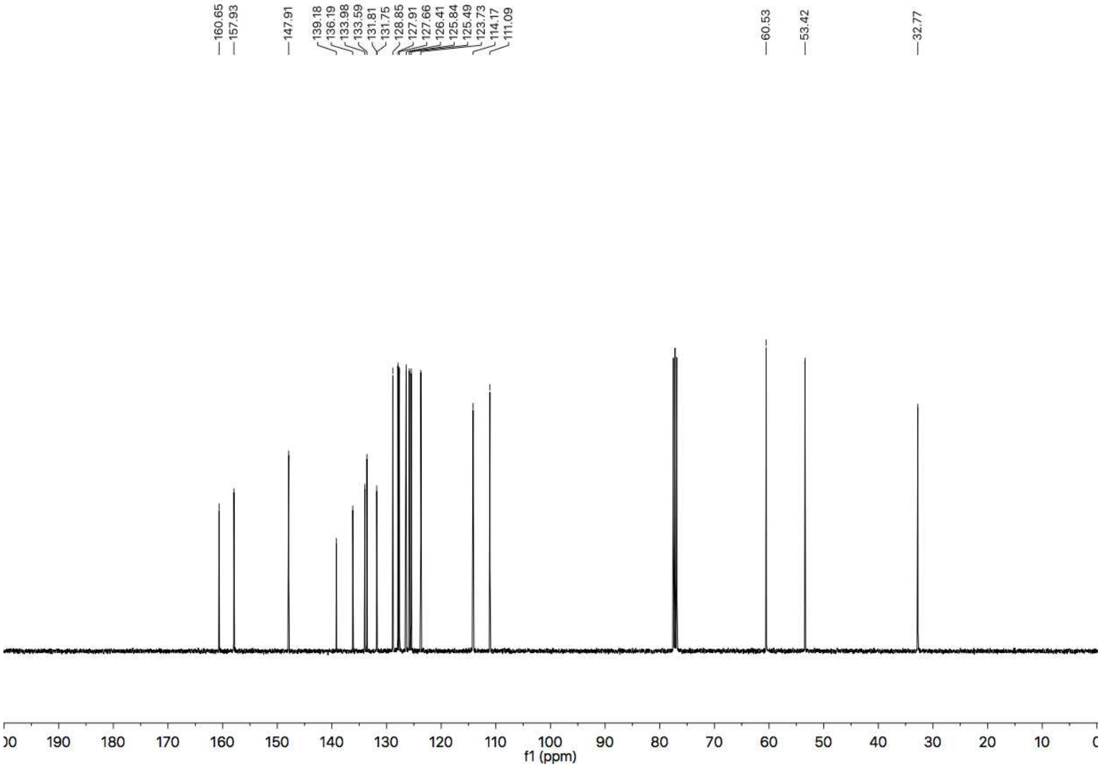

Compound 6d. ^{1}H NMR (400 MHz, $CDCl_3$) ^{13}C{^{1}H} NMR (100 MHz, $CDCl_3$)

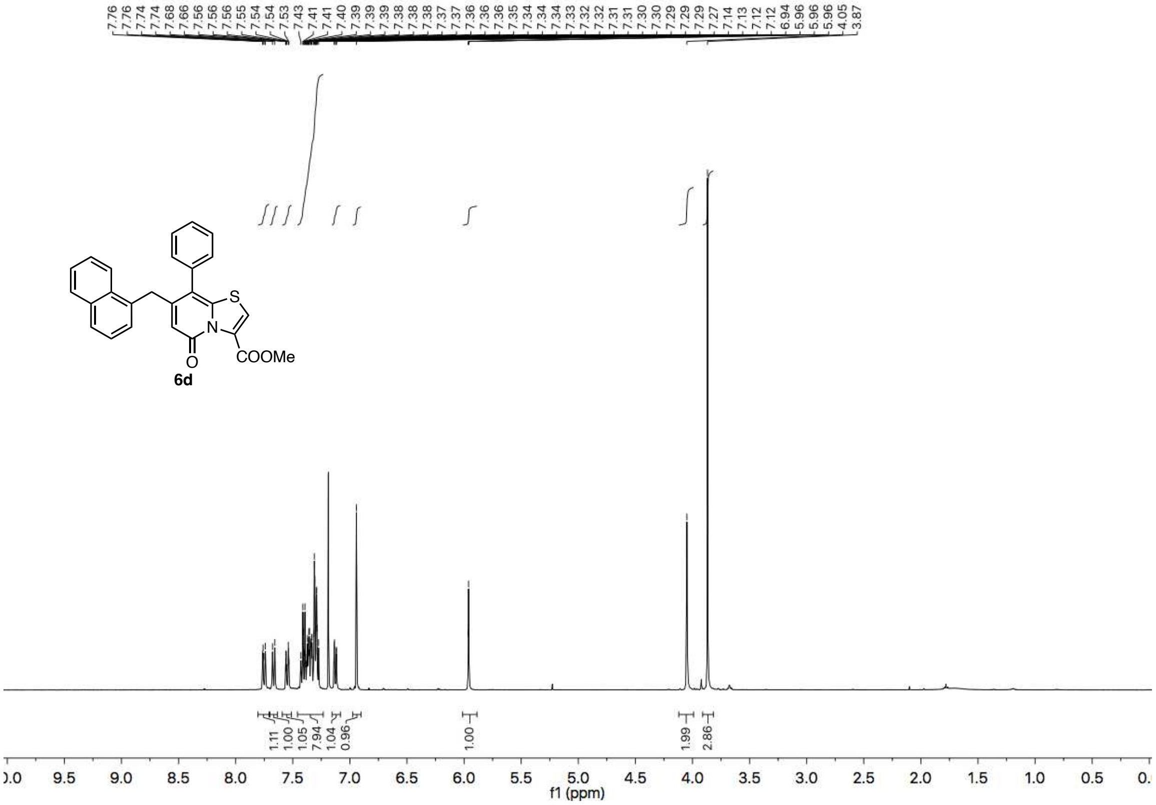

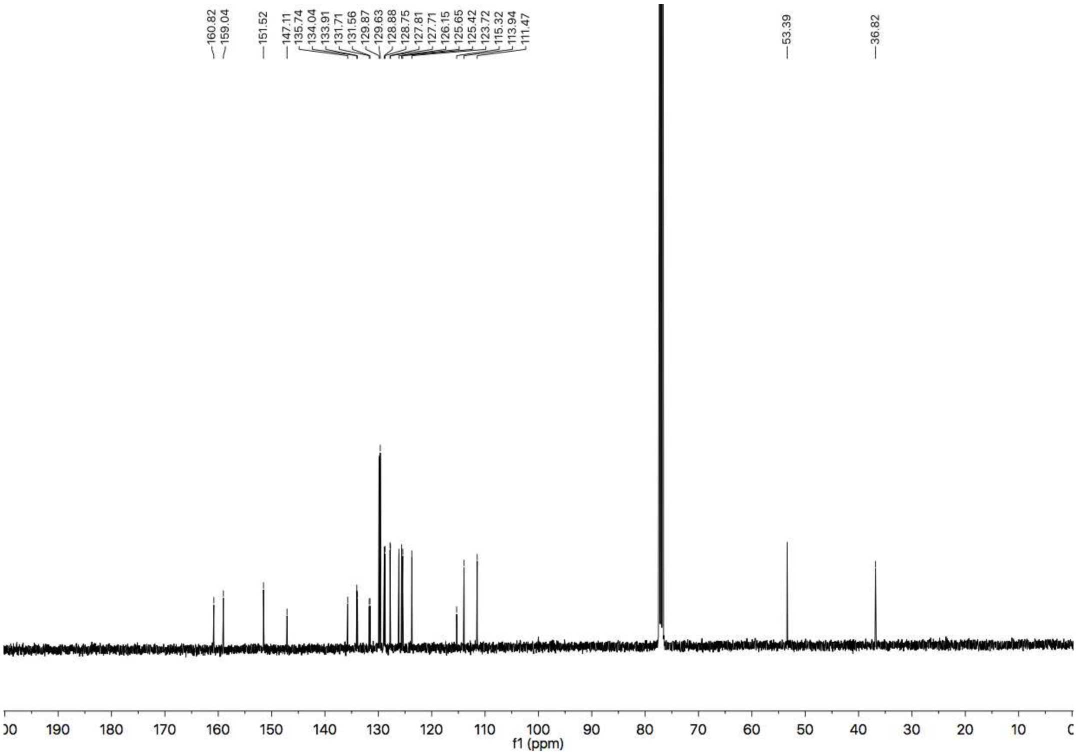

Compound 6f. ^{1}H NMR (400 MHz, CDCl$_3$) ^{13}C{^{1}H} NMR (100 MHz, CDCl$_3$)

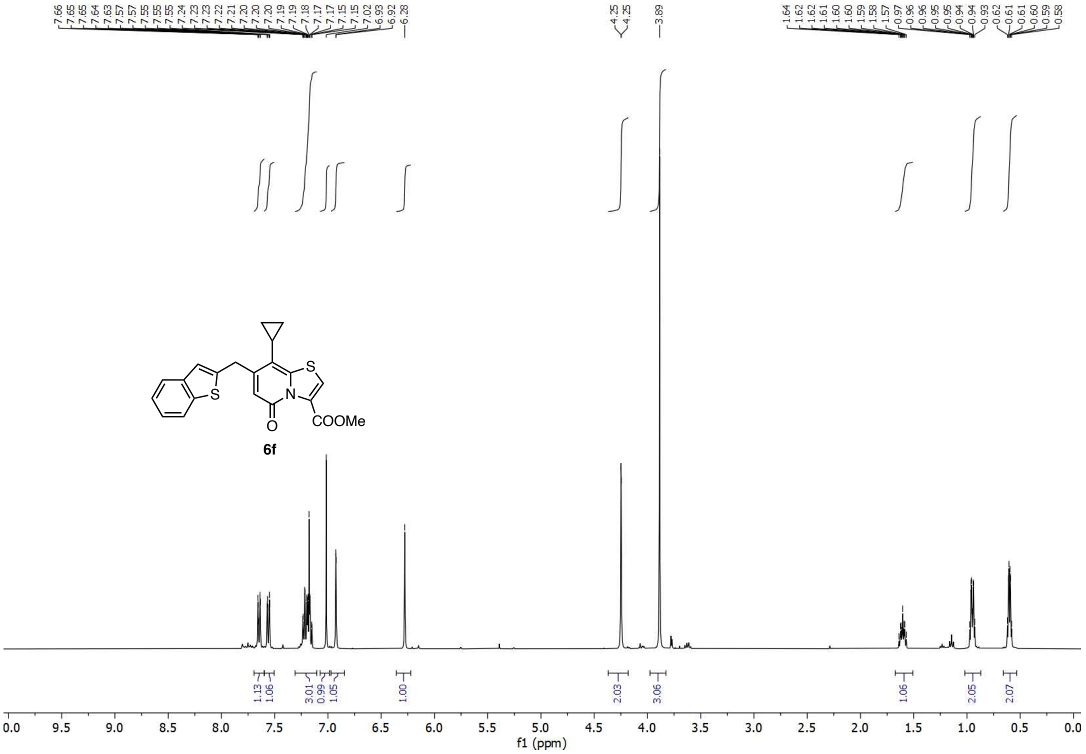

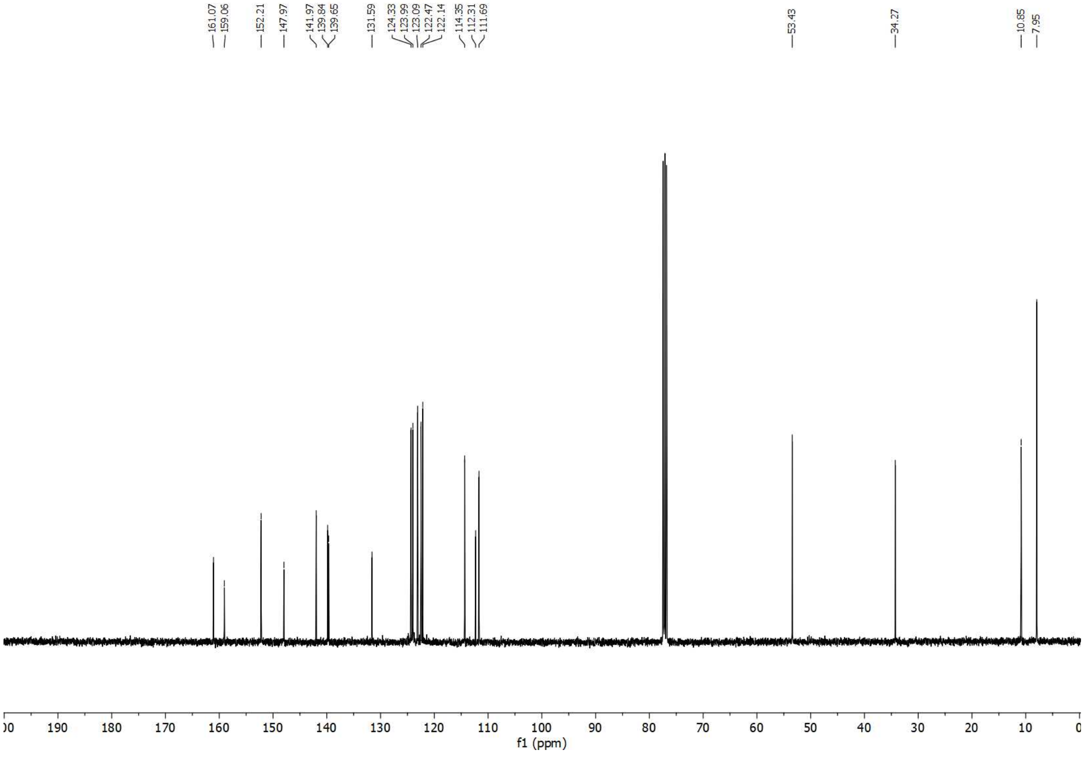

Compound 8a. ^{1}H NMR (400 MHz, $CDCl_3$) ^{13}C{^{1}H} NMR (100 MHz, $CDCl_3$)

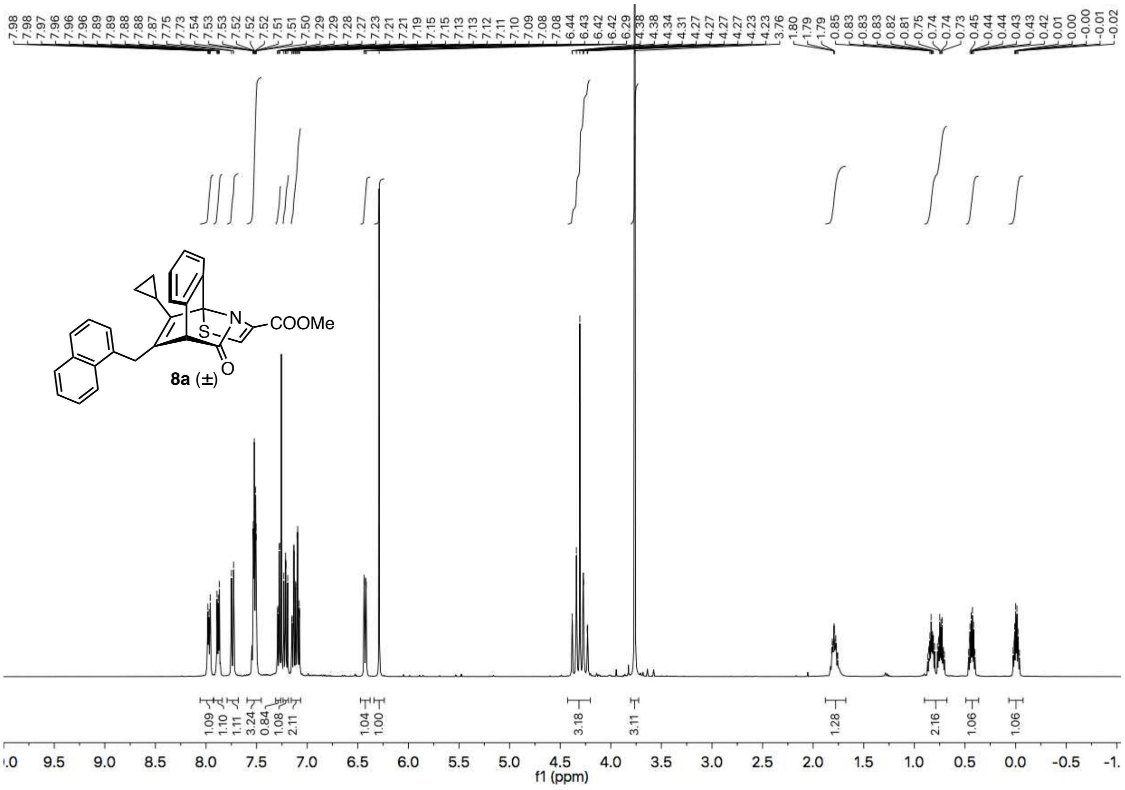

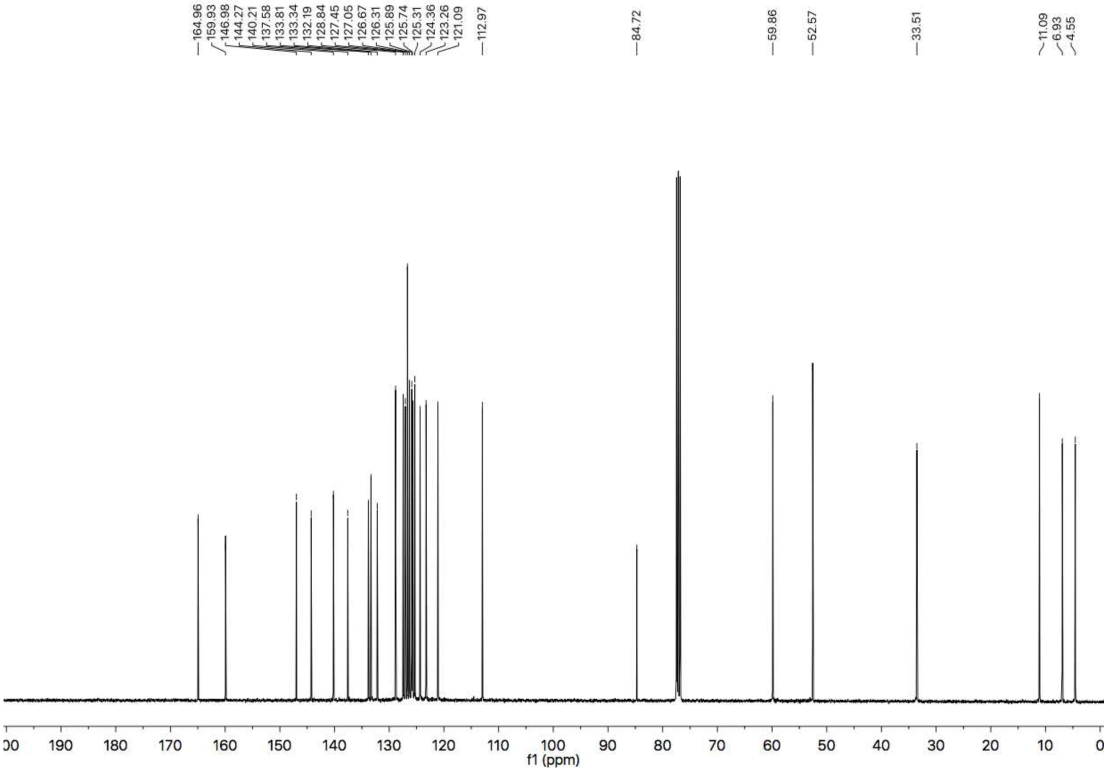

Compound 8b. ^{1}H NMR (400 MHz, $CDCl_3$) $^{13}C\{^{1}H\}$ NMR (100 MHz, $CDCl_3$)

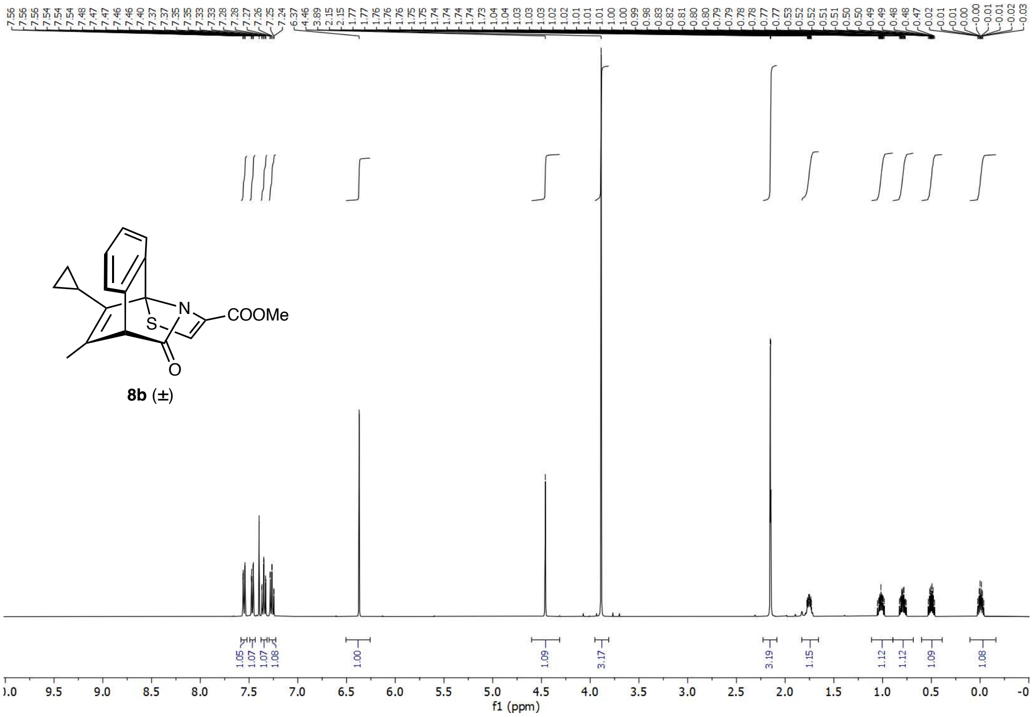

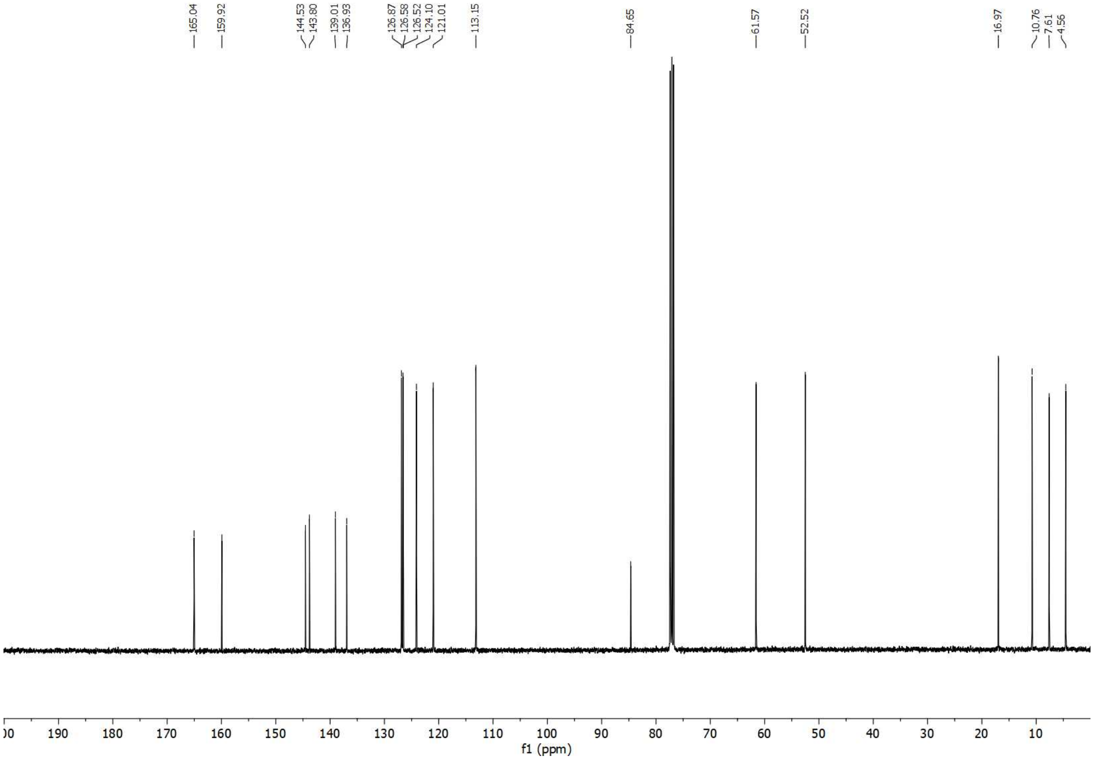

Compound 8c. ^{1}H NMR (400 MHz, $CDCl_3$) $^{13}C\{^{1}H\}$ NMR (100 MHz, $CDCl_3$)

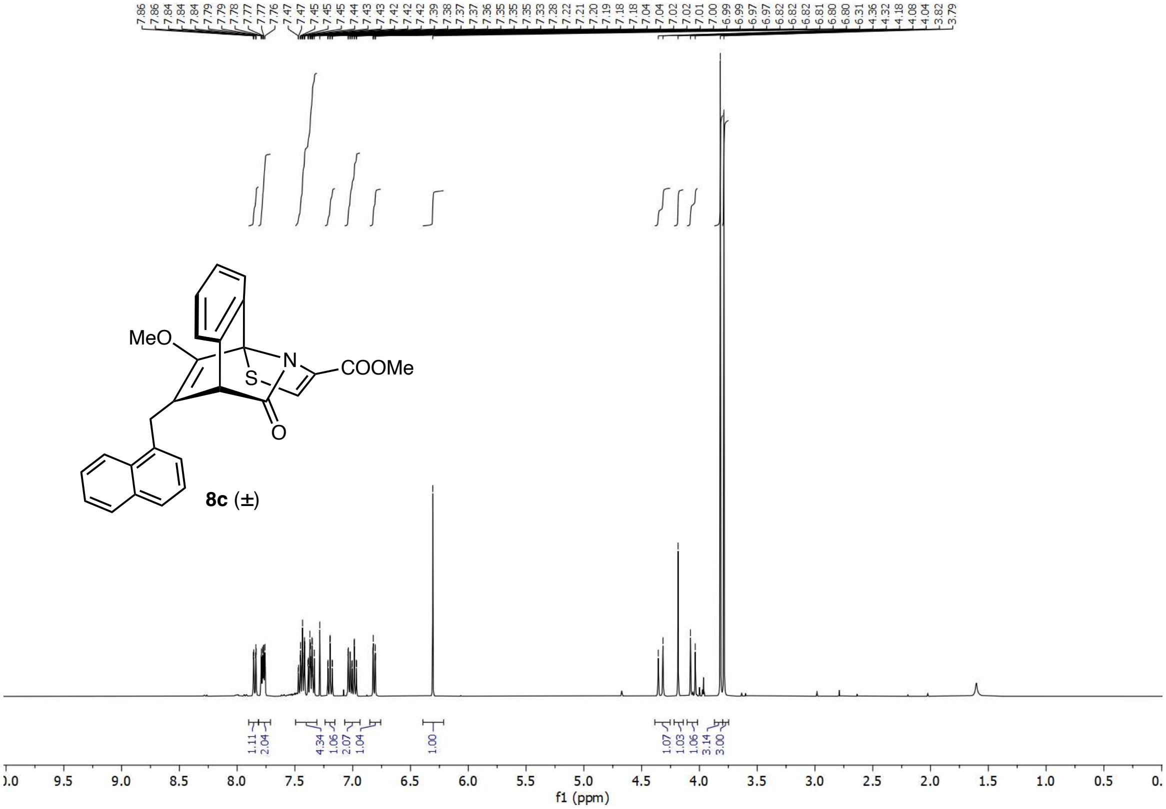

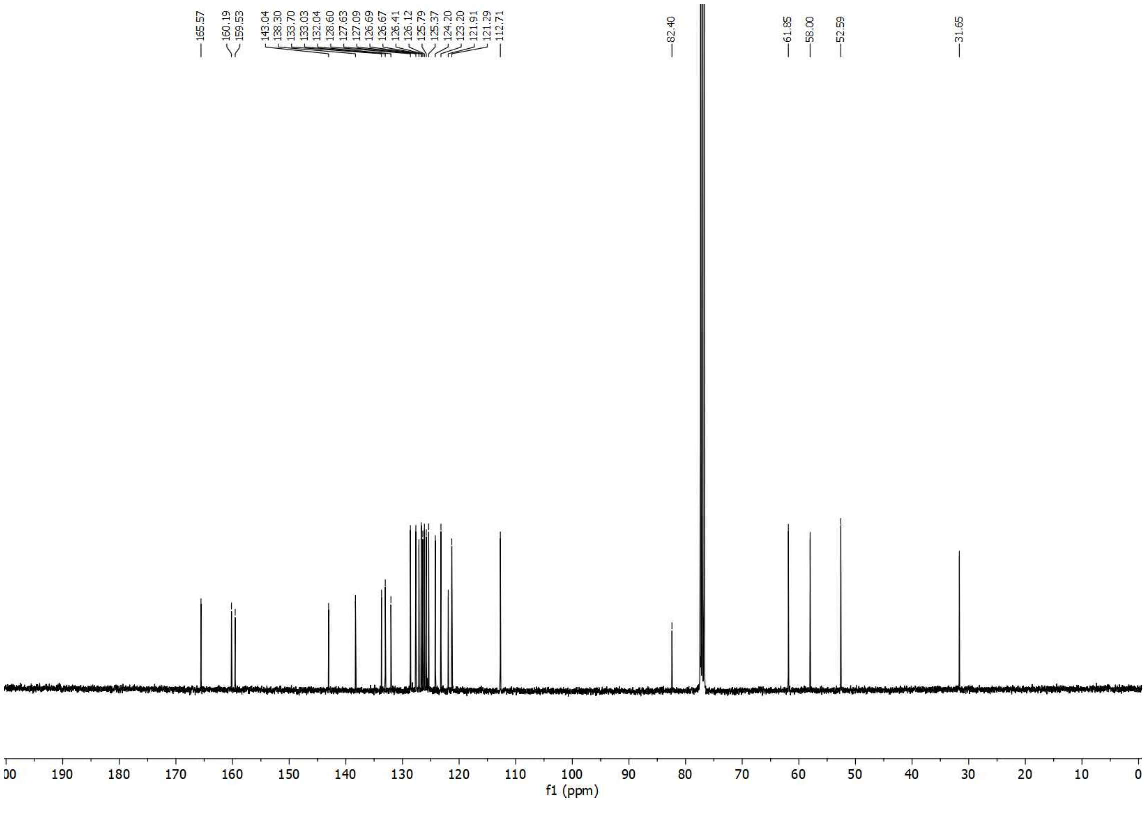

Compound 8d. ^{1}H NMR (400 MHz, $CDCl_3$) $^{13}C\{^{1}H\}$ NMR (100 MHz, $CDCl_3$)

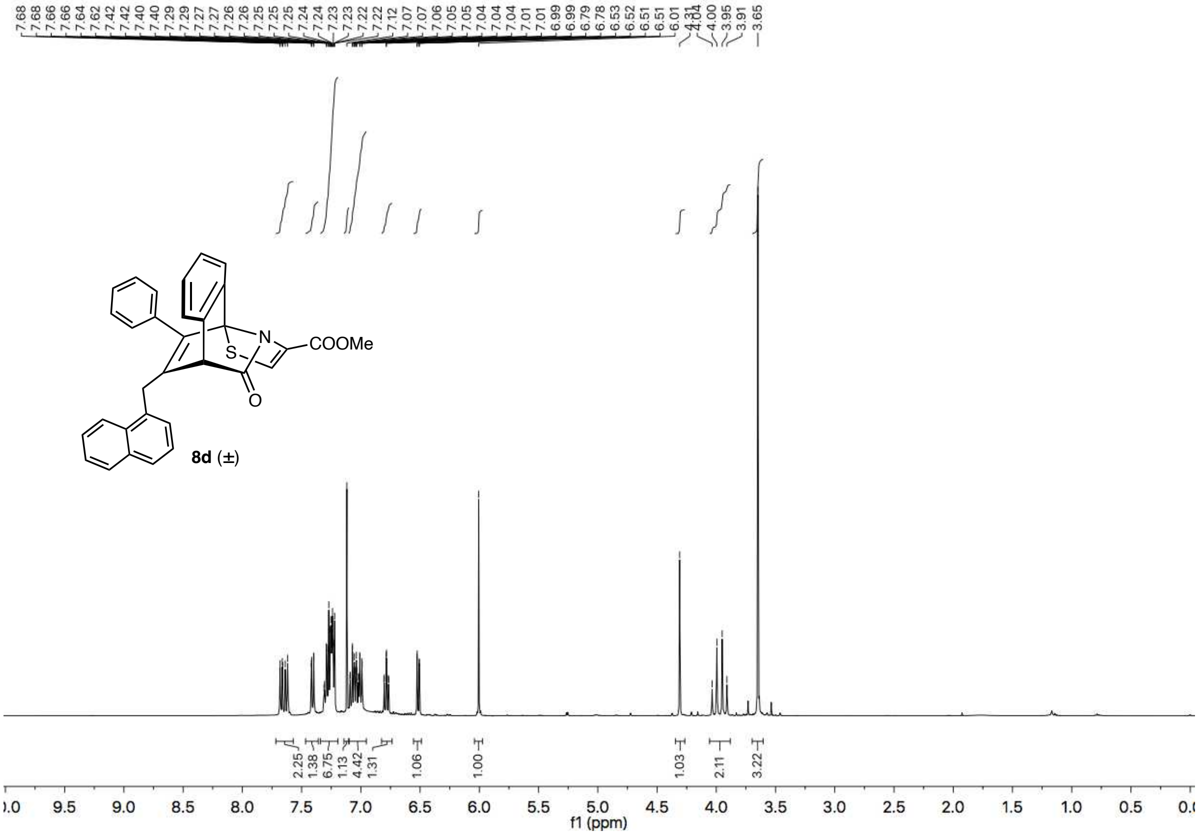

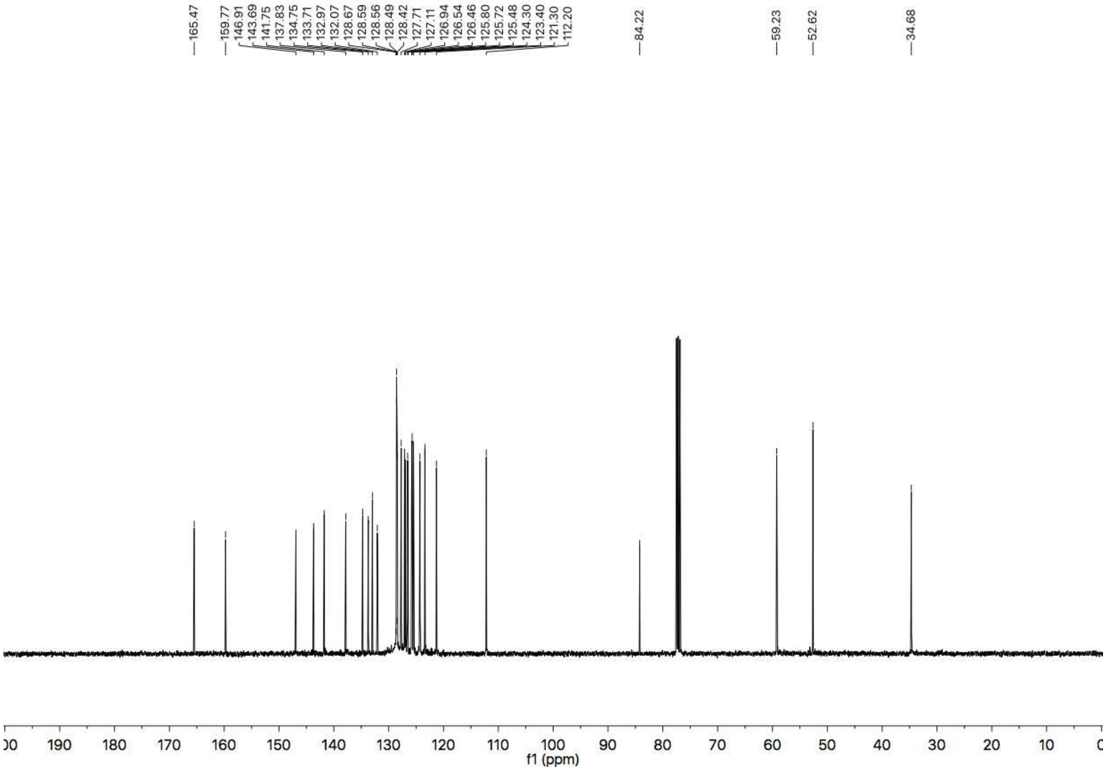

Compound 8e. ^{1}H NMR (400 MHz, $CDCl_3$) $^{13}C\{^{1}H\}$ NMR (100 MHz, $CDCl_3$)

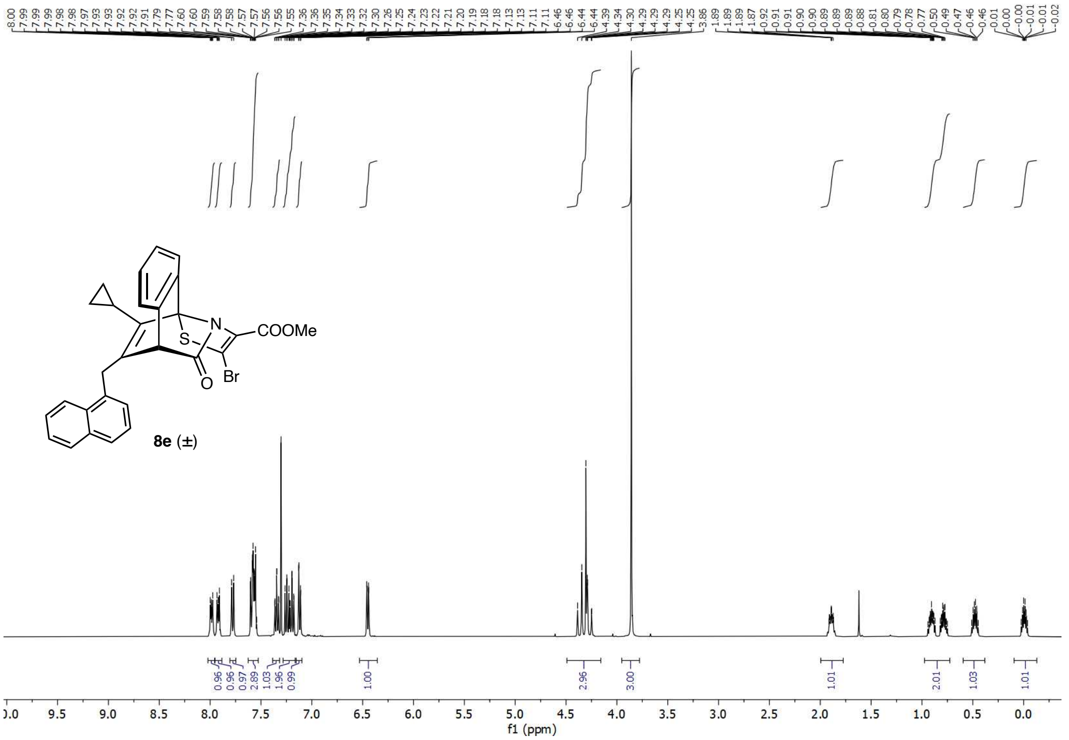

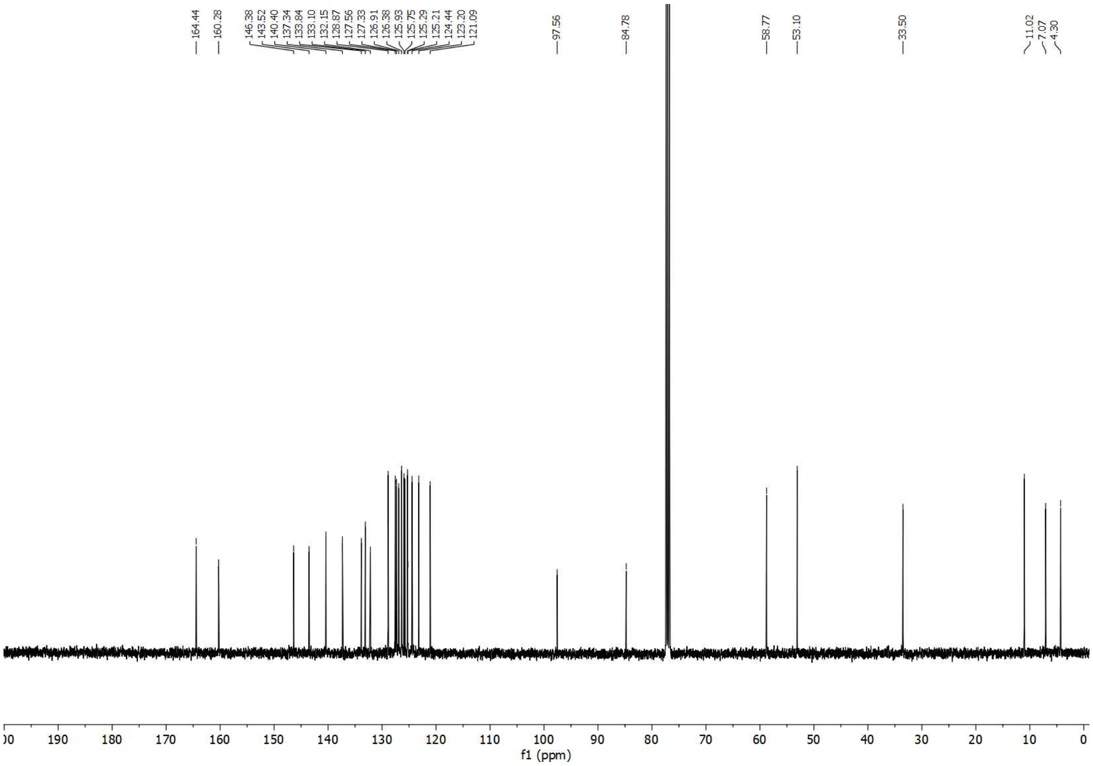

Compound 8f. ^{1}H NMR (400 MHz, $CDCl_3$) $^{13}C\{^{1}H\}$ NMR (100 MHz, $CDCl_3$)

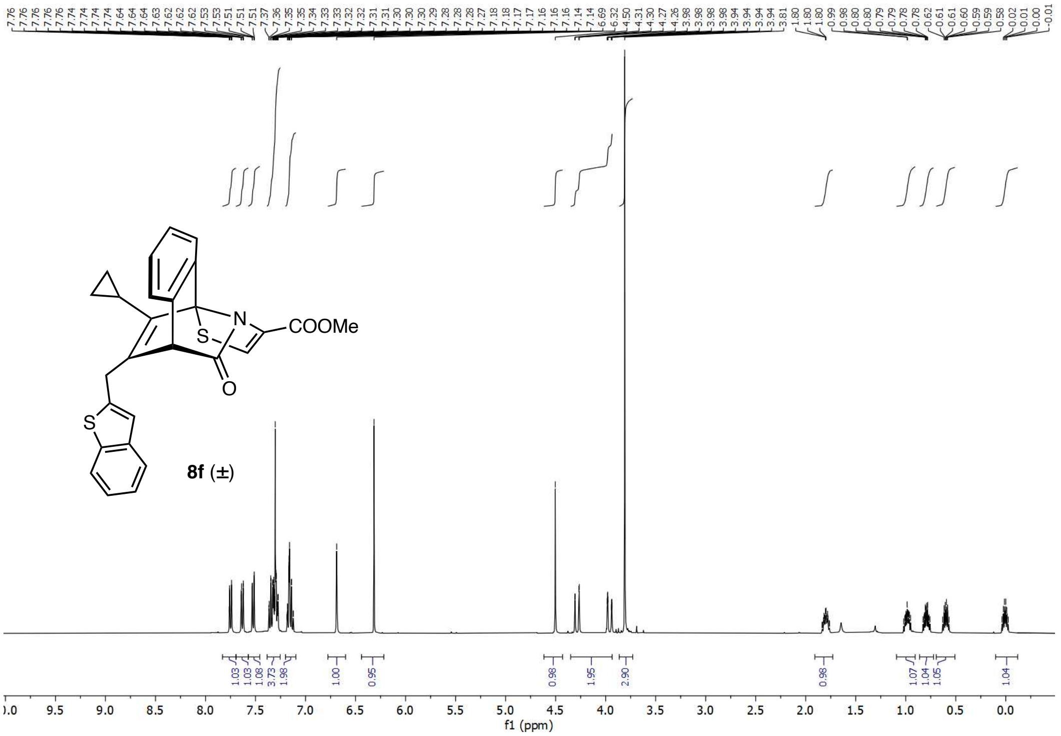

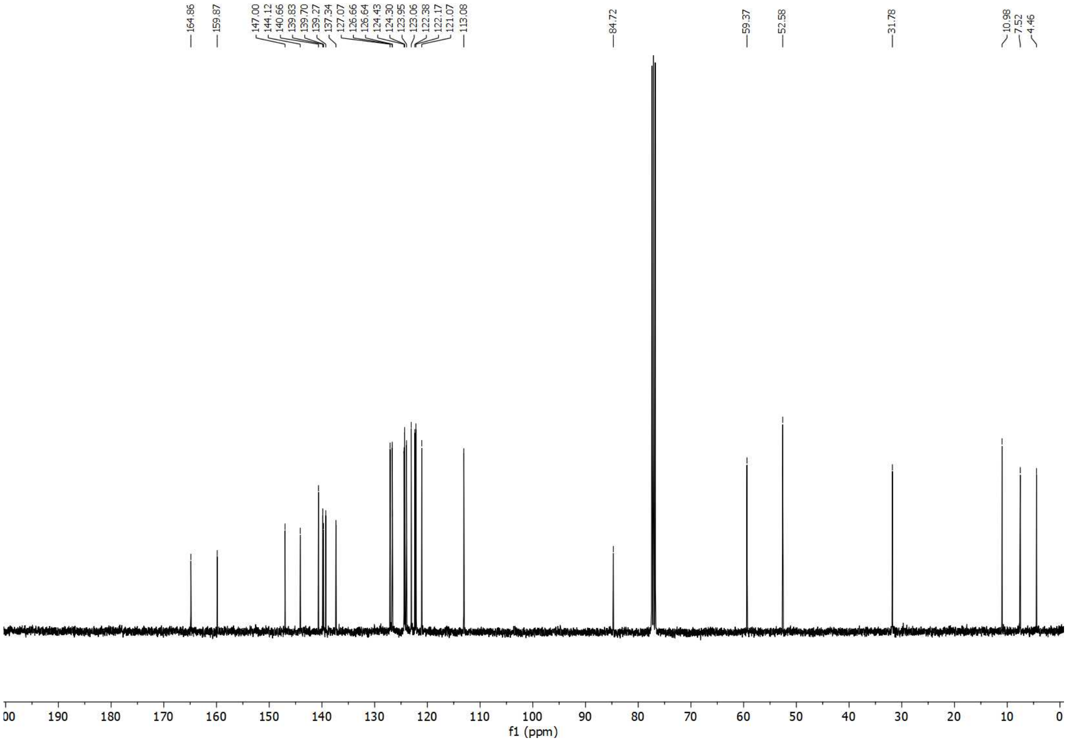

Compound 8g. ^{1}H NMR (400 MHz, $CDCl_3$) $^{13}C\{^{1}H\}$ NMR (100 MHz, $CDCl_3$)

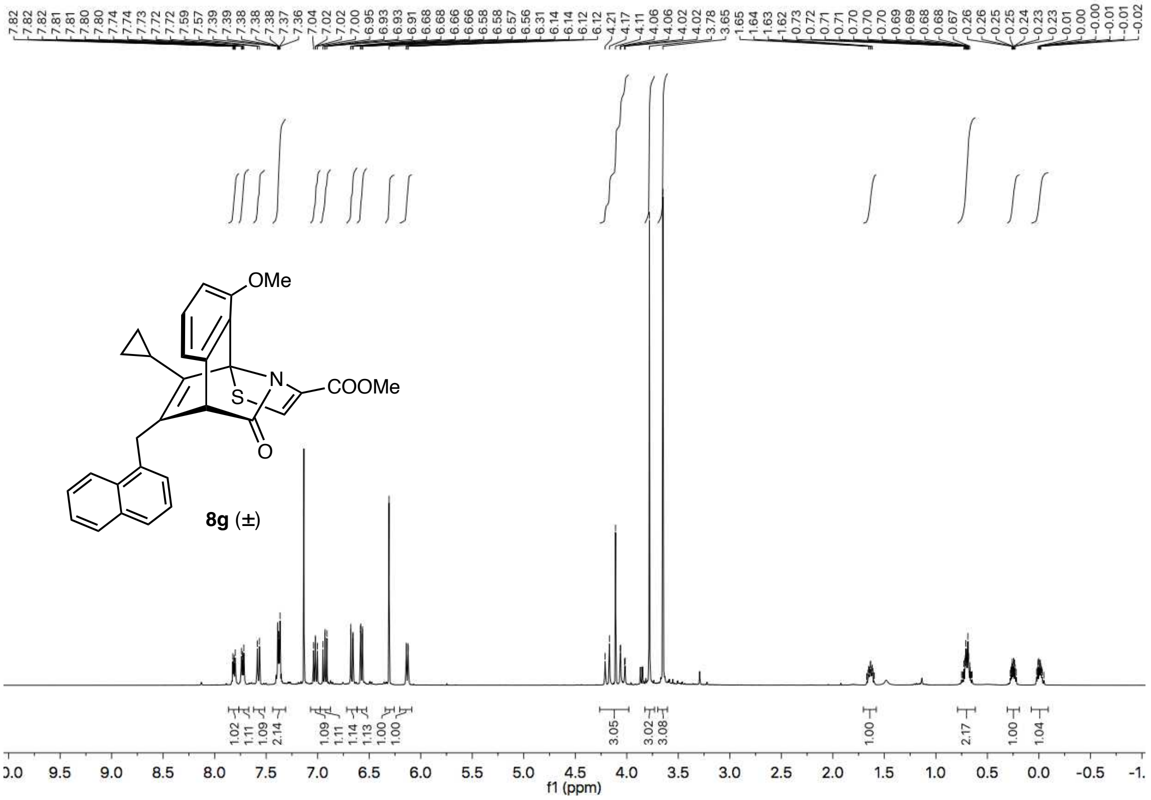

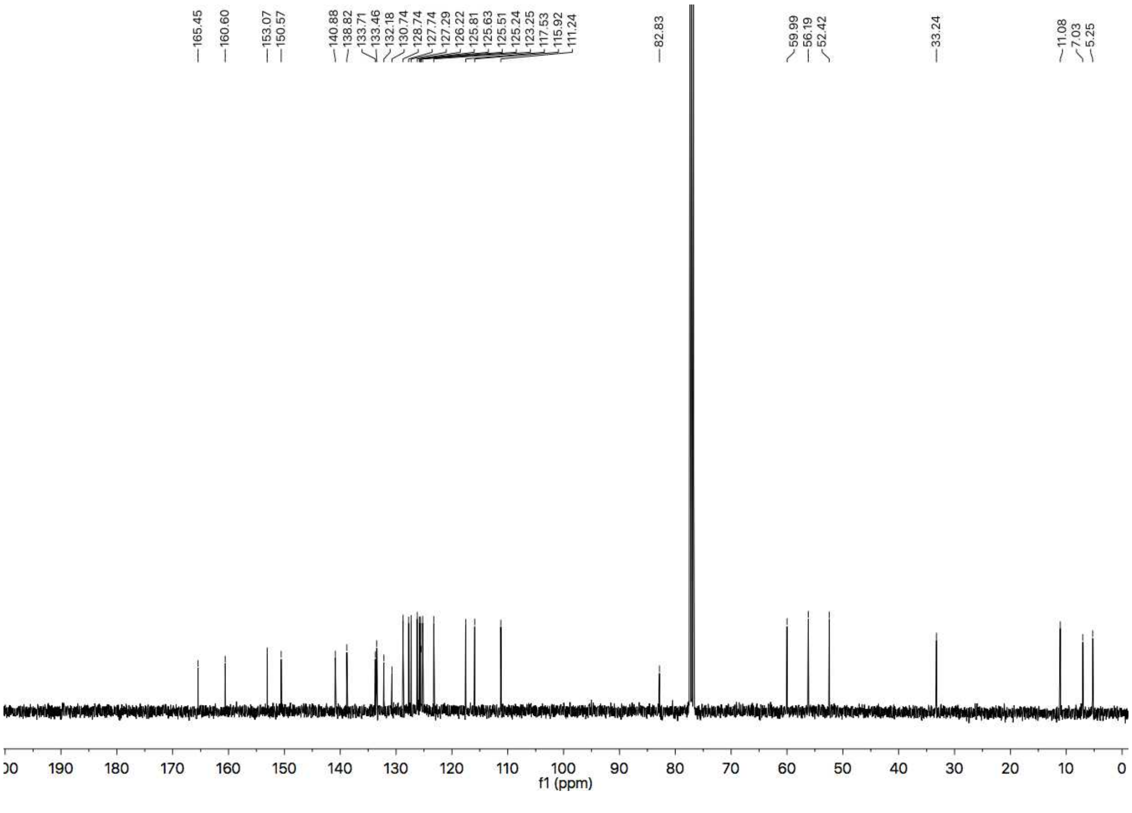

Compound 8h. ^{1}H NMR (400 MHz, $CDCl_3$) $^{13}C\{^{1}H\}$ NMR (100 MHz, $CDCl_3$)

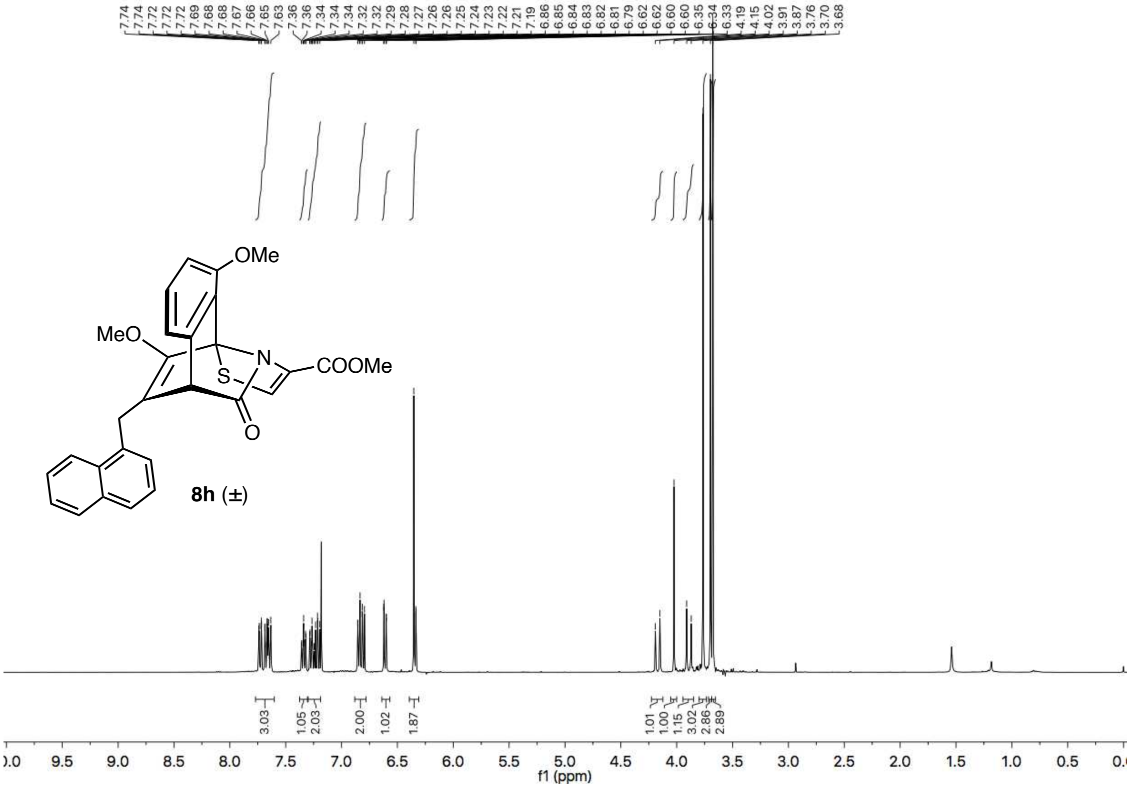

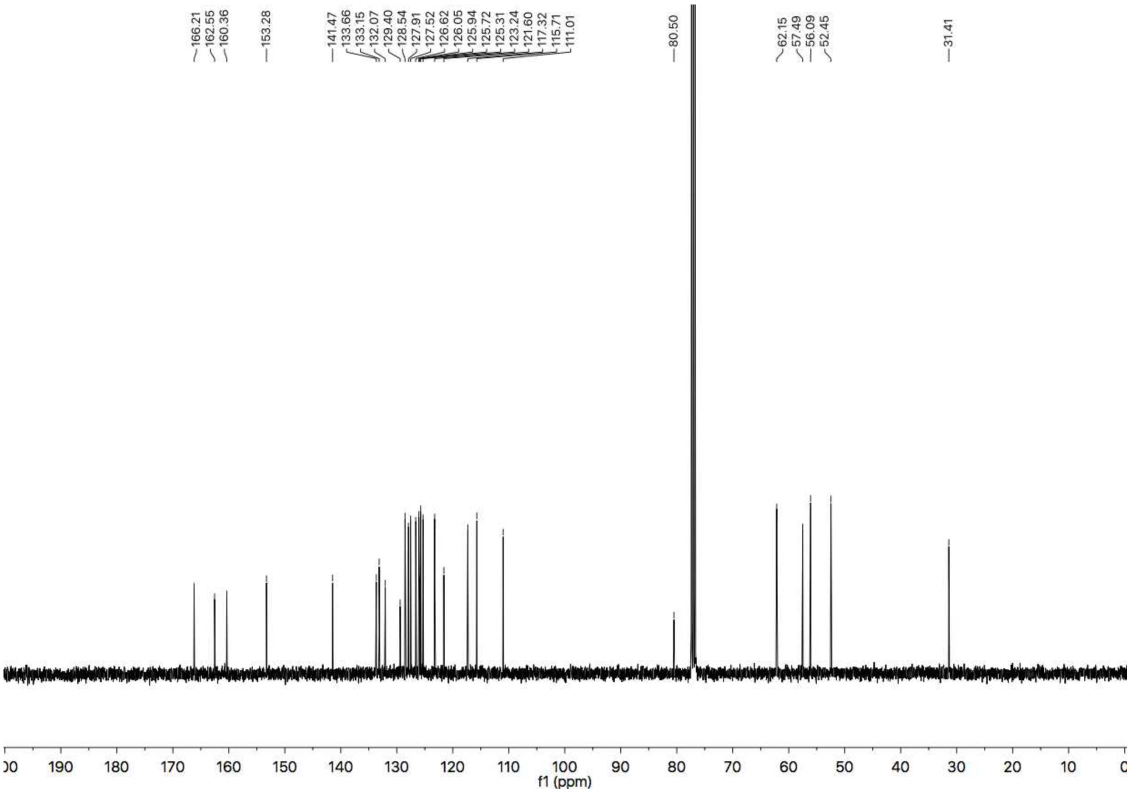

Compound 8i. ^{1}H NMR (400 MHz, $CDCl_3$) ^{13}C{^{1}H} NMR (100 MHz, $CDCl_3$)

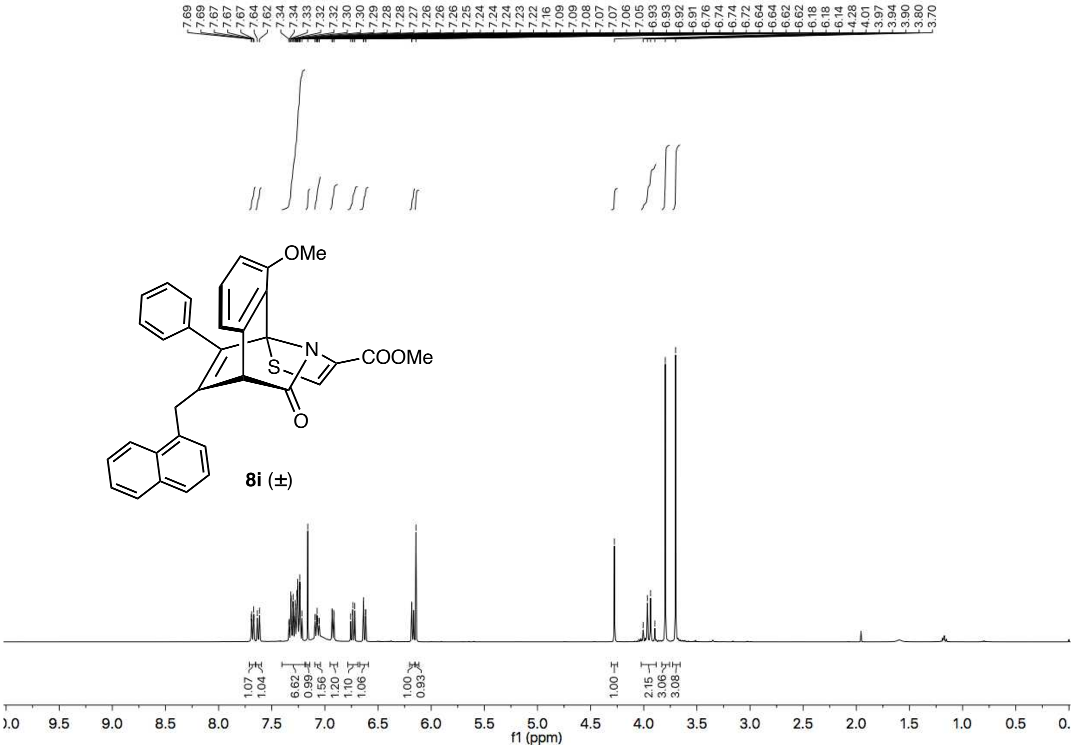

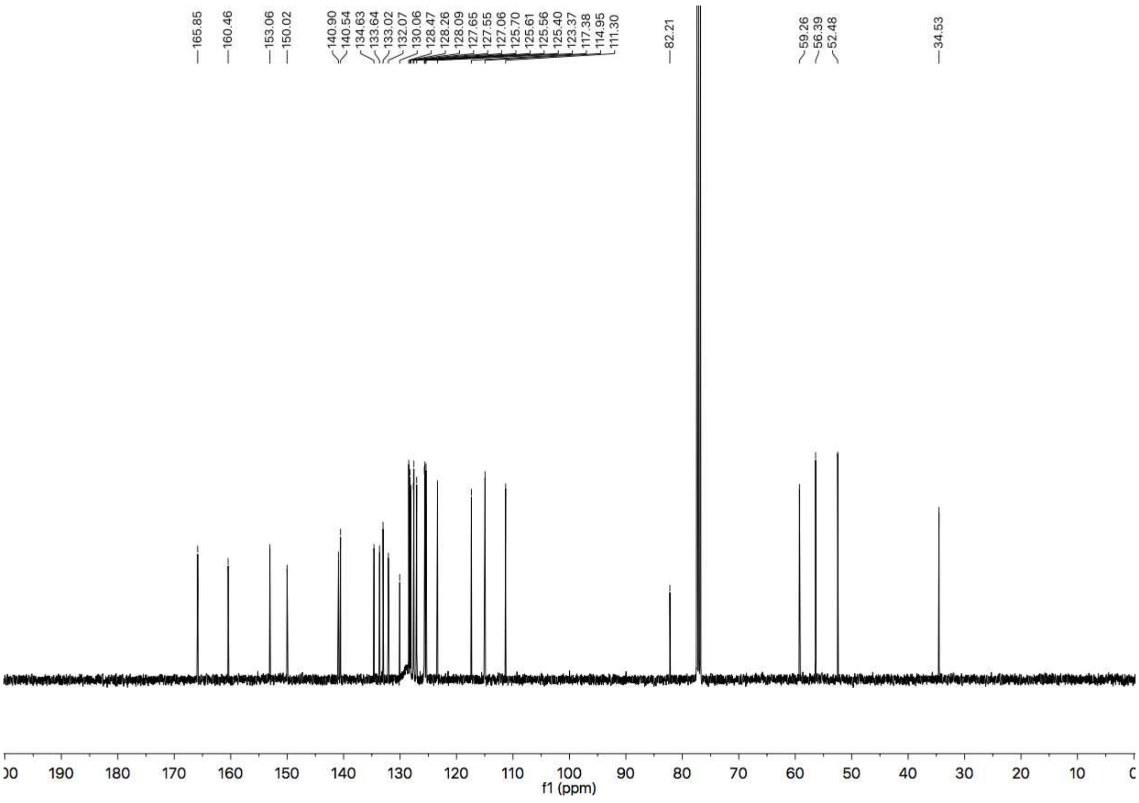

Compound 9a. ^{1}H NMR (600 MHz, $CDCl_3$) $^{13}C\{^{1}H\}$ NMR (151 MHz, $CDCl_3$)

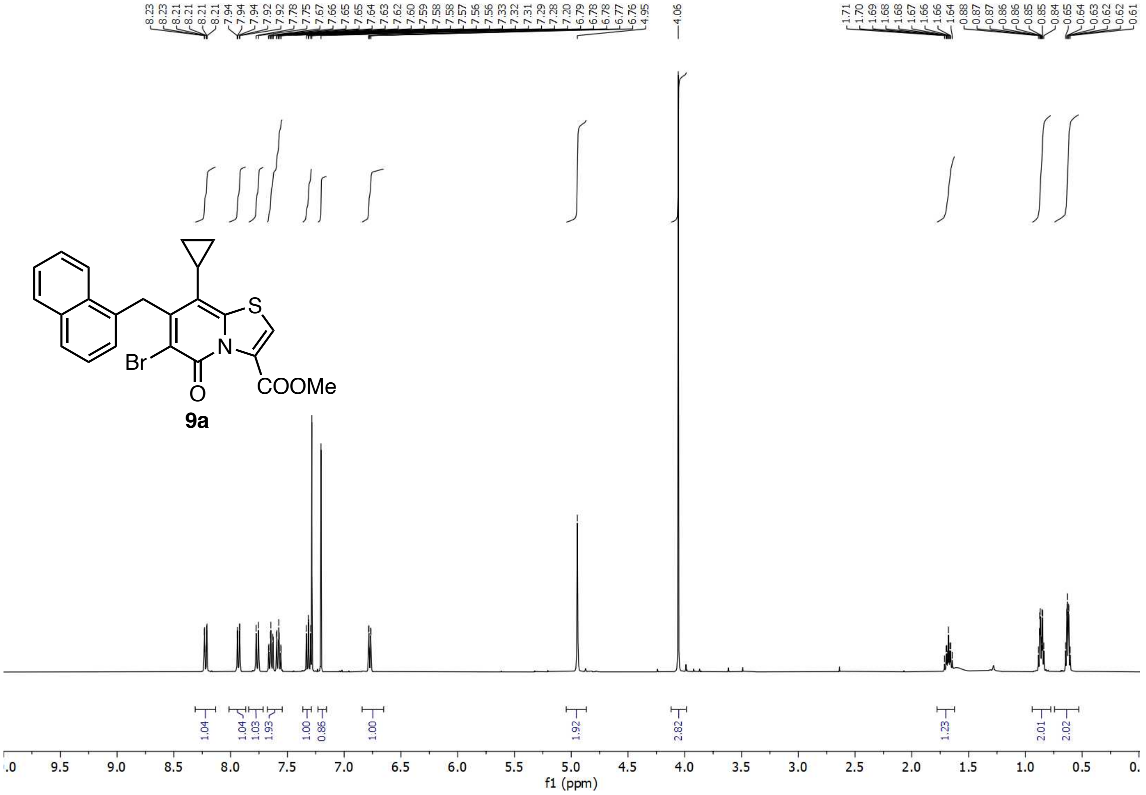

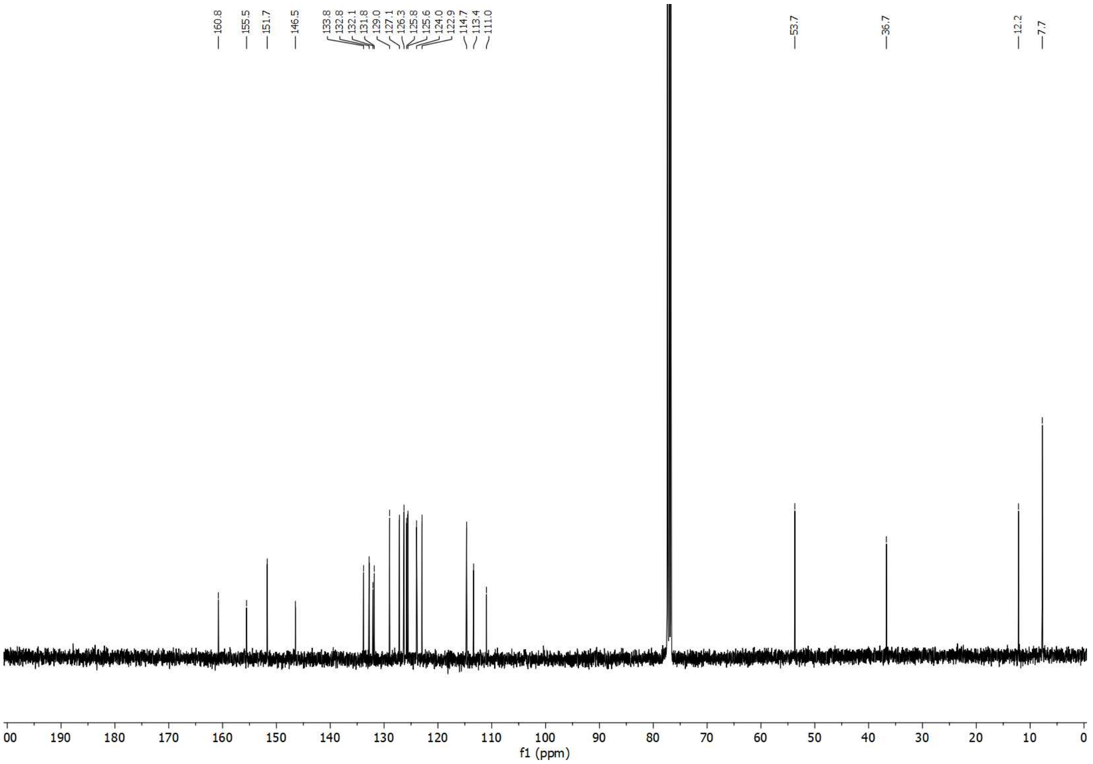

Compound 9b. ^{1}H NMR (600 MHz, $CDCl_3$) $^{13}C\{^{1}H\}$ NMR (151 MHz, $CDCl_3$)

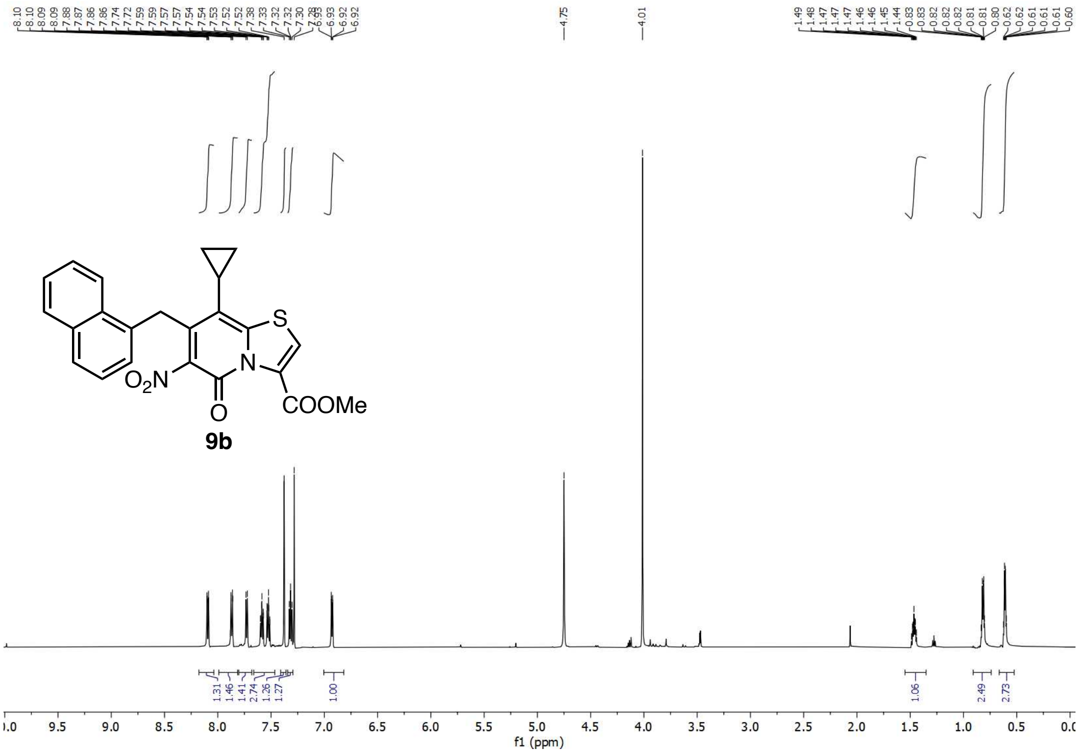

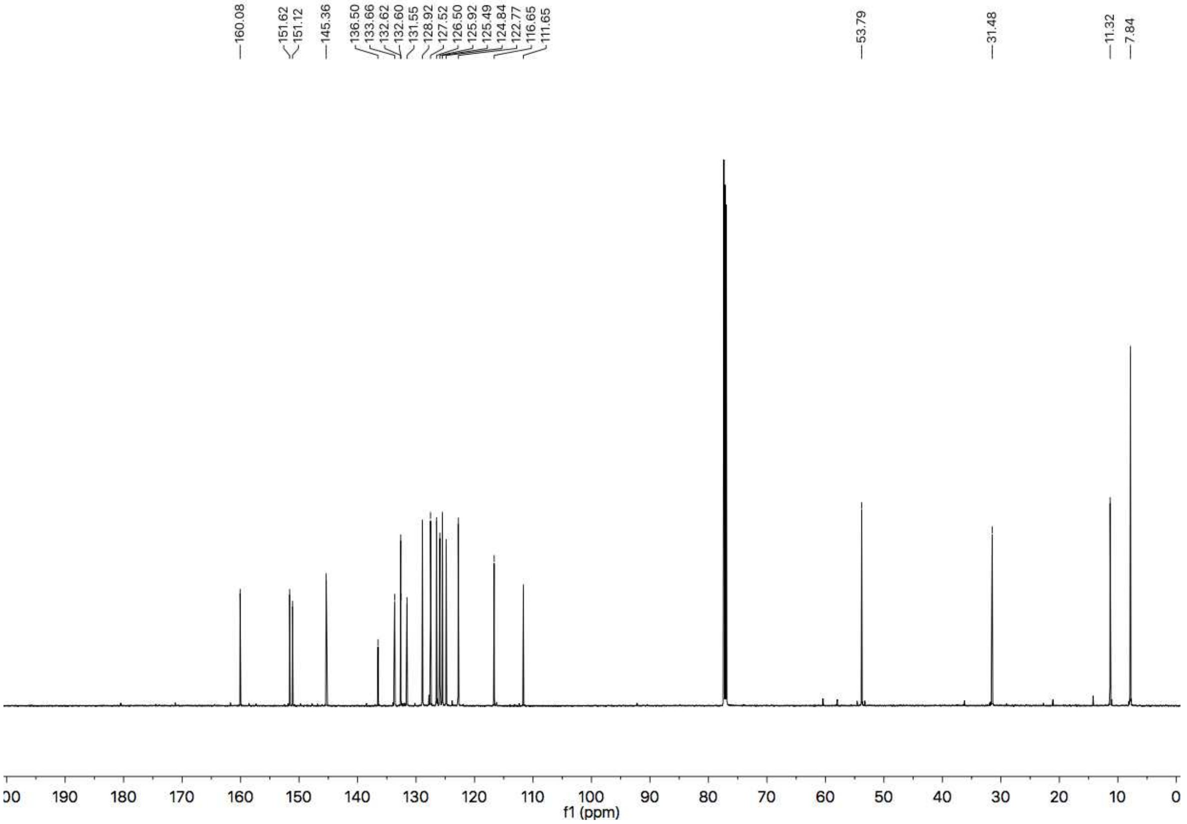

Compound 9c. ^{1}H NMR (400 MHz, $CDCl_3$) $^{13}C\{^{1}H\}$ NMR (100 MHz, $CDCl_3$)

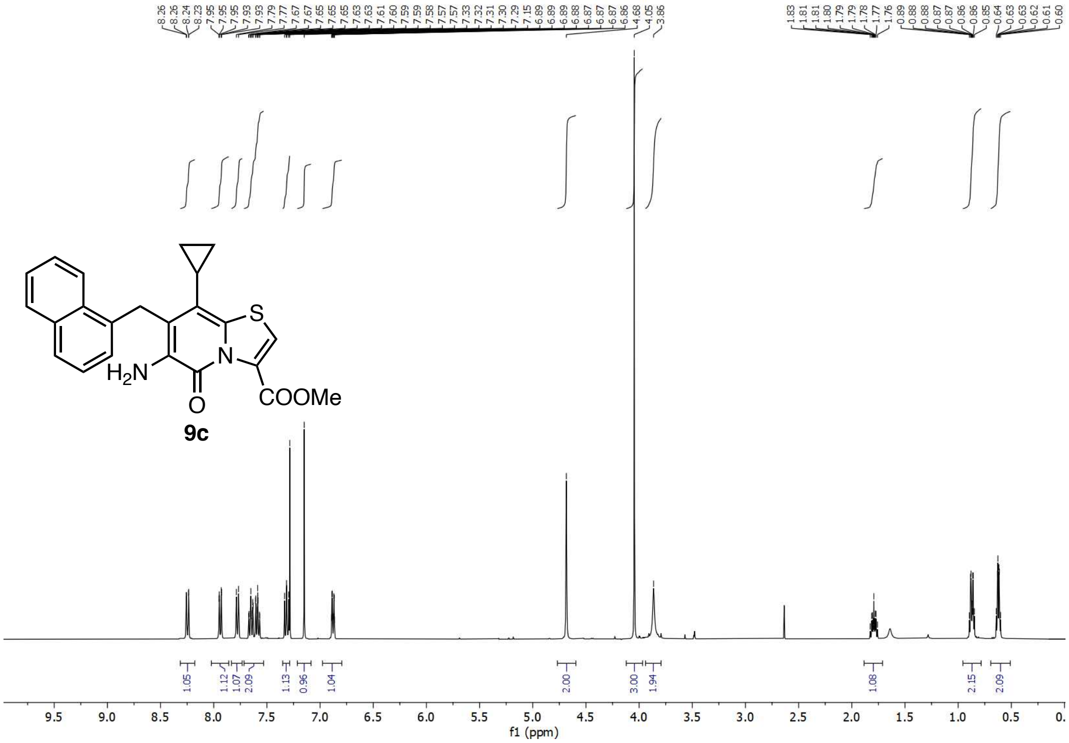

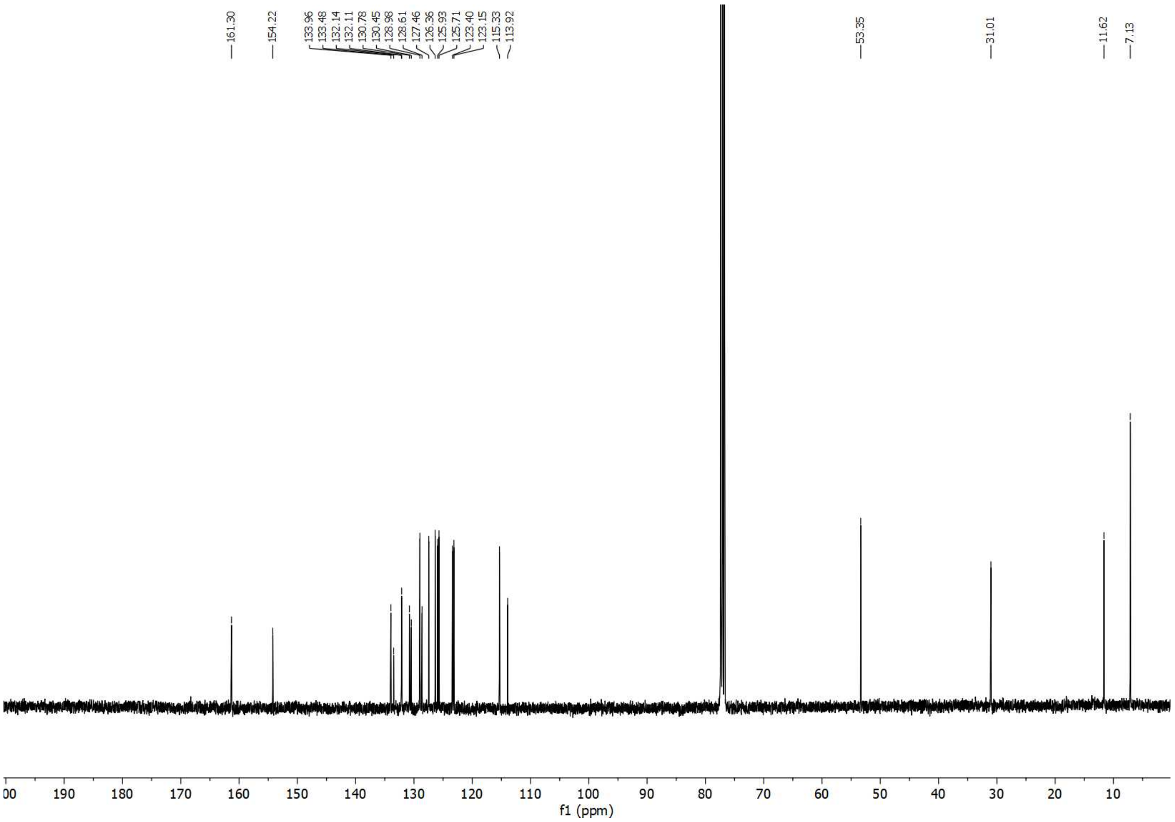

Compound 10a. ^{1}H NMR (400 MHz, $CDCl_3$) $^{13}C\{^{1}H\}$ NMR (100 MHz, $CDCl_3$)

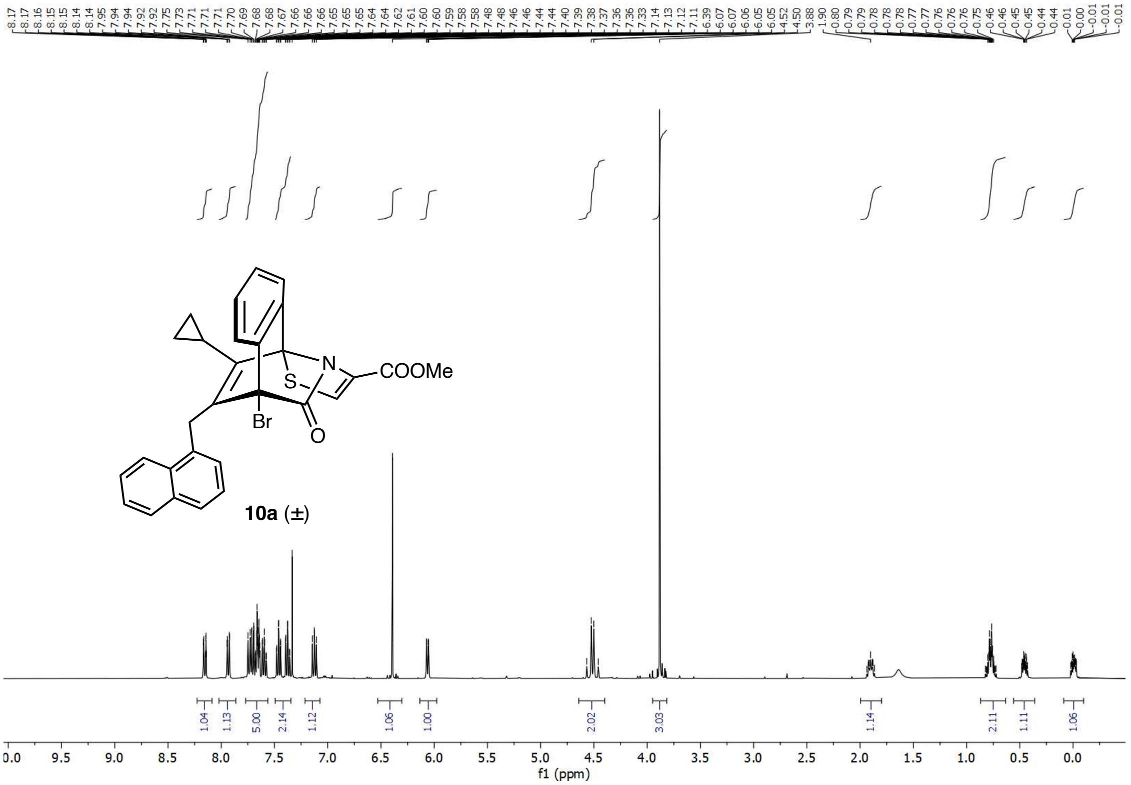

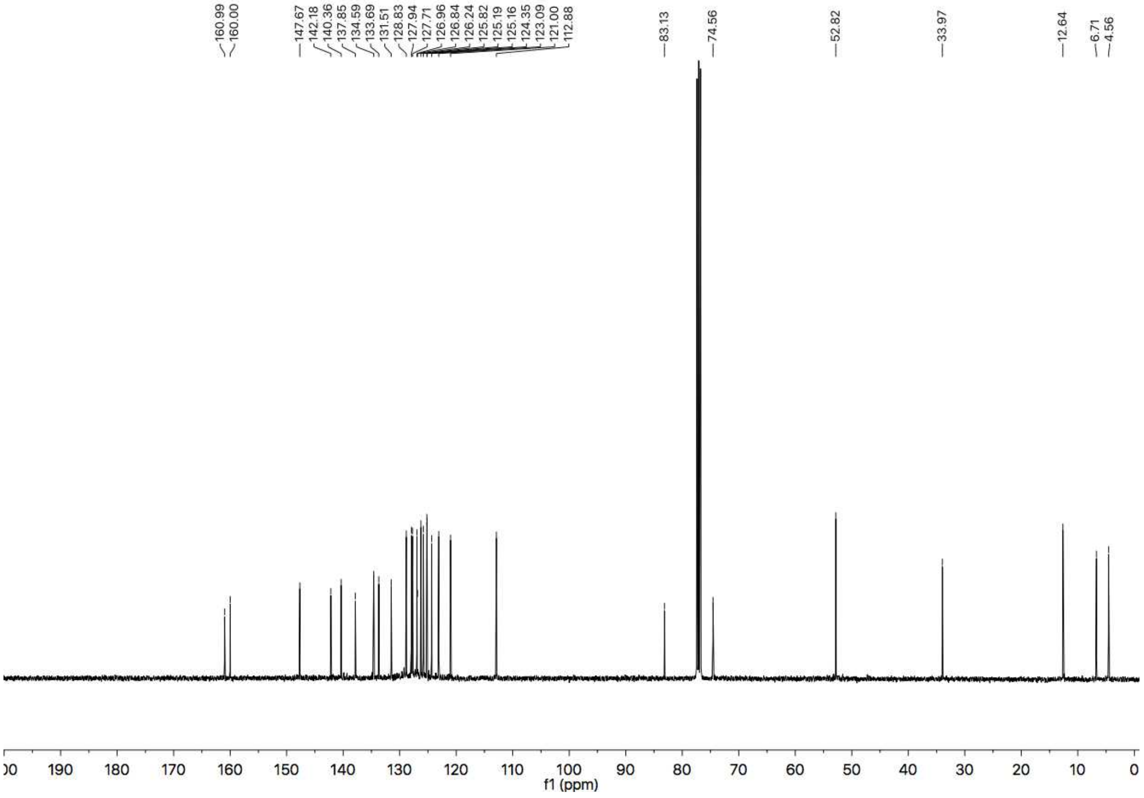

Compound 10c. ^{1}H NMR (400 MHz, $CDCl_3$) $^{13}C\{^{1}H\}$ NMR (100 MHz, $CDCl_3$)

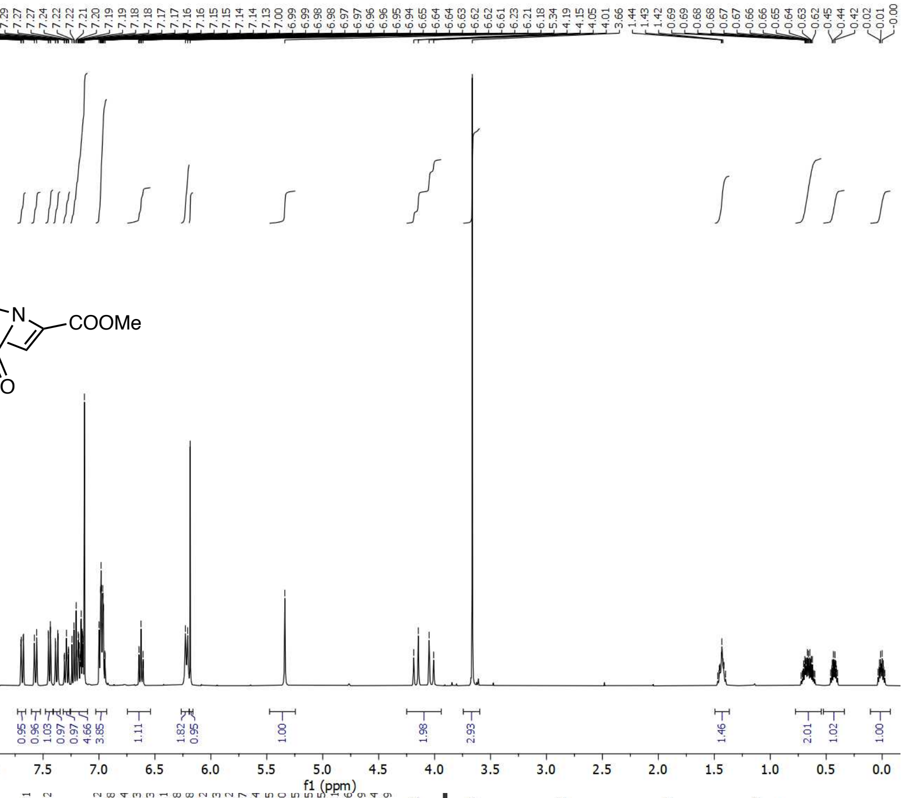

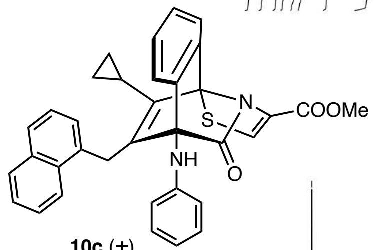

10c (±)

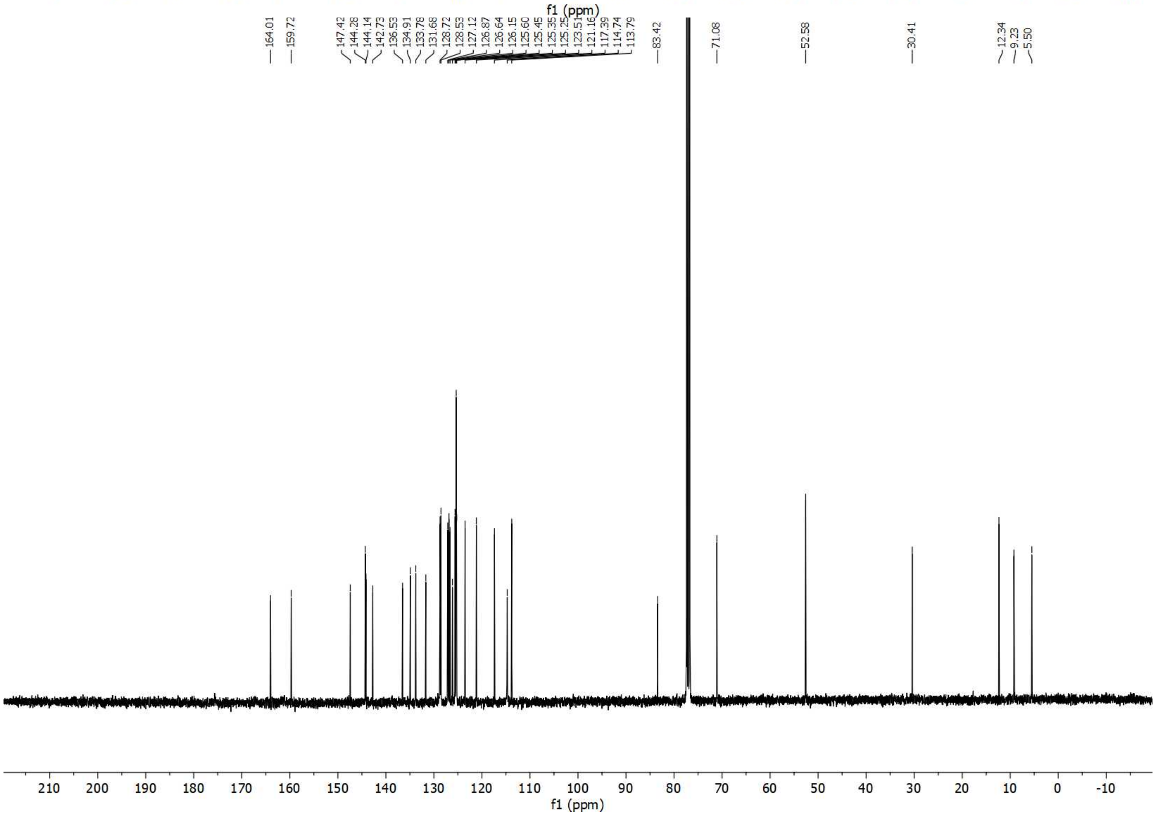

Compound 11. ^{1}H NMR (400 MHz, $CDCl_3$) $^{13}C\{^{1}H\}$ NMR (100 MHz, $CDCl_3$)

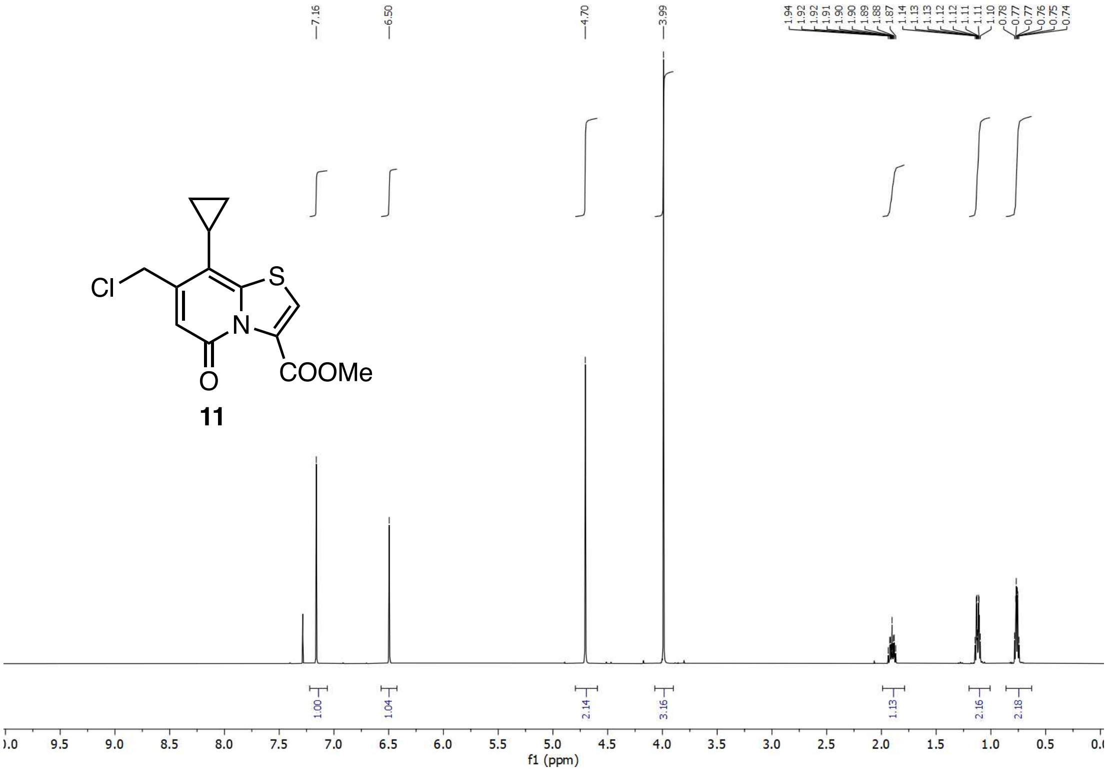

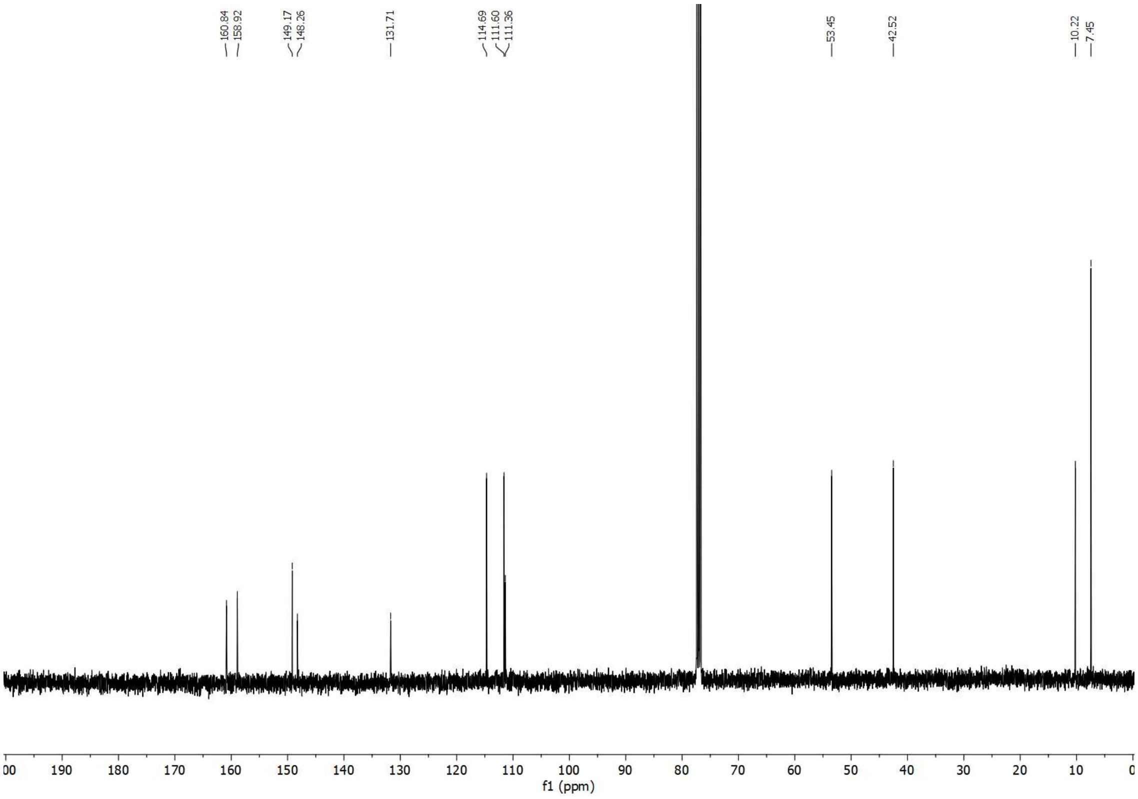

Compound 12. ^{1}H NMR (400 MHz, $CDCl_3$) $^{13}C\{^{1}H\}$ NMR (100 MHz, $CDCl_3$)

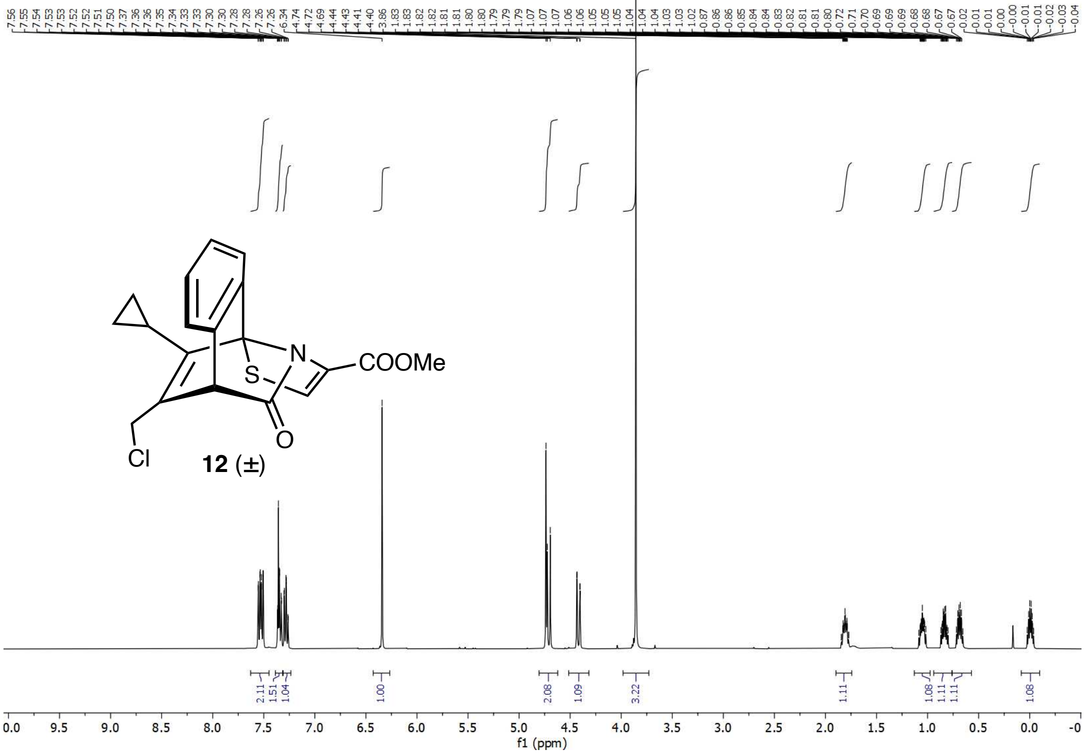

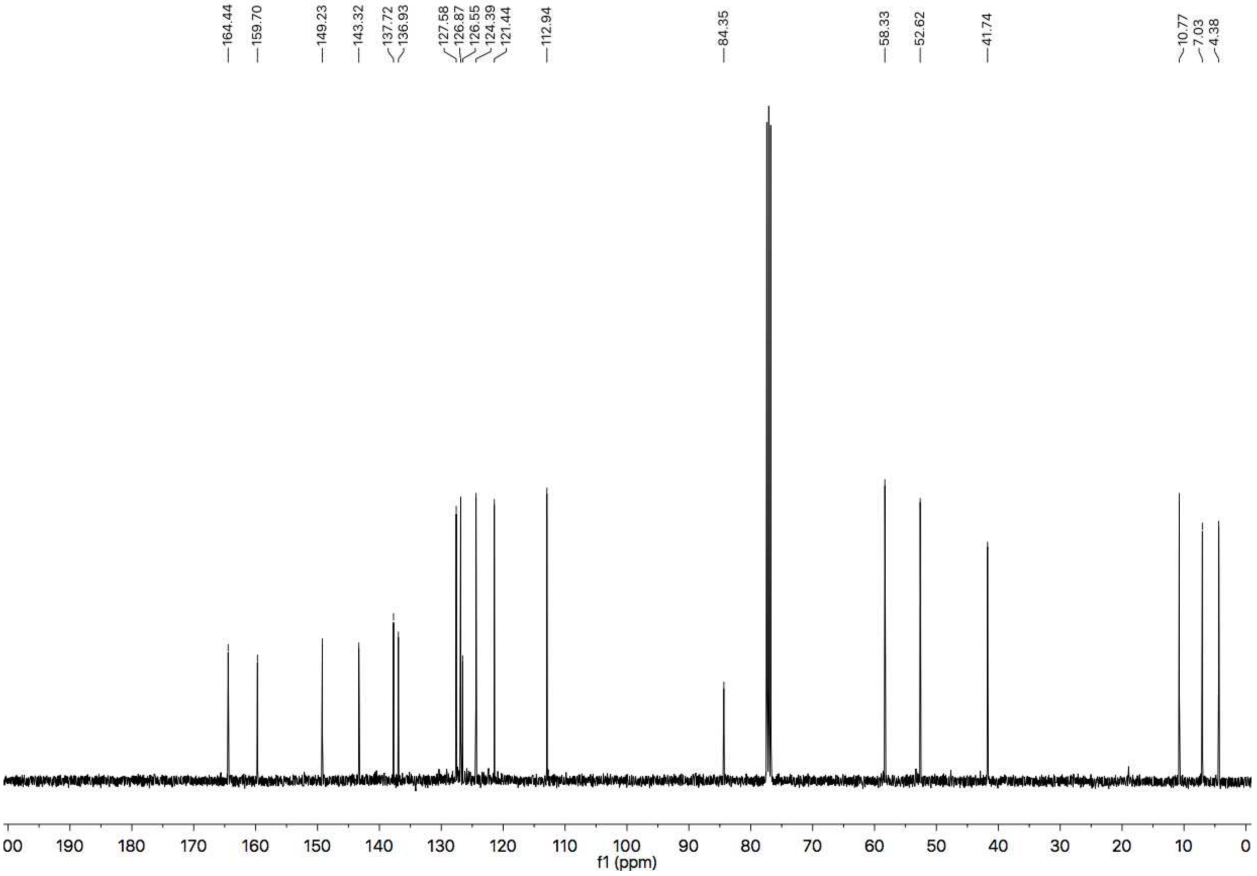

Compound 13. ^{1}H NMR (600 MHz, $CDCl_3$) $^{13}C\{^{1}H\}$ NMR (151 MHz, $CDCl_3$)

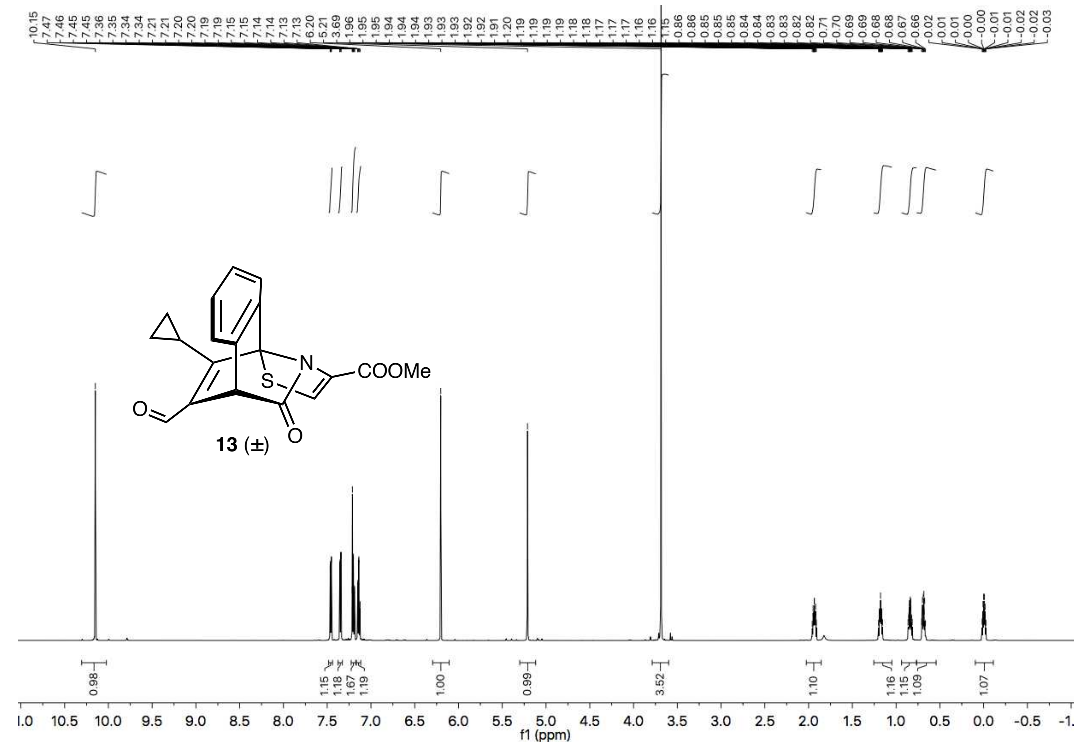

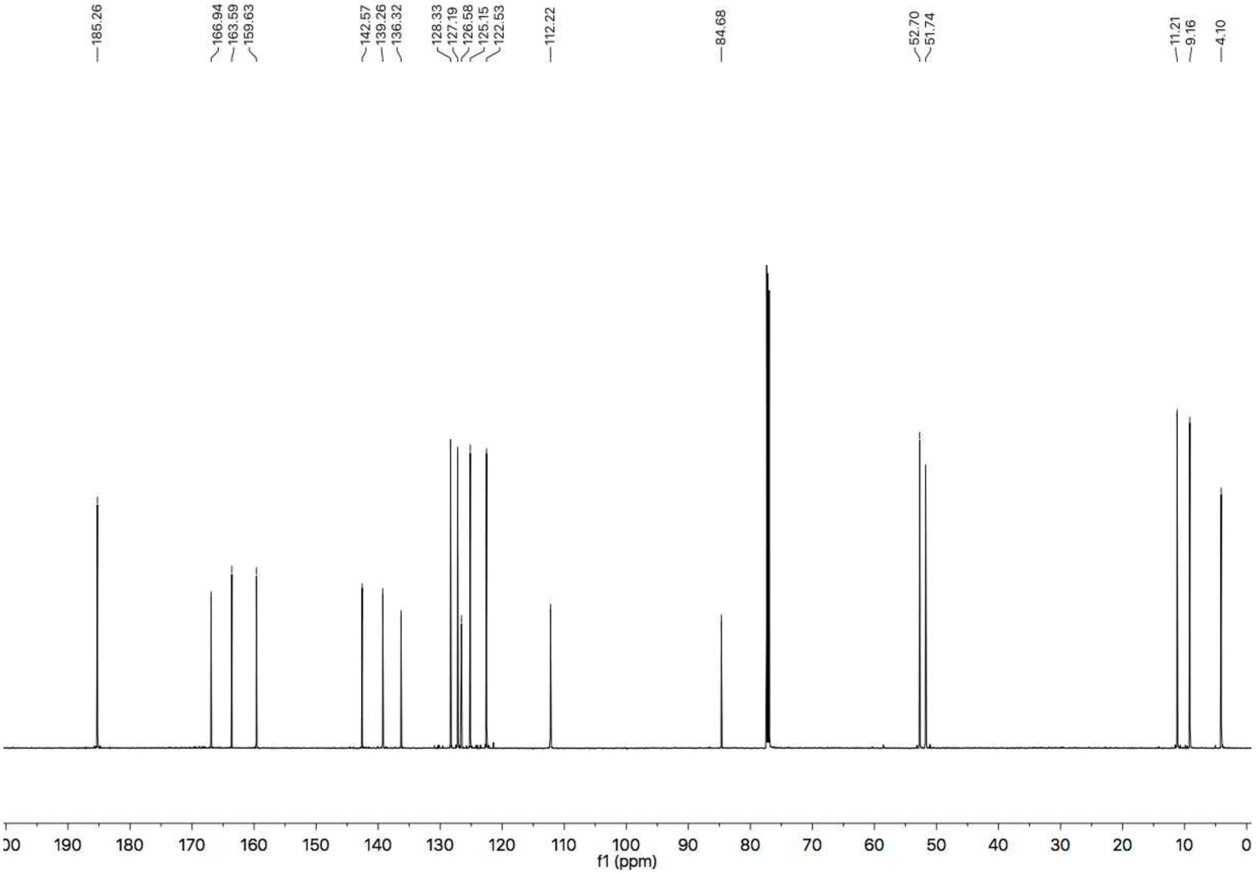

Compound 14. ^{1}H NMR (400 MHz, $CDCl_3$) $^{13}C\{^{1}H\}$ NMR (100 MHz, $CDCl_3$)

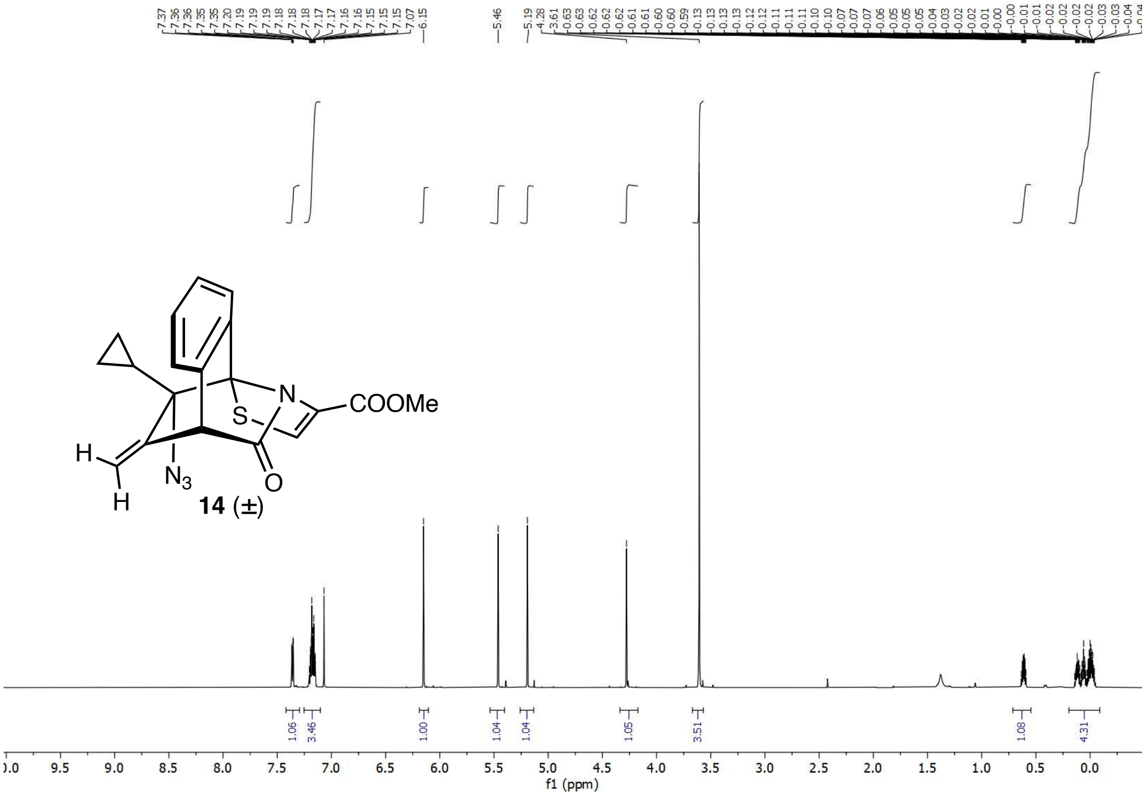

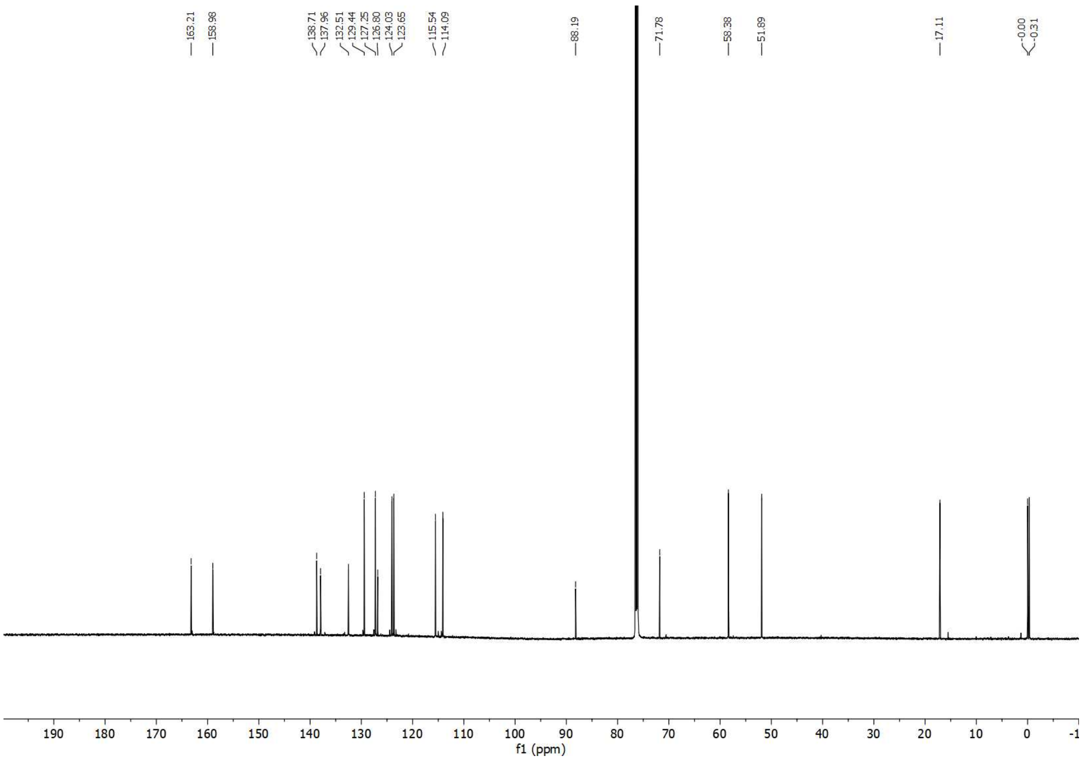

Compound 15. ^{1}H NMR (400 MHz, $CDCl_3$) $^{13}C\{^{1}H\}$ NMR (100 MHz, $CDCl_3$)

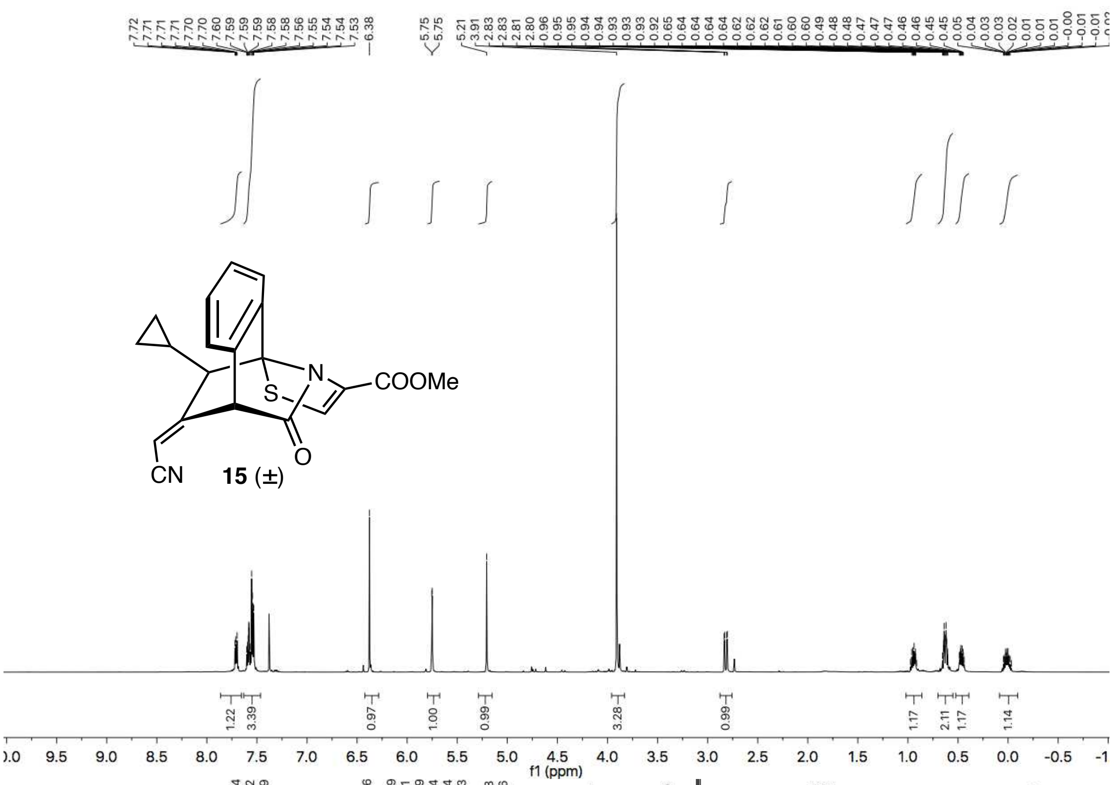

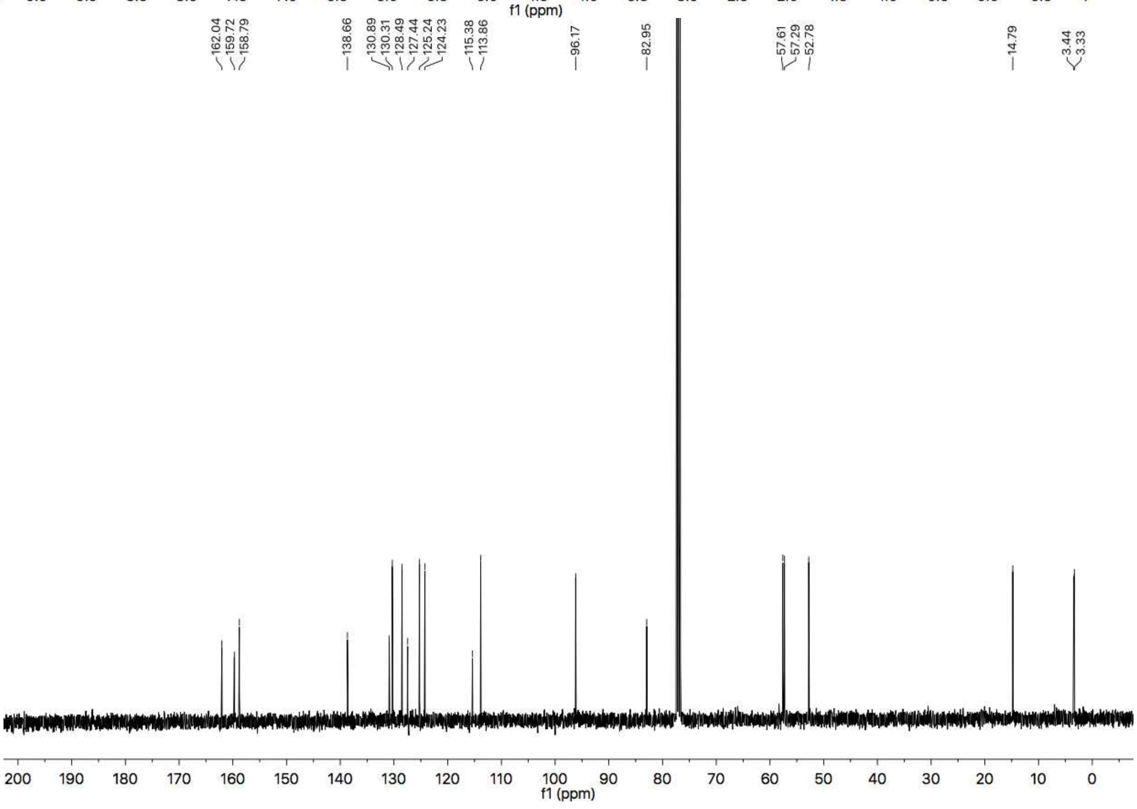

5. References

1. Aberg, V.; Norman, F.; Chorell, E.; Westermark, A.; Olofsson, A.; Sauer-Eriksson, A. E.; Almqvist, F., Microwave-assisted decarboxylation of bicyclic 2-pyridone scaffolds and identification of Abeta-peptide aggregation inhibitors. *Org Biomol Chem* **2005,** *3*, 2817-23.
2. Emtenas, H.; Taflin, C.; Almqvist, F., Efficient microwave assisted synthesis of optically active bicyclic 2-pyridinones via delta2-thiazolines. *Mol Divers* **2003,** *7*, 165-9.
3. Good, J. A.; Silver, J.; Nunez-Otero, C.; Bahnan, W.; Krishnan, K. S.; Salin, O.; Engstrom, P.; Svensson, R.; Artursson, P.; Gylfe, A.; Bergstrom, S.; Almqvist, F., Thiazolino 2-Pyridone Amide Inhibitors of Chlamydia trachomatis Infectivity. *J Med Chem* **2016,** *59*, 2094-108.
4. Kulen, M.; Lindgren, M.; Hansen, S.; Cairns, A. G.; Grundstrom, C.; Begum, A.; van der Lingen, I.; Brannstrom, K.; Hall, M.; Sauer, U. H.; Johansson, J.; Sauer-Eriksson, A. E.; Almqvist, F., Structure-Based Design of Inhibitors Targeting PrfA, the Master Virulence Regulator of Listeria monocytogenes. *J Med Chem* **2018,** *61*, 4165-4175.
5. Bengtsson, C.; Almqvist, F., Regioselective halogenations and subsequent Suzuki-Miyaura coupling onto bicyclic 2-pyridones. *J Org Chem* **2010,** *75*, 972-5.
6. Chorell, E.; Edvinsson, S.; Almqvist, F., Improved procedure for the enantioselective synthesis of dihydrooxazolo and dihydrothiazolo ring-fused 2-pyridones. *Tetrahedron Lett* **2010,** *51*, 2461-2463.
7. Chorell, E.; Pinkner, J. S.; Phan, G.; Edvinsson, S.; Buelens, F.; Remaut, H.; Waksman, G.; Hultgren, S. J.; Almqvist, F., Design and synthesis of C-2 substituted thiazolo and dihydrothiazolo ring-fused 2-pyridones: pilicides with increased antivirulence activity. *J Med Chem* **2010,** *53*, 5690-5.

(8) Crysalis CCD; Oxford Diffraction Ltd.: Abingdon, O., UK, 2005.

(9) Crysalis RED; Oxford Diffraction Ltd.: Abingdon, O., UK, 2005.

(10) a) Sheldrick, G. M., Crystal structure refinement with SHELXL. *Acta Cryst. Sect. C, Structural Chemistry* 2015, *71*, 3-8. b) Dolomanov, O. V.; Bourhis, L. J.; Howard, J. A. K.; Puschmann, H. *J. Appl. Cryst.*, **2009,** 42, 339-341.

(11) CrystalMaker Software; Begbroke Science Park, S. L., Yarnton, Oxfordshire, OX5 1PF, United Kingdom, 2010.
